# Novel seleno-ureido hybrids targeting head and neck cancer and beyond: design, synthesis, and apoptosis-mediated anticancer evaluation

**DOI:** 10.1039/d6ra03577a

**Published:** 2026-07-15

**Authors:** Saad Shaaban, Samia S. Hawas, Ohud alzaidi, Marwa Sharaky, Fatema S. Alatawi, Khadra B. Alomari, Zainab S. Alghamdi, Hussein Ba-Ghazal, Arwa Omar Al Khatib, Hany M. Abd El-Lateef, Tarek A. Yousef, Mohamed Alaasar, Ahmed A. Al-Karmalawy

**Affiliations:** a Department of Chemistry, College of Science, King Faisal University Al-Ahsa 31982 Saudi Arabia sibrahim@kfu.edu.sa; b Department of Pharmaceutical Chemistry, Faculty of Pharmacy, Horus University-Egypt New Damietta 34518 Egypt akarmalawy@horus.edu.eg; c Department of Chemistry, ‏College of Science-Al Khurma, Taif University Taif 21944 Saudi Arabia; d Cancer Biology Department, National Cancer Institute (NCI), Cairo University Cairo Egypt; e Department of Biochemistry, Faculty of Science, University of Tabuk Tabuk Saudi Arabia; f Jazan University, Department of Physical Sciences, Chemistry Division Jazan 45142 Kingdom of Saudi Arabia; g Department of Chemistry, College of Science, Imam Abdulrahman Bin Faisal University Dammam 31441 Saudi Arabia; h Faculty of Pharmacy, Al-Ahliyya Amman University Amman Jordan; i College of Science, Chemistry Department, Imam Mohammad Ibn Saud Islamic University (IMSIU) Riyadh 11623 Saudi Arabia; j Department of Chemistry, Faculty of Science, Cairo University Giza Egypt

## Abstract

This study systematically designed a series of urea-linked organoselenium hybrids *via* molecular hybridization, combining the redox-active selenium pharmacophore with the hydrogen-bond-forming urea motif. The synthesized compounds featured both *mono*- and *bis*-ureido frameworks with various selenium substituents (methyl, allyl, and benzyl) and differing linker topologies. A biological evaluation of a panel of eight human tumor cells exhibited promising antiproliferative activity, with average growth inhibition percentages (% GI) ranging from low to high potency. Compound HB204 possessed the greatest activity (% GI = 77.72), followed by HB188 (% GI = 76.31), which was more active than the reference drug doxorubicin (% GI = 61.89). An analysis of the structure–activity relationship revealed that *mono*-ureido scaffolds, allylselanyl substitution, and hydrophobic linker frameworks significantly enhance anticancer activity. More cytotoxicity tests showed that HB204 worked better, with an IC_50_ of 5.68 µM against the FaDu cancer cell. Mechanistic investigations revealed that HB204 promotes apoptosis by increasing pro-apoptotic proteins (BAX and caspases 3, 7, and 9) and decreasing anti-apoptotic markers (BCL-2, MMP2, and MMP9). Cell cycle analysis also showed a significant arrest during the pre-G1 stage. Annexin V-FITC/PI flow cytometric analysis further confirmed apoptosis induction, increasing the total apoptotic population from 1.18% in untreated cells to 23.40% following HB204 treatment. These findings indicate that urea-linked organoselenium hybrids are promising redox-modulating anticancer agents. HB204 is the best candidate for more research into HNSCC. Molecular docking matched with the experimental results, showing that HB204 binds more strongly to BAX than the reference inhibitor (BAI 1). Additionally, HB204 and BAI 1 underwent a 500 ns MD simulation to validate the molecular docking results. This establishes important hydrogen bonding and π–hydrogen interactions that explain its pro-apoptotic function.

## Abstract 2

Novel seleno-ureido hybrids targeting head and neck cancer and beyond.

## Graphical abstract

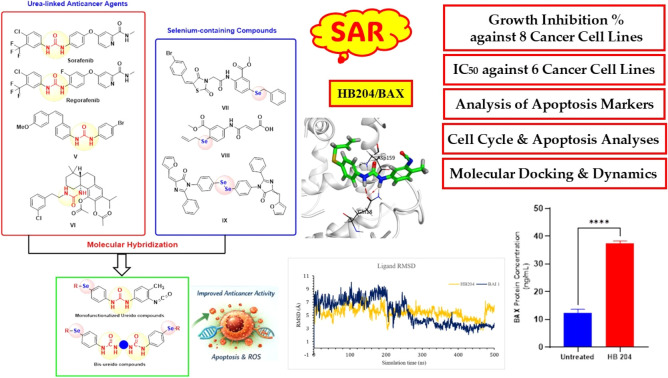

## 1. Introduction

Head and neck squamous cell carcinoma (HNSCC) is regarded as a major worldwide health issue.^1–4^ FaDu cells, derived from human hypopharyngeal carcinoma, are frequently utilized in in *vitro* studies to evaluate anticancer efficacy and investigate underlying mechanisms.^5–7^ Even with new treatments, managing HNSCC in the clinic is still very hard because of therapy resistance, tumor recurrence, and bad prognoses.^8–13^ These limitations highlight the urgent need for novel anticancer agents that can effectively modulate essential cellular pathways involved in tumor progression.^14–17^

Two of the most important ways to treat cancer are to cause apoptosis and stop the cell cycle. This is because they directly control how long tumor cells can live and how quickly they multiply.^18,19^ HNSCC is marked by prevalent dysfunctions in apoptotic signaling pathways and cell cycle checkpoints, resulting in unregulated cell proliferation and treatment resistance.^20–22^ As a result, compounds that can restore apoptotic equilibrium and halt cell cycle progression are very important in medicine.

Among apoptosis-related proteins, BAX plays a central role in the mitochondrial apoptotic pathway through promoting mitochondrial membrane permeabilization and caspase activation.^23,24^ Dysregulation of BAX-mediated apoptosis is frequently associated with tumor progression and therapeutic resistance in HNSCC.^25,26^ Since organoselenium (OSe) compounds are known to modulate oxidative stress and apoptosis-related signaling,^27^ we hypothesized that the designed selenium–urea hybrids may exert their anticancer activity through apoptosis induction involving BAX-associated pathways. Therefore, BAX was selected as a representative molecular target for the mechanistic docking investigation.

In this context, OSe compounds were presented as prospective candidates in the realm of anticancer drug discovery.^28–31^ Selenium (Se)-containing compounds have been shown to disrupt redox hemostasis in cells and induce oxidative stress *via* the generation of reactive oxygen species (ROS), potentially culminating in cellular apoptosis.^32–36^ Additionally, various Se-based compounds have exhibited the capacity to activate both intrinsic and extrinsic apoptosis pathways, including caspase-dependent mechanisms, rendering them appealing scaffolds for mechanistic anticancer investigations.^37,38^

**Fig. 1:**
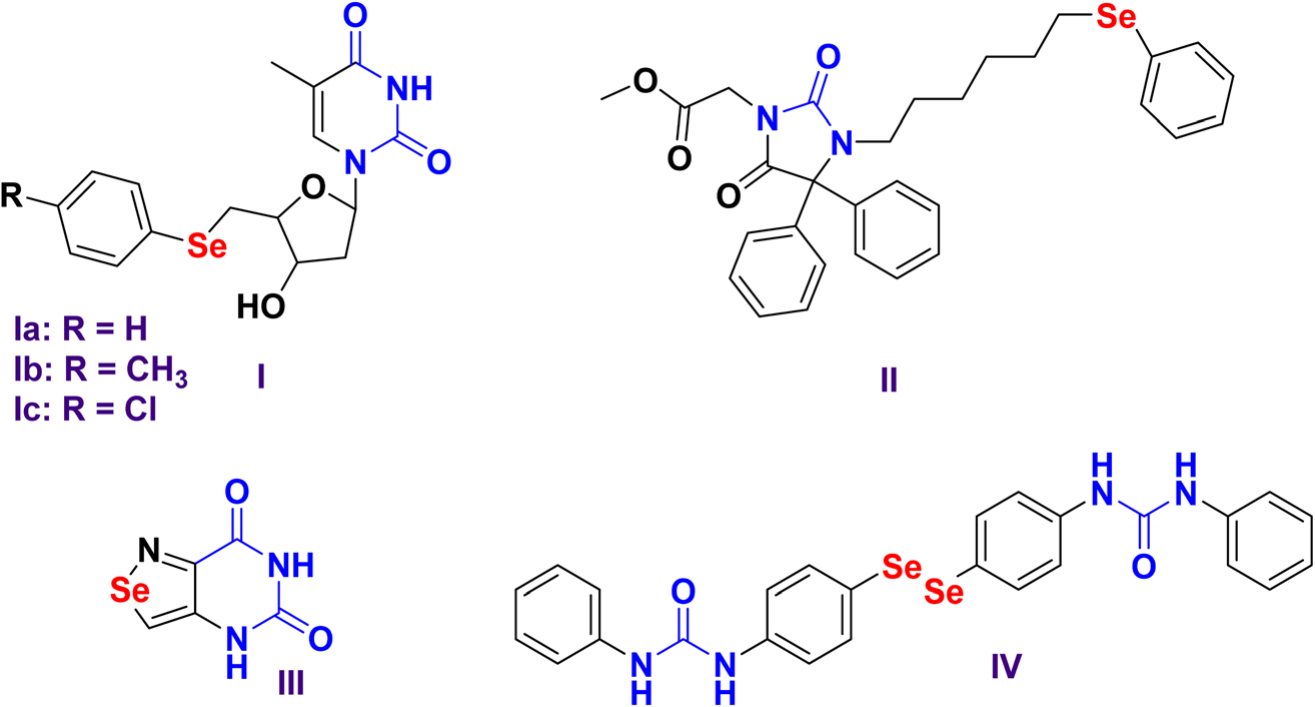
The reported organoselenium compounds connected with the urea moiety demonstrate anticancer properties.

Numerous studies have corroborated the anticancer efficacy of Se derivatives. For example, Se-substituted zidovudine derivatives (compound I) were cytotoxic to human bladder cancer 5637 cells, with IC_50_ values ranging from 40.4 to 78.85 µM.^39^ Another compound (II), which had a phenylselenide part, was very effective at stopping the growth of both sensitive and multidrug-resistant T-lymphoma cells. It had IC_50_ values of 0.67 and 0.90 µM, respectively, and altered the ABCB1 efflux pump.^40^ Moreover, compound III exhibited the capacity to induce apoptosis through the activation of caspase-8/9 and the elevation of reactive oxygen species (ROS), showing significant efficacy against melanoma (A375), breast (MCF-7), and liver (HepG2) cancer cell lines.^41,42^ Moreover, compound IV exhibited significant cytotoxicity against HepG2 cells at sub-micromolar concentrations, while demonstrating negligible toxicity towards normal cells. It induced apoptosis and inhibited the migration, invasion, and colony formation of cancer cells^43,44^ (Fig. 1).

These findings collectively highlight the potential of OSe-based compounds to modulate the survival of cancer cells, specifically through the induction of apoptosis and the disruption of proliferative signaling pathways. These characteristics render them suitable for further investigation in HNSCC models, such as FaDu cells, where their impact on apoptosis-related proteins and cell cycle regulatory pathways can be comprehensively examined.

### 1.1. Design rationale

**Fig. 2:**
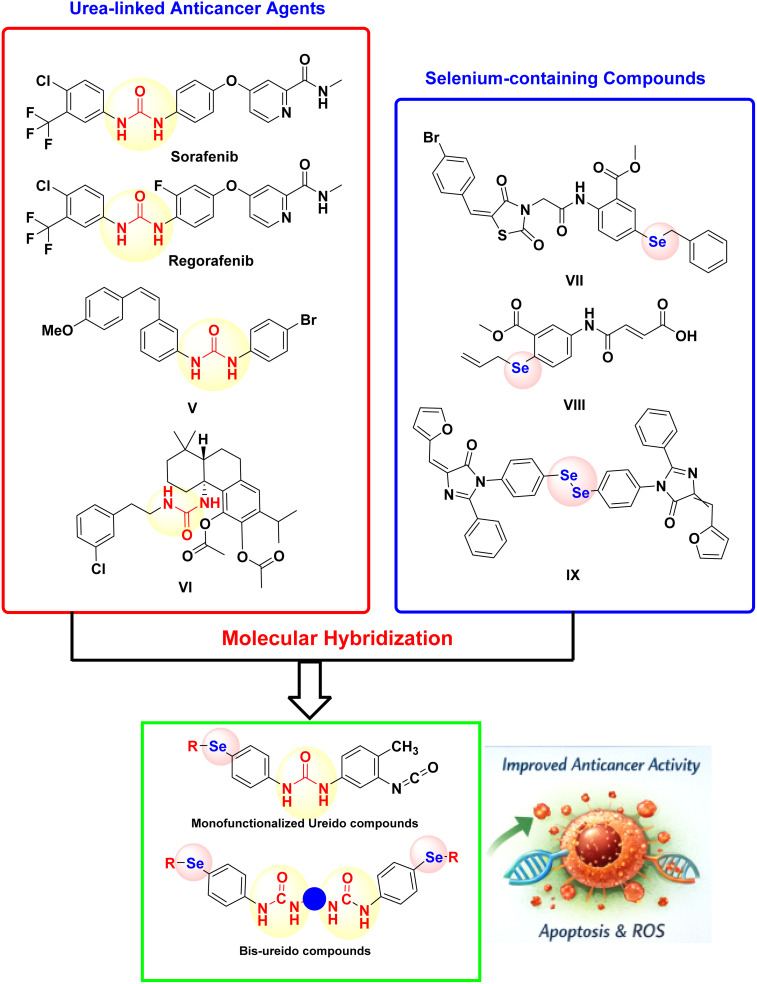
Rationale of organoselenium urea-linked derivatives.

A molecular hybridization strategy was used to develop the target compounds. This strategy aimed to combine two biologically important motifs: OSe fragments and urea-based linkers. Both of these have been proven to have anticancer properties that seem great. Urea-containing anticancer drugs like sorafenib and regorafenib show how important the urea linker is for making important hydrogen-bonding interactions take place in kinase active sites. This increases binding affinity and biological activity.^45^ Similarly, previously reported compounds (V and VI) demonstrate the significance of the urea pharmacophore in enhancing antiproliferative activity through robust interactions with targets,^40,41^ as illustrated in Fig. 2.

On the other hand, OSe compounds (VII–IX) have become more interesting because they can control cellular redox homeostasis, increase ROS production, and induce apoptosis in cancer cells.^46–48^ Se-based molecules look promising, but many of them don't have good selectivity or physicochemical properties, which could make them less useful in medicine,^49^Fig. 2.

The current design solves these problems by combining both pharmacophores into one framework. The urea functionality improves target binding by forming hydrogen bonds. The Se moiety plays a role in redox-mediated cytotoxic effects, which could lead to a stronger anticancer response through a process called molecular hybridization.^50^

The design also includes both *bis*-ureido and *mono*-ureido architectures to see how molecular symmetry and multivalency affect biological activity. The *bis*-ureido derivatives are expected to provide enhanced target engagement through dual interaction sites, whereas *mono*-ureido analogs allow evaluation of the minimal structural requirements for activity.^51^

In addition, structural diversification was achieved through variation of the Se substituents (methyl, allyl, and benzyl groups), aiming to modulate lipophilicity, steric factors, and electronic properties, which are critical determinants of cellular uptake, target interaction, and overall anticancer efficacy.^52^

The methylselanyl, allylselanyl, and benzylselanyl substituents were deliberately selected to provide gradual structural and physicochemical variation in steric bulk, lipophilicity, flexibility, and electronic properties around the Se center. The methylselanyl group represents a small hydrophobic substituent with minimal steric demand, allowing evaluation of the intrinsic contribution of the Se pharmacophore. In contrast, the allylselanyl moiety introduces conformational flexibility and moderate lipophilicity together with a π-system that may enhance hydrophobic and non-covalent interactions with biological targets. The benzylselanyl substituent was incorporated as a bulkier aromatic hydrophobic fragment to investigate the influence of increased steric size and aromaticity on antiproliferative activity and cellular uptake.

The *para*-amino arrangement was specifically selected to provide a more favorable electronic and steric environment for urea bond formation while maintaining a relatively linear molecular geometry that could facilitate target interactions. In contrast, *ortho*-substituted analogues were expected to suffer from steric hindrance near the amino functionality, potentially affecting both synthetic accessibility and biological interactions, whereas *meta*-substitution may disrupt the desired spatial orientation between the Se pharmacophore and the urea linker. Therefore, the present study focused on *para*-substituted derivatives as an initial proof-of-concept scaffold. Although the present work focused on a limited set of Se substituents, this preliminary library was intentionally designed to establish initial structure–activity relationships. Future investigations will explore broader electronic variations, including electron-donating and electron-withdrawing substituents such as halogens and heteroaromatic analogues, to further optimize the biological activity and physicochemical properties.

Collectively, this rational design integrates insights from both urea-based anticancer agents and Se-containing cytotoxic agents, and is therefore expected to yield novel compounds with improved antiproliferative activity and mechanistic diversity, justifying their synthesis and subsequent biological evaluation (Fig. 2).

## 2. Results and discussion

### 2.1. Chemistry

**Scheme 1:**
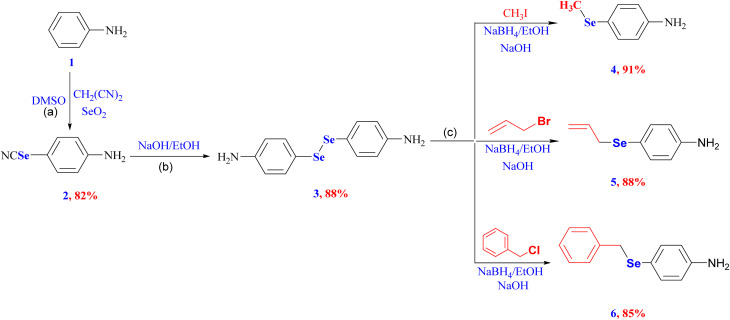
Synthesis of selenium-functionalized amines 4–6. Reagents: (a) Aniline (1) (10 mmol), SeO_2_ (12 mmol), malononitrile (6 mmol), DMSO (30 mL); (b) selenocyanatobenzenamine (2) (1 mmol), NaOH (3 mmol), EtOH (40 mL); (c) *Bis*(4-aminophenyl)diselenide (3) (3 mmol), alkyl halides (CH_3_I, CH_2_=CHCH_2_Br, or PhCH_2_Cl) (6 mmol), EtOH (50 mL), NaBH_4_ (18 mmol), NaOH (6 mmol).

**Scheme 2:**
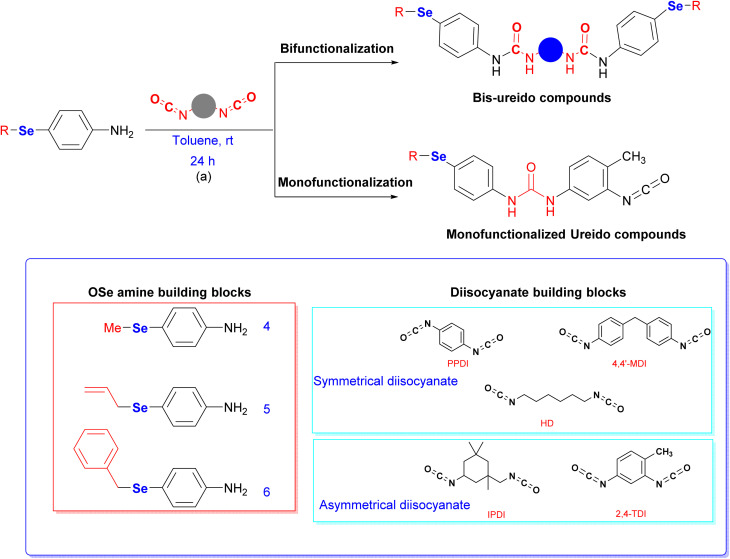
Synthesis of selenium-functionalized ureido architectures *via* coupling with diisocyanates. Reagents: (a) organoselenium amines (10 mmol), toluene (10 mL), diisocyanate (20 mmol, rt for 24 h).

**Scheme 3:**
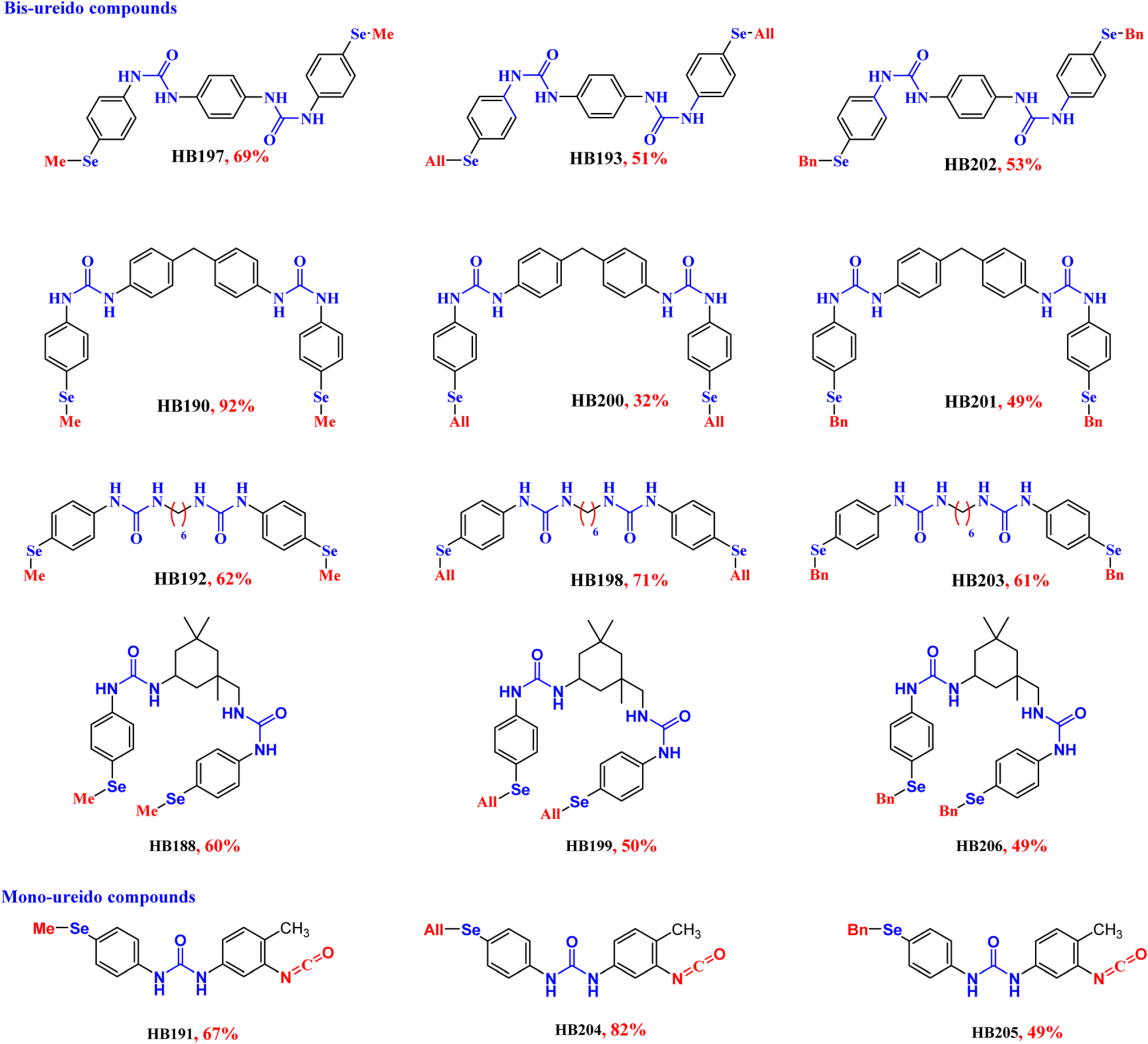
Selenium-functionalized ureido architecture.

The synthetic approach for obtaining the desired OSe-based ureido compounds is illustrated in Schemes 1–3. The primary aim of this research is to develop innovative ureido architectures that integrate the Se redox center, thus leveraging the overall biological and pharmacological profiles of the resulting compounds.

The OSe-functionalized anilines 4, 5, and 6 were successfully synthesized in good yields (up to 91%) from 4,4′-diselanediyldianiline (3) by reduction using NaBH_4_ and subsequent reaction with appropriate alkyl halides (Scheme 1).^53,54^ The incorporation of the RSe functionality at the *para*-position in relation to the NH_2_ group enables an optimal steric and electronic balance, enhancing the nucleophilicity of the aniline NH_2_ group while providing a Se redox-active center.^47,55^

With the prerequisite OSe-functionalized anilines 4, 5, and 6 in hand, we proceeded to establish the ureido linkages through the nucleophilic addition of the primary amino groups to various diisocyanates, including symmetrical diisocyanates (*e.g.*, 1,4-phenylene diisocyanate (PPDI), 4,4′-methylene diphenyl diisocyanate (4,4′-MDI), hexamethylene diisocyanate (HDI)) and asymmetrical diisocyanates (*e.g.*, isophorone diisocyanate (IPDI) and 2,4-toluene diisocyanate (2,4-TDI)) to afford the corresponding ureido derivatives (Scheme 2).

The reaction of OSe amines 4, 5, and 6 with PPDI, 4,4′-MDI, HDI, and IPDI diisocyanates proceeded smoothly through the nucleophilic attack of the NH_2_ on the carbon of the isocyanate, which is very electrophilic (32–92%) (Scheme 3).

On the other hand, the reaction with the asymmetrical diisocyanate 2,4-TDI provided only the *mono*-functionalized ureido derivatives HB191, HB204, and HB205 in good yields (up to 82%) rather than the *bis*-ureido adducts. This behavior can be explained by the inherent asymmetry and different reactivity of the two isocyanate groups. The observed regioselectivity arises from the preferential reactivity of the two isocyanate groups in 2,4-TDI.^56,57^ The *ortho*-isocyanate group is highly deactivated because of the steric hindrance by the adjacent methyl substituent. On the other hand, the *para*-isocyanate group is sterically unhindered and significantly more reactive.^58,59^ Accordingly, nucleophilic addition occurs exclusively at the *para* position, affording the *mono*-ureido regioisomers HB191, HB204, and HB205. Furthermore, the second functionalization, *i.e.*, formation of the *bis*-ureido derivative, might also be unfavored owing to the deactivation of the *ortho*-isocyanate group after the formation of the *mono*-ureido regioisomer.

The FT-IR spectra provided the most definitive confirmation of successful urea bond formation across all compounds. In the case of the *bis*-ureido products, broad N–H signals were observed in the 3279–3363 cm^−1^ region, characteristic of urea N–H groups. Furthermore, the C=O urea functionality stretch appeared in the range 1624–1686 cm^−1^. In the case of the IPDI-derived compounds HB188 and HB199, two carbonyl bands were detected at 1686 cm^−1^ and 1644–1645 cm^−1^, respectively, confirming the two non-equivalent urea C=O groups present in the asymmetric isophorone skeleton.

Moreover, the most significant spectral distinction between the *bis*- and *mono*-ureido derivatives was confirmed by the residual isocyanate (–NCO) stretching band in the spectra of the 2,4-TDI-derived *mono*-ureido adducts. Within this context, compounds HB191, HB204, and HB205 showed sharp, strong absorptions at 2278, 2278, and 2269 cm^−1^, respectively, in a region absent from the spectra of the *bis*-ureido compounds, where both isocyanate groups had been completely reacted.

In the case of ^1^H-NMR spectra, the most important feature was the entire absence of the starting NH_2_ signals of the OSe-based amines and the concurrent appearance of the urea N–H protons. In this context, the aryl-NH appeared at *δ* 8.25–8.44 ppm, while the aliphatic-NH resonated at a significantly higher field (*δ* 5.91–6.27 ppm). The *mono*-ureido 2,4-TDI compounds HB191, HB204, and HB205 showed only one urea N–H signal per nitrogen (*δ* 9.14–8.51), confirming selective *mono*-functionalization. On the other hand, the urea C=O carbon was the main structural marker in the ^13^C-NMR spectra, appearing at *δ* 152.74–155.72 ppm.

Notably, the HPLC chromatograms show single, well-resolved peaks for the target compounds, indicating that the synthesized compounds possess a purity greater than 99%, and therefore no additional significant impurities were detected that would require further toxicological characterization.

### 2.2. Biological evaluation

#### 2.2.1. The percentage of cellular inhibition of growth (GI) against various normal and tumor cell lines

Eight human cancer cell lines were used, human squamous cell carcinoma (HN9), hepatocellular carcinoma (HuH7, HEPG2), human hypopharyngeal squamous cell carcinoma (FaDu), human breast cancer (MCF7), human non-small lung cancer (A549), human colorectal carcinomas (HCT_116_), and human melanoma (A375)—were used to test the growth inhibition (GI) efficacy of diisocyanate-derived OSe urea derivatives. Furthermore, two normal cell lines: human skin fibroblasts (HSF) and the human oral epithelial cells (OEC) were used to measure the GI% of the new urea-linked OSe compounds. The GI activity data for the substances under evaluation are shown in Table 1, alongside those for the reference anticancer drug, doxorubicin.

**Table 1:** The growth inhibition percentages of 100 µg mL^−1^ of the new organoselenium compounds linked to urea against both tumor and normal cell lines

Cell line/Comp. code	HN9	HuH7	FaDu	MCF7	HEPG2	A549	HCT_116_	A375	Mean	OEC	HSF
HB188	74.05	88.58	77.27	67.74	76.23	74.38	85.36	66.85	76.31	32.70	53.59
HB190	9.38	2.42	22.48	7.64	31.12	15.07	9.63	10.53	13.19	7.33	9.76
HB191	33.99	87.15	76.20	61.97	35.92	59.94	81.67	81.02	64.73	45.43	37.99
HB192	44.14	41.32	30.58	21.34	42.60	27.62	42.02	41.07	36.34	11.96	8.44
HB193	31.89	47.13	13.79	33.40	31.38	20.09	60.51	41.47	34.96	54.07	1.63
HB197	42.40	49.43	38.27	32.40	39.40	20.62	59.68	50.78	41.62	5.096	15.94
HB198	77.17	55.32	21.85	29.38	42.04	37.94	61.42	43.66	46.10	54.67	40.51
HB199	85.83	59.92	53.49	38.71	56.65	37.23	62.89	15.37	51.26	64.65	37.18
HB200	63.94	54.89	21.08	38.36	35.07	27.67	48.86	44.20	41.76	68.12	12.25
HB201	50.11	50.36	41.22	34.43	18.16	35.49	54.74	35.77	40.04	36.38	19.85
HB202	26.62	44.69	19.28	29.73	35.43	11.16	49.23	38.65	31.85	23.51	10.70
HB203	63.39	57.06	45.90	39.31	57.34	35.86	57.14	48.17	50.52	30.93	6.38
HB204	82.15	80.54	71.96	70.73	83.21	75.97	77.50	79.70	77.72	52.83	39.75
HB205	47.19	53.20	19.75	31.78	56.57	12.36	60.68	48.78	41.29	60.29	3.913
HB206	68.24	59.41	51.73	46.26	51.87	39.25	56.34	49.74	52.86	57.83	27.29
DOX	51.73	67.99	79.34	58.65	55.62	39.14	64.36	78.26	61.89	31.96	29.57
Average	53.26	56.04	42.76	40.11	46.79	35.61	58.25	48.38		39.86	22.17

##### 2.2.1.1 Structure activity relationships (SAR)

**Fig. 3:**
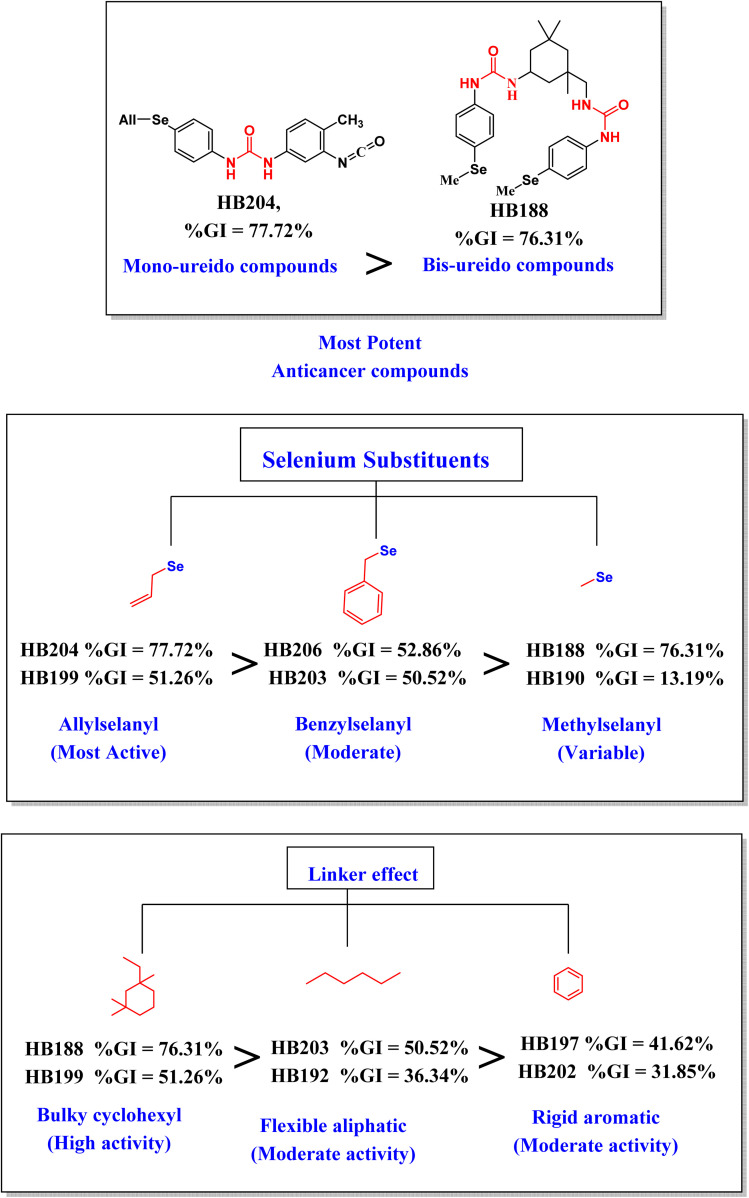
Novel compounds' structure–activity relationship.

The antiproliferative activity of the synthesized Se-containing urea derivatives was systematically analyzed to elucidate the relationship between structural features and biological performance. The observed activity profile is primarily influenced by three factors: (i) the degree of ureido substitution (*mono-vs. bis*-ureido), (ii) the kind of Se substituent, and (iii) the shape and flexibility of the linker that connects the pharmacophoric units (Fig. 3).

*Mono*-ureido and *bis*-ureido derivatives were clearly different from each other. The *mono*-ureido compound HB204 had the highest average growth inhibition (% GI = 77.72), which was much higher than the reference drug doxorubicin (% GI = 61.89). Other *mono*-ureido derivatives, including HB191 (% GI = 64.73) and HB205 (% GI = 41.29), also demonstrated moderate-to-high activity. Most *bis*-ureido compounds, in contrast, exhibited moderate antiproliferative effects, typically within the % GI range of 30–55. The only one that was different was HB188, which had a high average % GI of 76.31. These findings indicate that *mono*-ureido scaffolds are typically more beneficial, likely due to their reduced steric hindrance and enhanced conformational flexibility.

The nature of the Se substituent is essential for changing activity. Compounds with the allylselanyl (prop-1-en-1-ylselanyl) group, like HB204 (% GI = 77.72), HB199 (% GI = 51.26), HB198 (% GI = 46.10), and HB200 (% GI = 41.76), showed good to moderate activity, which shows how useful unsaturated selenium substituents can be. Benzylselanyl derivatives (HB206, % GI = 52.86; HB203, % GI = 50.52; HB201, % GI = 40.04; and HB202, % GI = 31.85) had moderate activity, which means that more hydrophobic bulk is satisfactory but not perfect. Methylselenyl derivatives, on the other hand, usually had less or less consistent activity. For example, HB190 (% GI = 13.19), HB192 (% GI = 36.34), and HB197 (% GI = 41.62) all showed this, but HB188 (% GI = 76.31) is an exceptional case.

The effect of linker architecture was also obvious. Compounds with bulky hydrophobic linkers, such as the trimethylcyclohexyl moiety in HB188 (% GI = 76.31), HB199 (% GI = 51.26), and HB206 (% GI = 52.86), were more active. This shows that biological activity depends a lot on hydrophobic core structures. However, derivatives with flexible aliphatic linkers (HB192, % GI = 36.34; HB200, % GI = 41.76; and HB203, % GI = 50.52) and solid aromatic linkers (HB197, % GI = 41.62; and HB202, % GI = 31.85) usually had moderate activity regardless of whether they were combined with very favorable substituents, like in HB204.

The most active compounds, particularly HB204 and HB188, displayed notable selectivity for normal cell lines, thereby enhancing their potential as effective anticancer agents. A preliminary selectivity assessment based on single-dose GI% screening indicated that HB204 and HB188 exhibited preferential antiproliferative activity toward cancer cells relative to normal cells, with preliminary selectivity ratios of 1.68 and 1.77, respectively.

These findings collectively suggest that optimal antiproliferative activity in this series is achieved through a synergistic combination of structural features, rather than a singular determinant. *Mono*-ureido frameworks, allylselanyl substitution, and well-balanced hydrophobic linkers are all important parts of improving activity. HB204 stands out as the most promising lead candidate among all compounds, as it combines these good traits and has the highest average % GI across the tested panel.

#### 2.2.2. *In vitro* cytotoxicity evaluation

**Fig. 4:**
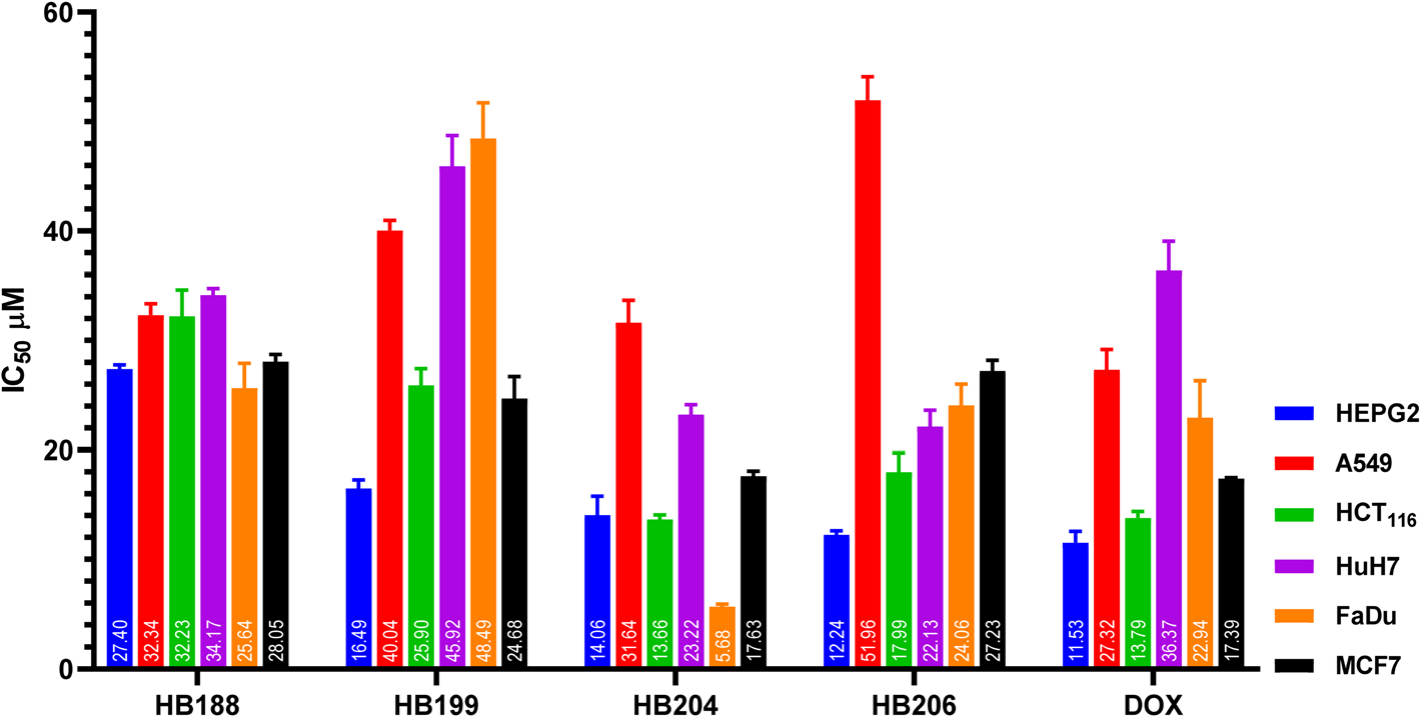
The half maximal cytotoxic inhibitory concentration (IC_50_) of the compounds HB188, HB199, HB204, and HB206 toward HEPG2, A549, HCT_116_, HuH7, FaDu, and MCF7 cancer cell lines using the sulforhodamine B (SRB) assay. Graphs and data analysis were performed using GraphPad InStat, version 8.02. The results are expressed as the mean ± SD of 3 separate experiments performed in 3 replicates.

Cytotoxic effects (IC_50_ values) of the examined candidates were assessed against tumor cell lines with the highest GI percentage. The standard SRB analysis was used on the following cell lines: HuH7, HEPG2, FaDu, MCF7, A549, and HCT_116_ (ref. 60) (Fig. 4).

The investigated compounds were evaluated at different concentrations (6.25, 12.5, 25, and 50 mg mL^−1^) on the chosen cancer cell lines. Among them, HB204 demonstrated the most potent activity, with the lowest IC_50_ values recorded as 14.06, 31.64, 13.66, 23.22, 5.68, and 17.63 µM toward HEPG2, A549, HCT_116_, HuH7, FaDu, and MCF7, respectively. The reference drug doxorubicin had IC_50_ values of 11.53, 27.32, 13.79, 36.37, 22.94, and 17.39 µM against the same cell lines in the same order. It is important to note that HB204 exhibited greater cytotoxicity against the FaDu cell line than doxorubicin, indicating that it has strong potential as an anticancer drug (Fig. 4).

#### 2.2.3. Apoptosis-related protein expression

To investigate the apoptotic mechanism of the highly active compound, HB204, its impact on protein expression in the highly sensitive FaDu cancer cell line was examined. This investigation aimed to detect any changes in the expression of key proteins involved in apoptosis and to learn more about the molecular pathways that are affected by HB204.

**Fig. 5:**
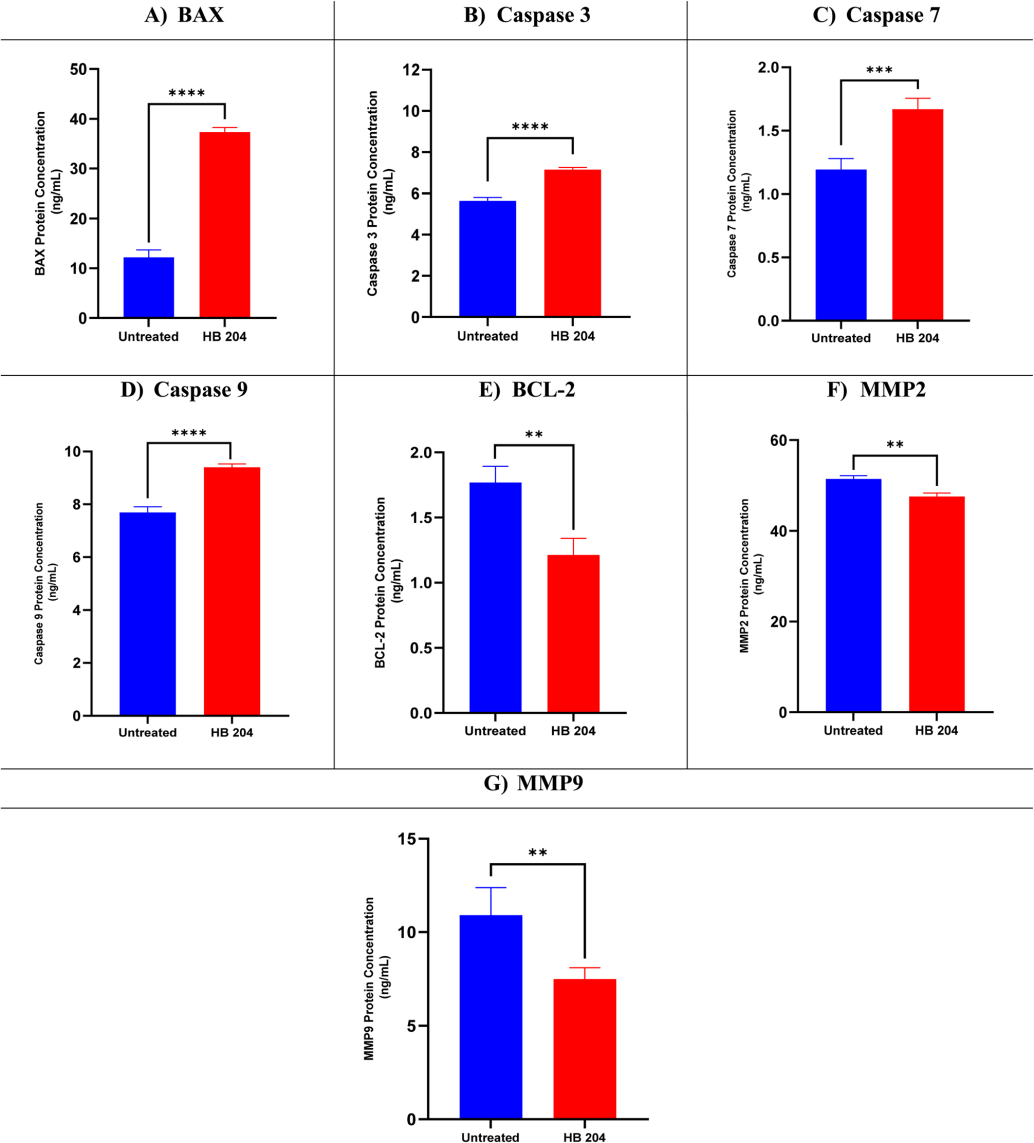
Compound HB204 protein expression levels for BAX (A), caspases 3 (B), 7 (C), and 9 (D), BCL-2 (E), MMP2 (F), and MMP9 (G) in the treated and untreated FaDu cancer cell line. The results are the mean ± SD of 3 separate experiments performed in duplicate. Statistical significance of results was analyzed using one-way ANOVA followed by Tukey's multiple comparison test. * Significantly different from control (*P* < 0.05).

The study evaluated the protein expression concentration of pro-apoptotic proteins (BAX, caspases 3, 7, and 9) and anti-apoptotic proteins (MMP2, MMP9, and BCL-2) in HB204-treated cells *versus* untreated control cells. Notably, treatment with HB204 led to a significant upregulation of the pro-apoptotic markers: BAX (3.06-fold), caspase-3 (1.27-fold), caspase-7 (1.40-fold), and caspase-9 (1.22-fold). In contrast, anti-apoptotic protein expression was downregulated: MMP2 (1.08-fold), MMP9 (1.42-fold), and BCL-2 (1.47-fold). These results further confirm the pro-apoptotic effect of HB204 as shown in Fig. 5.

#### 2.2.4. Cell cycle arrest determination

Given the strong anticancer activity of HB204 toward the FaDu cell line (IC_50_ = 5.68 µM), flow cytometry was used to investigate more closely how it affected the cell cycle to determine the stage at which it exerts its arrest. Interestingly, the experiment was conducted on the untreated FaDu cell line^61–63^ for comparison.

**Fig. 6:**
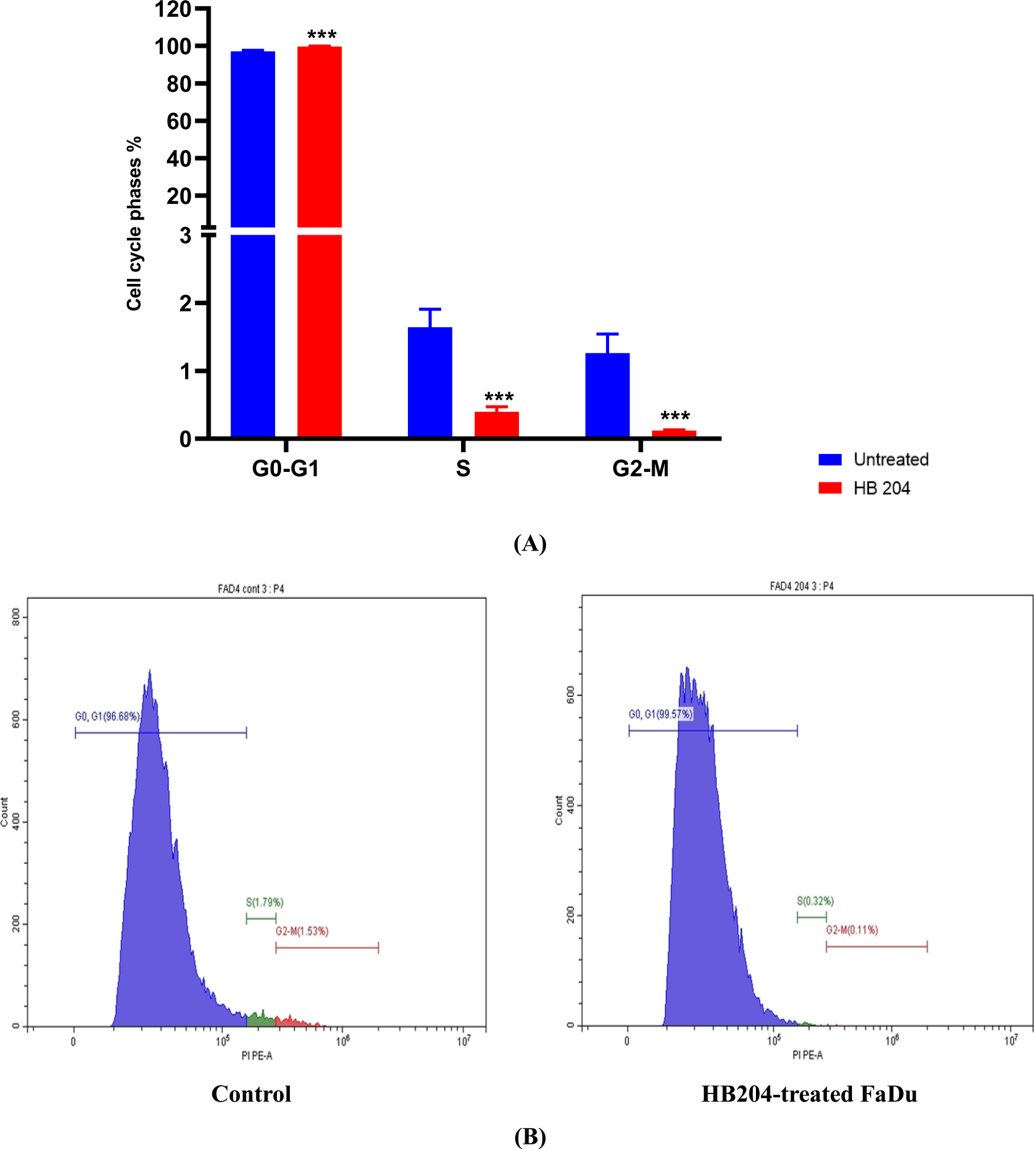
(A) Cell cycle study of treated and untreated FaDu cells using compound HB204; (B) compound HB204-treated FaDu histogram *vs.* control values. Cell cycle phase distribution was analyzed after 48 h exposure to Baricitinib by staining with Propidium iodide (PI). Data are presented as mean ± SD from 3 independent experiments and analyzed using one-way analysis of variance (ANOVA), followed by the Tukey–Kramer multiple comparison test. ^*^Significantly different from the corresponding control cell cycle phases at *P* < 0.05.

According to the flow cytometry results, HB204 treatment caused a notable increase in the pre-G1 cell population, rising from 96.68% in untreated cells to 99.57%. The percentage of cells in the S phase dropped from 1.79% to 0.32%, and the percentage of cells in the G2/M phase dropped from 1.53% to 0.11%. These results indicate that HB204 probably stops cancer from spreading by stopping the cell cycle at the pre-G1 phase, which is one of the ways it fights cancer (Fig. 6).

To confirm the results, the cell cycle analysis was repeated three times (SI).

#### 2.2.5. Apoptosis determination by annexin V-FITC/PI assay

To further investigate the mechanism underlying the potent anticancer activity of HB204, Annexin V-FITC/PI staining followed by flow cytometric analysis was performed using the highly sensitive FaDu cell line. FaDu cells were treated with HB204 and subsequently analyzed to evaluate its ability to induce programmed cell death.

**Fig. 7:**
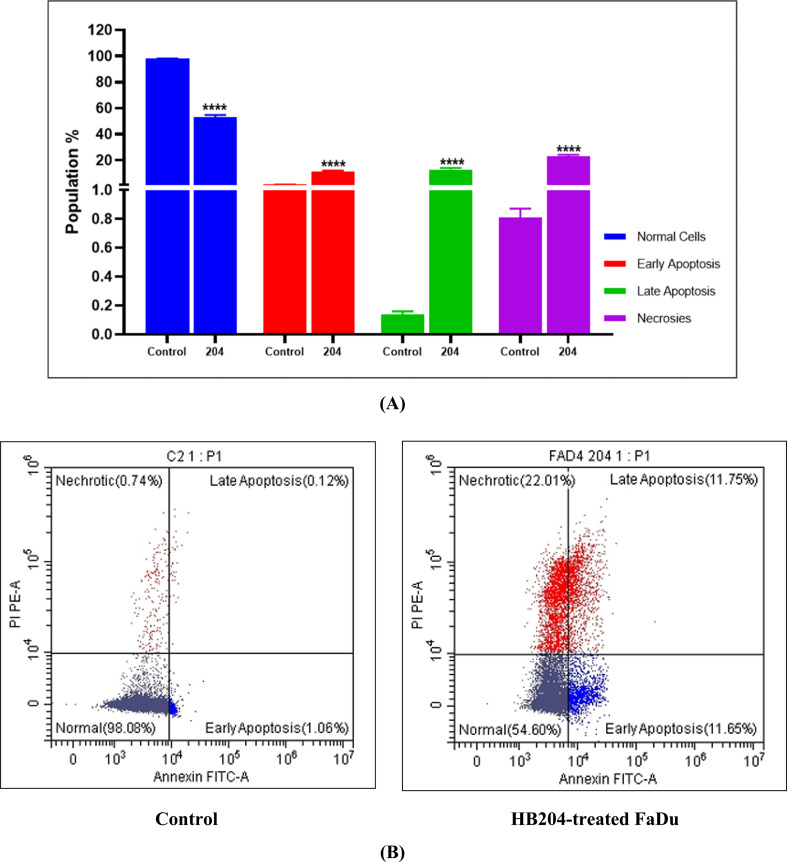
(A) Apoptosis study of treated and untreated FaDu cells using compound HB204; (B) representative Annexin V-FITC/PI quadrant plots of HB204-treated FaDu cells *versus* control values. Apoptosis distribution was analyzed after 48 h exposure to HB204 by staining with Annexin V-FITC and Propidium iodide (PI). Data are presented as the mean ± SD from 3 independent experiments and analyzed using two-way analysis of variance (ANOVA), followed by Sidak's multiple comparisons test. ^*^Significantly different from the corresponding control apoptotic populations at *P* < 0.05.

As depicted in Fig. 7, HB204 markedly increased the apoptotic cell population compared with untreated control cells. The percentage of early apoptotic cells increased from 1.06% in untreated cells to 11.65% following HB204 treatment. Similarly, late apoptotic cells increased from 0.12% to 11.75%. By summing both early and late apoptotic populations, the total apoptosis percentage reached 23.40% in HB204-treated cells compared with only 1.18% in untreated cells, representing approximately a 20-fold increase in apoptosis induction.

Furthermore, HB204 treatment reduced the percentage of viable cells from 98.08% to 54.60%, while the necrotic cell population increased from 0.74% to 22.01%. These findings clearly demonstrate that HB204 exerts its antiproliferative activity through apoptosis induction. Moreover, the obtained results are in good agreement with the observed upregulation of the pro-apoptotic proteins BAX and caspases 3, 7, and 9, together with the downregulation of the anti-apoptotic proteins BCL-2, MMP2, and MMP9, further confirming apoptosis-mediated cell death as a principal mechanism underlying the anticancer activity of HB204.

To confirm the results, the apoptosis analysis was repeated three times (SI).

### 2.3. *In silico* studies

#### 2.3.1. Molecular docking

The superior lead HB204 was subjected to a molecular docking study to investigate its inhibitory potential towards one of the most important apoptotic markers (BAX). The selection of BAX was based on its superior sensitivity to the lead analog (HB204), as indicated by the aforementioned *in vitro* protein expression assay. Moreover, the reported BAX small allosteric inhibitor (BAI 1)^64^ was inserted with the lead HB204 for comparison in terms of binding mode and score.

Analogue HB204 achieved a superior binding score (−5.66 kcal mol^−1^) at a Root Mean Square Deviation (RMSD) value of 1.56 Å. However, the reported BAX inhibitor (BAI 1) displayed a binding score of −5.14 kcal mol^−1^ (RMSD = 1.75 Å).

**Fig. 8:**
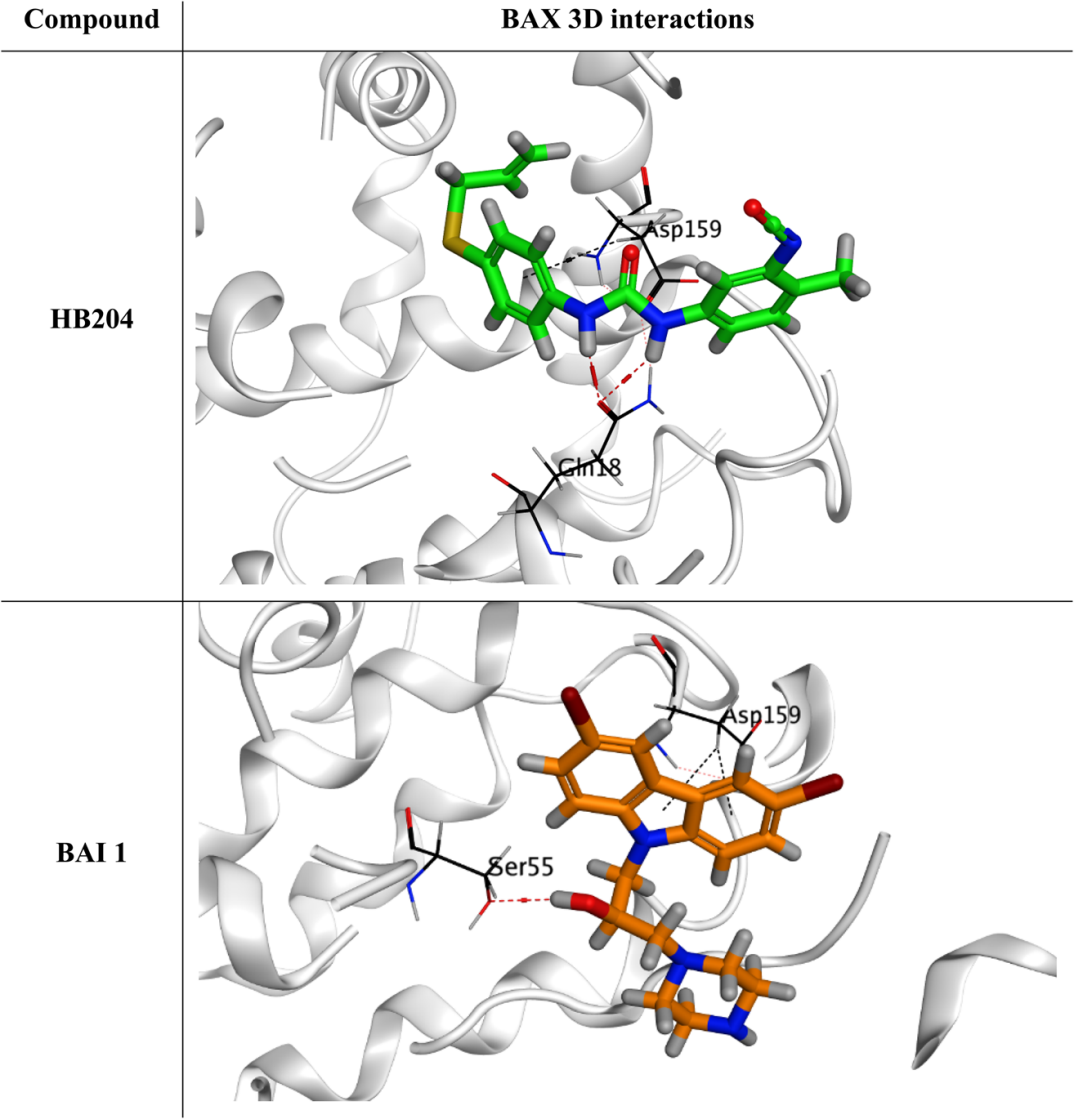
3D Binding interactions of analog (HB204) compared to the reported BAX inhibitor (BAI 1) towards the BAX protein (PDB ID: 1F16).

Analyzing the binding interactions (Fig. 8), analog HB204 showed the formation of two hydrogen bonds (through the urea moiety) with Gln18 and one pi–hydrogen bond with Asp159 (*via* its phenyl ring attached to the selenium atom). On the other hand, the reported BAX inhibitor (BAI 1) formed one hydrogen bond with Ser55 (*via* its –OH group) and two pi-hydrogen bonds with Asp159 (*via* its tricyclic moiety).

Based on the above, we could explain the strong *in vitro* BAX-inhibitory potential of analog HB204 (described earlier) and clarify its recommended binding interactions as well.

#### 2.3.2. Molecular dynamics (MD) simulation

The outstanding lead HB204 and the reported BAX small allosteric inhibitor (BAI 1)^64^ were subject to a 500 ns MD simulation to confirm the results of molecular docking. The protein RMSD (P-RMSD) and the ligand RMSD (L-RMSD) were recorded to investigate the stability of the ligand-BAX complexes. The movements of the BAX backbone (C*α*) relative to its initial position and the ligand as a function of time were evaluated.

**Fig. 9:**
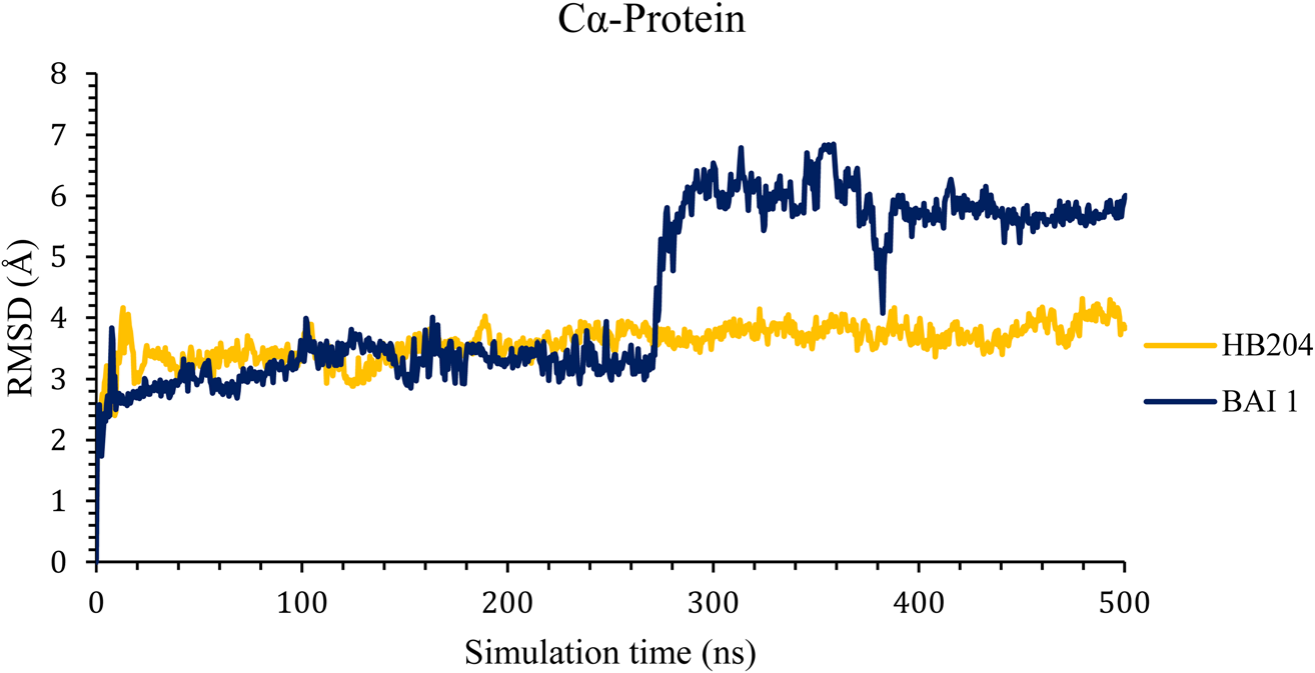
Root mean square deviations (P-RMSDs) of the BAX proteins' backbone (PDB ID: 1F16) complexed to HB204 and BAI 1.

Obviously, the HB204 complex showed a more stable P-RMSD (less than 4.00 Å) during the simulation time, comparable to that of the BAI 1 complex. The BAI 1 complex showed an initial stable P-RMSD (less than 4.00 Å) until 275 ns of the simulation time; then it rose to 6.5 Å with unstable behaviour (Fig. 9).

**Fig. 10:**
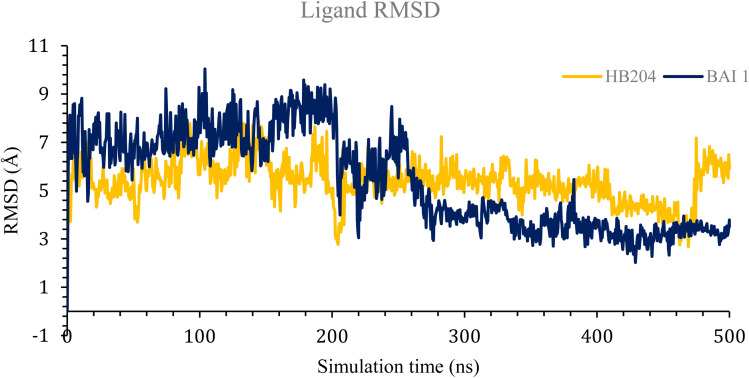
Root mean square deviations (L-RMSDs) of ligands to proteins (PDB ID: 1F16) of HB204 and BAI 1.

The L-RMSDs (Fig. 10) clarified that HB204 showed better stability inside the BAX active site, moving by 6.00 Å on average (between 4 and 7.5 Å). However, BAI 1 showed lower stability, especially in the first half of the simulation time (reaching up to 10 Å). Then, its RMSD decreased to 4 Å at the end of the simulation time.

**Fig. 11:**
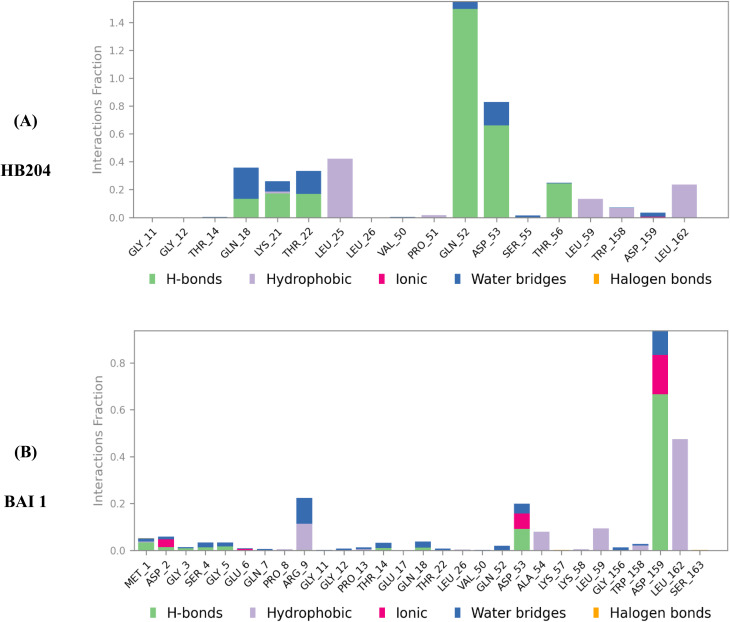
Protein-ligand contacts of HB204 (A) and BAI 1 (B) toward the active site of the BAX protein (PDB ID: 1F16).

Moreover, the ligand-amino acid interactions analysis was carried out. HB204 and BAI 1 interactions within the BAX active site are depicted in Fig. 11. The histogram of HB204 (Fig. 11A) showed that Gln52 is the most crucial amino acid contributing to the interactions (150%) through hydrogen bonds (145%) and water-bridged hydrogen bonds (5%). Followed by Asp53 (80%) through hydrogen bonds (70%) and water-bridged hydrogen bonds (10%). On the other hand, the histogram of BAI 1 (Fig. 11B) showed that Asp159 is the highest amino acid in the interactions (100%) through hydrogen bonds (70%), ionic bonds (20%), and water-bridged hydrogen bonds (10%). Followed by Leu162 (45%) through hydrophobic interactions only.

## 3. Conclusion

To sum up, a new set of urea-linked OSe hybrids was successfully designed, synthesized, and biologically evaluated as potential anticancer agents. The molecular hybridization strategy successfully integrated the redox-modulating characteristics of selenium with the binding properties of the urea pharmacophore, yielding compounds with considerable antiproliferative efficacy. Biological screening demonstrated that activity is strongly influenced by structural features, particularly the degree of ureido substitution, the nature of the Se substituent, and linker architecture. *Mono*-ureido derivatives typically demonstrated superior performance relative to *bis*-ureido analogs, with allylselanyl substitution and hydrophobic linkers enhancing activity. Among all the synthesized compounds, HB204 was the most promising. It had the greatest average % GI, the best selectivity, and the best cytotoxicity when compared to doxorubicin in certain cancer cell lines. Mechanistic studies confirmed that HB204 triggers apoptosis and cell cycle arrest by altering critical apoptotic proteins. Furthermore, Annexin V-FITC/PI flow cytometric analysis demonstrated a marked increase in total apoptotic cells from 1.18% in untreated FaDu cells to 23.40% following HB204 treatment, confirming apoptosis-mediated cell death as a major contributor to its anticancer activity. Docking studies showed that HB204 binds strongly to BAX, with a higher affinity than BAI 1 and important interactions with residues. Furthermore, the MD simulation for 500 ns confirmed the results of molecular docking. This supports its proposed mechanism and shows that it could be optimized even further. Overall, our analysis identifies HB204 as a viable lead compound for additional optimization and preclinical research in head and neck cancer therapy and offers insightful information about the SAR of OSe-based urea hybrids. Although promising anticancer activity was observed, broader toxicological characterization beyond cytotoxicity remains necessary at the early drug-development stage to confirm the safety profile of the synthesized compounds.

## 4. Materials and methods

### 4.1. Chemistry

Diisocyanates PPDI, 4,4′-MDI, HDI, IPDI, and 2,4-TDI were purchased from Sigma-Aldrich. OSe amines 3, 4, and 5 were synthesized following our reported method (experimental details are also listed in the SI).^65–68^ Refer to SI for IR, ^1^H-NMR, ^13^C-NMR, MS, and HPLC copies of the recently developed compounds.

Regarding nitrosamine risk, no nitrosating reagents (*e.g.*, nitrite salts or nitrous acid) were employed in any step, and the reactions were conducted under conditions not conducive to nitrosation. In addition, the synthesized compounds do not possess nitrosatable secondary or tertiary amine moieties. Therefore, the formation of nitrosamine impurities is not expected under the conditions described.

#### 4.1.1. General procedure for the synthesis of selenium-based ureido derivatives

To the OSe amines (10 mmol) in toluene (10 mL), the appropriate isocyanate (20 mmol) was added, and the solution was stirred for 24 h. The solid formed was isolated by filtration, washed with boiling toluene, and dried.

##### 4.1.1.1 The synthesis of 1,1'-(1,4-phenylene)bis(3-(4-(methylselanyl)phenyl)urea) (HB197)

Compound HB197 was synthesized from OSe amine 4 (20 mmol) and PPDI (10 mmol) in toluene (10 mL) and separated as white powder; yield = 69%; MP = > 300 °C; ^1^H-NMR (500 MHz, DMSO-*d*_6_) *δ* 8.60 (s, 2H, 2NH), 8.49 (s, 2H, 2NH), 7.35 (d, *J* = 16.5 Hz, 12H, Ar–H), 2.29 (s, 6H, 2CH_3_); ^13^C-NMR (125 MHz, DMSO-*d*_6_) *δ* 152.98, 138.89, 131.60, 123.09, 121.23, 119.45, 114.55, 7.82; MS (ESI): *m*/*z* = calcd. For C_22_H_22_N_4_O_2_Se_2_ [M^+^]: 532.3, found 532.8 [M^+^], 564.7 [M^+^ + Na + MeOH].

##### 4.1.1.2 The synthesis of 1,1'-(1,4-phenylene)bis(3-(4-(allylselanyl)phenyl)urea) (HB193)

Compound HB193 was synthesized from OSe amine 5 (20 mmol) and PPDI (10 mmol) in toluene (10 mL) and separated as white powder; yield = 51%; MP = 234–235 °C; ^1^H-NMR (500 MHz, DMSO-*d*_6_) *δ* 8.64 (s, 2H, 2NH), 8.54 (s, 2H, 2NH), 7.38 (s, 7H, Ar–H), 7.35 (d, *J* = 6.2 Hz, 5H, Ar–H), 5.95–5.79 (m, 2H, 2CH=), 4.96–4.84 (m, 4H, 2CH_2_=), 3.50 (t, *J* = 6.6 Hz, 4H, 2SeCH_2_); ^13^C-NMR (125 MHz, DMSO-*d*_6_) *δ* 152.94, 139.73, 135.24, 134.48, 121.05, 119.51, 119.35, 119.18, 117.11, 30.78; MS (ESI): *m*/*z* = calcd. For C_26_H_26_N_4_O_2_Se_2_ [M^+^]: 584.4, found 584.8 [M^+^], 608.9 [M^+^ + Na–H].

##### 4.1.1.3 The synthesis of 1,1'-(1,4-phenylene)bis(3-(4-(benzylselanyl)phenyl)urea) (HB202)

Compound HB202 was synthesized from OSe amine 6 (20 mmol) and PPDI (10 mmol) in toluene (10 mL) and separated as white powder; yield = 53%; MP = MP = > 300 °C; FT-IR (*ν*, cm^−1^): 3297, 3024, 1639, 1583, 1543, 1509, 1486, 1450, 1229, 1174, 1062, 1010, 807, 692, 623, 692, 497; FT-IR (*ν*, cm^−1^): 3297, 3024, 1639, 1583, 1543, 1509, 1486, 1450, 1229, 1174, 1062, 1010, 807, 692, 623, 692, 497; ^1^H-NMR (400 MHz, DMSO-*d*_6_) *δ* 8.63 (s, 1H, 2NH), 8.51 (s, 1H, 2NH), 7.31 (t, *J* = 4.8 Hz, 12H, Ar–H), 7.22–7.13 (m, 10H, Ar–H), 4.11 (s, 4H, 2SeCH_2_); ^13^C-NMR (100 MHz, DMSO-*d*_6_) *δ* 152.89, 139.77, 139.46, 134.39, 129.15, 128.66, 127.04, 121.53, 121.03, 119.47, 119.09, 31.91; MS (ESI): *m*/*z* = calcd. For C_34_H_30_N_4_O_2_Se_2_ [M^+^]: 684.5, found 684.8 [M^+^], 720.5 [M^+^ + K–3H].

##### 4.1.1.4 The synthesis of 1,1'-(methylenebis(4,1-phenylene))bis(3-(4-(methylselanyl)phenyl)urea) (HB190)

Compound HB190 was synthesized from OSe amine 4 (20 mmol) and 4,4′-MDI (10 mmol) in toluene (10 mL) and separated as white powder; yield = 92%; MP = MP = > 300 °C; FT-IR (*ν*, cm^−1^): 3287, 3027, 2995, 1637, 1582, 1579, 1543, 1489, 1389, 1231, 1174, 900, 806, 760; ^1^H-NMR (400 MHz, DMSO-*d*_6_) *δ* 8.59 (s, 2H, 2NH), 8.53 (s, 2H, 2NH), 7.38–7.28 (m, 12H, Ar–H), 7.08 (d, *J* = 8.5 Hz, 4H, Ar–H), 3.77 (s, 2H, CH_2_), 2.28 (s, 6H, 2CH_3_); ^13^C-NMR (100 MHz, DMSO-*d*_6_) *δ* 152.88, 138.77, 137.93, 135.50, 131.57, 129.32, 123.17, 119.46, 118.86, 40.27, 7.80; MS (ESI): *m*/*z* = calcd. For C_29_H_28_N_4_O_2_Se_2_ [M^+^]: 622.4, found 622.8 [M^+^], 646.8 [M^+^ + Na–H].

##### 4.1.1.5 The synthesis of 1,1'-(methylenebis(4,1-phenylene))bis(3-(4-(allylselanyl)phenyl)urea) (HB200)

Compound HB200 was synthesized from OSe amine 5 (20 mmol) and 4,4′-MDI (10 mmol) in toluene (10 mL) and separated as white powder; yield = 32%; MP = MP = > 300 °C; FT-IR (*ν*, cm^−1^): 3296, 2976, 2899, 1639, 1585, 1541, 1507, 1489, 1426, 1408, 1303, 1232, 806, 624, 499, 510; ^1^H-NMR (600 MHz, DMSO-*d*_6_) *δ* 8.65 (s, 2H, 2NH), 8.57 (s, 2H, 2NH), 7.38 (s, 8H, Ar–H), 7.34 (d, *J* = 8.5 Hz, 4H, Ar–H), 7.10 (d, *J* = 8.4 Hz, 4H, Ar–H), 5.92–5.82 (m, 2H, 2CH=), 4.96–4.84 (m, 4H, 2CH_2_=), 3.78 (d, *J* = 15.4 Hz, 2H, CH_2_), 3.49 (t, *J* = 6.7 Hz, 4H, 2SeCH_2_); ^13^C-NMR (150 MHz, DMSO-*d*_6_) *δ* 152.86, 139.66, 137.92, 135.56, 135.23, 134.47, 129.35, 121.16, 119.22, 118.92, 117.10, 39.60, 30.78; MS (ESI): *m*/*z* = calcd. For C_33_H_32_N_4_O_2_Se_2_ [M^+^]: 674.5, found 674.6 [M^+^], 698.5 [M^+^ + Na–H].

##### 4.1.1.6 The synthesis of 1,1'-(methylenebis(4,1-phenylene))bis(3-(4-(benzylselanyl)phenyl)urea) (HB201)

Compound HB201 was synthesized from OSe amine 6 (20 mmol) and 4,4′-MDI (10 mmol) in toluene (10 mL) and separated as white powder; yield = 49%; MP = MP = > 300 °C; ^1^H-NMR (600 MHz, DMSO-d_6_) *δ* 8.67 (s, 2H, NH), 8.58 (s, 2H, NH), 7.37–7.32 (m, 12H, Ar–H), 7.21 (dt, *J* = 15.4, 7.1 Hz, 8H, Ar–H), 7.17–7.14 (m, 2H, Ar–H), 7.09 (dd, *J* = 8.8, 5.8 Hz, 4H, Ar–H), 4.14–4.08 (m, 4H, 2CH_2_), 3.79 (d, *J* = 4.0 Hz, 2H, CH_2_), 3.32 (s, 2H, CH_2_); ^13^C-NMR (150 MHz, DMSO-*d*_6_) *δ* 152.85, 139.72, 139.47, 137.91, 135.57, 134.42, 129.36, 129.17, 128.68, 127.06, 121.67, 119.16, 118.92, 39.60, 31.95; MS (ESI): *m*/*z* = calcd. For C_41_H_36_N_4_O_2_Se_2_ [M^+^]: 774.6, found 774.5 [M^+^], 798.6 [M^+^+Na–H], 810.5 [M^+^ + K–3H].

##### 4.1.1.7 The synthesis of 1,1'-(hexane-1,6-diyl)bis(3-(4-(methylselanyl)phenyl)urea) (HB192)

Compound HB192 was synthesized from OSe amine 4 (20 mmol) and HDI (10 mmol) in toluene (10 mL) and separated as white powder; yield = 62%; MP = 227–228 °C; ^1^H-NMR (500 MHz, DMSO-*d*_6_) *δ* 8.38 (s, 2H, 2NH), 7.29 (dd, *J* = 20.1, 8.7 Hz, 8H, Ar–H), 6.08 (s, 2H, 2NH), 3.30 (s, 4H, 2CH_2_), 3.05 (dd, *J* = 12.7, 6.6 Hz, 4H, 2CH_2_), 2.26 (s, 6H, 2CH_3_), 1.40 (d, *J* = 6.3 Hz, 4H, 2CH_2_), 1.28 (s, 4H, 2CH_2_); ^13^C-NMR (125 MHz, DMSO-*d*_6_) *δ* 155.53, 139.73, 131.71, 122.00, 118.93, 39.44, 30.17, 26.57, 7.93; MS (ESI): *m*/*z* = calcd. For C_22_H_30_N_4_O_2_Se_2_ [M^+^]: 540.4, found 540.9 [M^+^], 564.9 [M^+^ + Na–H].

##### 4.1.1.8 The synthesis of 1,1'-(hexane-1,6-diyl)bis(3-(4-(allylselanyl)phenyl)urea) (HB198)

Compound HB198 was synthesized from OSe amine 5 (20 mmol) and HDI (10 mmol) in toluene (10 mL) and separated as white powder; yield = 71%; MP = 188–189 °C; FT-IR (*ν*, cm^−1^): 3314, 2930, 2856, 2856, 1624, 1578, 1545, 1475, 1487, 1236, 1070, 908, 814, 668, 635, 498; ^1^H-NMR (500 MHz, DMSO-*d*_6_) *δ* 8.41 (s, 2H, 2NH), 7.31 (s, 8H, Ar–H), 6.11 (s, 2H, 2NH), 5.85 (ddt, *J* = 17.5, 9.9, 7.6 Hz, 2H, 2CH=), 4.93–4.82 (m, 4H, 2CH_2_=), 3.50–3.44 (m, 4H, 2SeCH_2_), 3.05 (dd, *J* = 12.8, 6.7 Hz, 4H, CH_2_), 1.47–1.37 (m, 4H, CH_2_), 1.29 (d, *J* = 6.6 Hz, 4H, CH_2_); ^13^C-NMR (125 MHz, DMSO-*d*_6_) *δ* 155.49, 140.55, 135.26, 134.59, 119.99, 118.65, 117.00, 39.44, 30.87, 30.16, 26.56; MS (ESI): *m*/*z* = calcd. For C_26_H_34_N_4_O_2_Se_2_ [M^+^]: 592.5, found 592.9 [M^+^], 616.8 [M^+^ + Na].

##### 4.1.1.9 The synthesis of 1,1'-(hexane-1,6-diyl)bis(3-(4-(benzylselanyl)phenyl)urea) (HB203)

Compound HB203 was separated from OSe amine 6 (20 mmol) and PPDI (10 mmol) in toluene (10 mL) and isolated as white powder; yield = 61%; MP = 200–201 °C; FT-IR (*ν*, cm^−1^): 3298, 2932, 2859, 1628, 1584, 1556, 1522, 1451, 1478, 825, 754, 692, 669, 624, 504; ^1^H-NMR (500 MHz, DMSO-*d*_6_) *δ* 8.44 (s, 2H, 2NH), 7.31 (m, 8H, Ar–H), 7.22 (t, *J* = 7.3 Hz, 4H, Ar–H), 7.16 (dd, *J* = 12.7, 7.0 Hz, 6H, Ar–H), 6.12 (d, *J* = 5.1 Hz, 2H, NH), 4.08 (s, 2H, 4H, 2CH_2_), 3.05 (dd, *J* = 12.4, 6.3 Hz, 4H, 2CH_2_), 1.41 (s, 4H, 2CH_2_), 1.29 (s, 4H, 2CH_2_); ^13^C-NMR (125 MHz, DMSO-*d*_6_) *δ* 155.46, 140.63, 139.55, 134.53, 129.15, 128.65, 127.02, 120.49, 118.59, 39.44, 32.03, 30.16, 26.57; MS (ESI): *m*/*z* = calcd. For C_34_H_38_N_4_O_2_Se_2_ [M^+^]: 692.6, found 692.9 [M^+^], 732.7 [M^+^ + K–2H].

##### 4.1.1.10 The synthesis of 1-(4-(methylselanyl)phenyl)-3-((1,3,3-trimethyl-5-(3-(4-(methylselanyl)phenyl)ureido)cyclohexyl)methyl)urea (HB188)

Compound HB188 was synthesized from OSe amine 4 (20 mmol) and IPDI (10 mmol) in toluene (10 mL) and separated as white powder; yield = 60%; MP = 240–241 °C; FT-IR (*ν*, cm^−1^): 3363, 3299, 2926, 1686, 1644, 1587, 1560, 1541, 1236, 816, 609, 502; ^1^H-NMR (400 MHz, DMSO-*d*_6_) *δ* 8.36 (s, 1H, NH), 8.25 (s, 1H, NH), 7.33–7.20 (m, 8H, Ar–H), 6.17 (s, 1H, NH), 5.91 (s, 1H, NH), 3.87–3.64 (m, 2H, CH_2_), 2.82 (ddd, *J* = 37.4, 13.2, 6.2 Hz, 2H, CH_2_), 2.46 (dt, *J* = 3.6, 1.8 Hz, 1H, CH), 1.55 (t, *J* = 13.8 Hz, 3H, CH_3_), 1.15–1.02 (m, 12H, CH_2_), 1.00 (s, 3H, CH_3_), 0.97 (s, 3H, CH_3_), 0.93–0.80 (m, 6H, 2CH_3_); ^13^C-NMR (100 MHz, DMSO-*d*_6_) *δ* 155.72, 154.78, 139.67, 139.60, 131.75, 131.64, 122.03, 122.01, 118.85, 118.77, 53.21, 47.34, 46.55, 42.86, 42.24, 36.54, 35.40, 32.00, 27.98, 23.65, 7.93, 7.87; MS (ESI): *m*/*z* = calcd. For C_26_H_36_N_4_O_2_Se_2_ [M^+^]: 594.5, found 594.9 [M^+^], 616.9 [M^+^ + Na + H].

##### 4.1.1.11 The synthesis of 1-(4-(allylselanyl)phenyl)-3-((5-(3-(4-(allylselanyl)phenyl)ureido)-1,3,3-trimethylcyclohexyl)methyl)urea (HB199)

Compound HB199 was synthesized from OSe amine 5 (20 mmol) and IPDI (10 mmol) in toluene (10 mL) and separated as white powder; yield = 50%; MP = 209–210 °C; FT-IR (*ν*, cm^−1^): 3360, 2925, 2947, 2896, 1645, 1586, 1538, 1490, 1459, 1309, 1232, 1176, 815, 607, 502; ^1^H-NMR (600 MHz, DMSO-*d*_6_) *δ* 8.42 (s, 1H, NH), 8.30 (s, 1H, NH), 7.35–7.25 (m, 8H, Ar–H), 6.21 (t, *J* = 6.2 Hz, 2H, 2CH=), 5.96 (d, *J* = 7.9 Hz, 4H, 2CH_2_), 5.85 (tt, *J* = 9.3, 7.6 Hz, 1H, CH), 4.93–4.82 (m, 4H, 2CH_2_), 3.82–3.71 (m, 1H, CH), 3.47 (d, *J* = 7.5 Hz, 4H, CH), 2.89 (dd, *J* = 13.2, 6.5 Hz, 1H, CH), 2.80 (dd, *J* = 13.3, 5.9 Hz, 2H, CH_2_), 2.48 (dd, *J* = 3.6, 1.8 Hz, 1H, CH), 1.57 (dd, *J* = 24.5, 12.1 Hz, 2H, CH_2_), 1.15 (d, *J* = 13.7 Hz, 1H, CH), 1.04 (s, 1H, CH), 1.02 (s, 3H, CH_3_), 0.99 (s, 3H, CH_3_), 0.90 (s, 3H, CH_3_), 0.86 (dd, *J* = 22.5, 10.1 Hz, 2H, CH_2_); ^13^C-NMR (150 MHz, DMSO-*d*_6_) *δ* 155.69, 154.77, 140.52, 140.46, 135.27, 134.65, 134.56, 120.00, 118.62, 118.52, 117.00, 53.23, 47.36, 46.56, 45.09, 42.91, 42.24, 36.57, 35.42, 32.02, 30.88, 30.84, 28.00, 23.67; MS (ESI): *m*/*z* = calcd. For C_30_H_40_N_4_O_2_Se_2_ [M^+^]: 646.5, found 646.7 [M^+^], 668.8 [M^+^ + Na + H].

##### 4.1.1.12 The synthesis of 1-(4-(benzylselanyl)phenyl)-3-((5-(3-(4-(benzylselanyl)phenyl)ureido)-1,3,3-trimethylcyclohexyl)methyl)urea (HB206)

Compound HB206 was synthesized from OSe amine 6 (20 mmol) and IPDI (10 mmol) in toluene (10 mL) and separated as white powder; yield = 49%; MP = 276–277 °C;^1^H-NMR (400 MHz, DMSO-*d*_6_) *δ* 8.43 (s, 1H, NH), 8.38 (s, 1H, NH), 7.25 (d, *J* = 5.1 Hz, 7H, Ar–H), 7.21–7.16 (m, 5H, Ar–H), 7.16–7.11 (m, 6H, Ar–H), 6.27 (s, 1H, NH), 5.94 (s, 1H, NH), 4.14 (dd, *J* = 7.5, 4.6 Hz, 1H, CH), 4.05 (s, 4H, 2CH_2_), 3.79 (dd, *J* = 28.9, 20.6 Hz, 2H, CH_2_), 2.90–2.75 (m, 2H, CH_2_), 1.56 (t, *J* = 13.6 Hz, 2H, CH_2_), 1.20–1.05 (m, 2H, CH_2_), 1.00 (d, *J* = 3.3 Hz, 3H, CH_3_), 0.96 (d, *J* = 6.5 Hz, 3H, CH_3_), 0.88 (s, 3H, CH_3_); ^13^C-NMR (100 MHz, DMSO-*d*_6_) *δ* 155.66, 154.73, 140.53, 139.51, 134.58, 134.52, 129.12, 128.62, 126.99, 120.48, 118.56, 118.52, 118.43, 47.33, 46.55, 46.27, 42.87, 42.80, 36.54, 35.40, 31.99, 27.98, 23.65; MS (ESI): *m*/*z* = calcd. For C_38_H_44_N_4_O_2_Se_2_ [M^+^]: 746.7, found 746.0 [M^+^], 770.6 [M^+^ + Na–H].

##### 4.1.1.13 The synthesis of 1-(3-isocyanato-4-methylphenyl)-3-(4-(methylselanyl)phenyl)urea (HB191)

Compound HB191 was synthesized from OSe amine 4 (20 mmol) and 2,4-TDI (10 mmol) in toluene (10 mL) and separated as white powder; yield = 67%; MP = 285–286 °C; FT-IR (*ν*, cm^−1^): 3282, 2925, 2278, 1639, 1585, 1540, 1489, 1448, 1391, 1220, 1072, 813, 501; ^1^H-NMR (500 MHz, DMSO-*d*_6_) *δ* 8.51 (s, 1H, NH), 7.37 (d, *J* = 8.6 Hz, 3H, Ar–H), 7.34–7.32 (m, 3H, Ar–H), 7.04 (dd, *J* = 8.4, 4.4 Hz, 1H, Ar–H), 6.77 (s, 1H, NH), 2.29 (s, 6, 2CH_3_); ^13^C-NMR (125 MHz, DMSO-*d*_6_) *δ* 152.82, 147.14, 138.13, 131.64, 131.60, 130.65, 130.33, 123.14, 122.88, 119.43, 119.28, 115.50, 106.96, 104.63, 17.26, 7.82; MS (ESI): *m*/*z* = calcd. For C_16_H_15_N_3_O_2_Se [M^+^]: 360.2, found 359.9 [M^+^].

##### 4.1.1.14 Synthesis of 1-(4-(allylselanyl)phenyl)-3-(3-isocyanato-4-methylphenyl)urea (HB204)

Compound HB204 was synthesized from OSe amine 5 (20 mmol) and 2,4-TDI (10 mmol) in toluene (10 mL) and separated as white powder; yield = 82%; MP = 204–205 °C; FT-IR (*ν*, cm^−1^): 3279, 2923, 2278, 1639, 1584, 1531, 1487, 1217, 1052, 813, 752, 640, 500; ^1^H-NMR (600 MHz, DMSO-*d*_6_) *δ* 8.62 (s, 1H, NH), 8.55 (s, 1H, NH), 7.97–7.86 (m, 1H, Ar–H), 7.45–7.33 (m, 4H, Ar–H), 7.21–7.11 (m, 1H, Ar–H), 7.08–7.00 (m, 1H, Ar–H), 5.87 (ddtd, *J* = 11.2, 10.0, 7.6, 3.7 Hz, 1H, CH=), 4.97–4.86 (m, 2H, CH_2_=), 3.51 (dd, *J* = 7.4, 4.0 Hz, 2H, SeCH_2_), 2.16 (s, 3H, CH_3_); ^13^C-NMR (150 MHz, DMSO-*d*_6_) *δ* 152.78, 139.76, 139.67, 138.15, 137.89, 135.23, 134.56, 134.49, 130.66, 121.13, 121.10, 119.17, 119.06, 117.08, 30.79, 17.68; MS (ESI): *m*/*z* = calcd. For C_18_H_17_N_3_O_2_Se [M^+^]: 386.3, found 385.7 [M^+^ − H].

##### 4.1.1.15 The synthesis of 1-(4-(benzylselanyl)phenyl)-3-(3-isocyanato-4-methylphenyl)urea (HB205)

Compound HB205 was synthesized from OSe amine 6 (20 mmol) and 2,4-TDI (10 mmol) in toluene (10 mL) and separated as white powder; yield = 49%; MP = 277–278 °C; FT-IR (*ν*, cm^−1^): 3339, 3028, 2269, 1643, 1586, 1488, 1538, 1520, 1451, 1417, 1217, 1177, 814, 693, 500; ^1^H-NMR (400 MHz, DMSO-*d*_6_) *δ* 9.14 (s, 1H, NH), 8.57 (s, 1H, NH), 7.39–7.27 (m, 4H, Ar–H), 7.26–7.08 (m, 6H, Ar–H), 7.05–6.98 (m, 1H, Ar–H), 6.76 (d, *J* = 8.4 Hz, 1H, Ar–H), 4.15 (s, 2H, CH_2_), 2.22 (s, 3H, CH_3_); ^13^C-NMR (100 MHz, DMSO-*d*_6_) *δ* 152.74, 139.89, 139.79, 139.72, 139.46, 138.38, 138.11, 137.86, 134.44, 130.64, 130.38, 129.15, 128.65, 127.03, 121.57, 118.93, 31.92, 17.21; MS (ESI): *m*/*z* = calcd. For C_22_H_19_N_3_O_2_Se [M^+^]: 436.3, found 435.7 [M^+^ − H], 491.7 [M^+^ + Na + MeOH].

### 4.2. Biological evaluation

The biological assays were conducted in the Cancer Biology Department, National Cancer Institute, Cairo University.

#### 4.2.1. The percentage of cellular inhibition of growth (% GI) assay toward several normal and tumor cell lines

To evaluate the anticancer potential of newly synthesized OSe derivatives, a sulforhodamine B (SRB) colorimetric assay (full protocols are available in the Supplementary Information). Every cancer cell line utilized in this investigation was procured from Vacsera (Giza, Egypt) and included a diverse panel of eight human tumor cell lines: HN9, HuH7, HEPG2, FaDu, MCF7, A549, HCT_116_, and A375, all originally obtained from the American Type Culture Collection (ATCC).

To assess the safety and selectivity of these selenium-based compounds, simultaneous cytotoxicity assays were conducted on noncancerous human cells, including HSF and OEC. HSFs are widely employed in research related to skin biology, regenerative medicine, and oncology. OECs are a good way to check compound selectivity and biocompatibility because they can grow back to normal and are useful in models of oral disease.

#### 4.2.2. *In vitro* cytotoxicity evaluation

To gain a deeper insight into the cytotoxic profiles of the most promising compounds—HB188, HB199, HB204, and HB206—their IC_50_ values were assessed in particular cancer cell lines. These were HEPG2, A549, HCT_116_, HuH7, FaDu, and MCF7, which, based on the first screening tests, had the highest rates of inhibition. The SRB assay was used to determine the IC_50_ values. The full protocols can be found in the SI. The results showed us how strong the compound was against a variety of tumors.

#### 4.2.3. Apoptosis-related protein expression

To elucidate the apoptosis-inducing mechanism of HB204, protein expression profiling was conducted in the FaDu cancer cell line, noted for its high treatment responsiveness. The focus was on the main regulators of the apoptotic cascade, such as the pro-apoptotic proteins BAX, caspase-3, -7, and -9, and the anti-apoptotic protein BCL-2. Commercially available ELISA and immunoblotting kits (Catalog Nos: SEA626Hu, EH71RB, ab119508, SEA778Ra) were utilized. The levels of matrix metalloproteinases MMP2 and MMP9 (Catalog Nos: MBS9135926 and MBS175780, respectively) were also investigated to see how the compound might influence invasion and metastasis. A comparative analysis of treated and untreated cells provided mechanistic insights into the apoptotic pathways and metastatic suppression triggered by HB204.

#### 4.2.4. Cell cycle arrest determination

To obtain more profound insights into the effects of HB204 on cancer cell proliferation, the cell cycle distribution was analyzed through flow cytometry. FaDu cells (2 × 10^5^ cells/well) were seeded in 12-well plates and treated with growing concentrations of HB204 for different time periods. After treatment, cells were fixed in cold 70% ethanol and stained with propidium iodide (PI) in the presence of RNase A and Triton X-100. A Becton Dickinson flow cytometer with a 488 nm laser was then used to examine the DNA content. This made it possible to count cells in the G_0_/G_1_, S, and G_2_/M phases, which showed any cell cycle arrest triggered by the compound being studied.

#### 4.2.5. Apoptosis analysis using annexin V-FITC/PI staining

The apoptotic effect of HB204 was evaluated in FaDu cells using Annexin V-FITC/Propidium Iodide (PI) double staining followed by flow cytometric analysis.^69,70^ Briefly, FaDu cells were seeded and allowed to attach overnight, then treated with HB204 for 48 h. After incubation, cells were harvested, washed twice with cold phosphate-buffered saline (PBS), and resuspended in binding buffer. Subsequently, Annexin V-FITC and PI solutions were added according to the manufacturer's protocol, and the cells were incubated in the dark for 15 min at room temperature. Samples were immediately analyzed by flow cytometry. Cell populations were classified into viable, early apoptotic, late apoptotic, and necrotic cells based on Annexin V and PI staining patterns. Data were expressed as mean ± SD from three independent experiments and statistically analyzed using two-way ANOVA followed by Sidak's multiple-comparison test. Differences were considered statistically significant at *P* < 0.05.

### 4.3. *In silico* studies

#### 4.3.1. Molecular docking

The AutoDock Vina 1.2.0 (ref. 71) and PyMOL^72^ programs were used to dock the best lead, HB204, to the BAX receptor after it was prepared by minimizing energy and optimizing partial charge.^73,74^ The protein receptor with ID (1F16) was found in the PDB.^75^ The reported BAX small allosteric inhibitor (BAI 1)^64^ was compared with the lead HB204 for comparison in terms of binding mode and score.^76,77^

#### 4.3.2. Molecular dynamics (MD) simulation

The MD simulation was performed using the Desmond simulation package of Schrödinger LLC^78^ for 500 ns (SI (SI3)).

## Author Contributions

Conceptualization and supervision: Ahmed A. Al-Karmalawy and Saad Shaaban; data collection, data curation, visualization, methodology, and writing – review and editing: Saad Shaaban, Samia S. Hawas, Ohud alzaidi, Marwa Sharaky, Fatema S. Alatawi, Khadra B. Alomari, Zainab S. Alghamdi, Hussein Ba-Ghazal, Arwa Omar Al Khatib, Hany M. Abd El-Lateef, Tarek A. Yousef, Mohamed Alaasar, and Ahmed A. Al-Karmalawy. All authors approved the submitted version of the manuscript.

## Conflicts of interest

The authors declared no conflict of interest.

## Funding

This work was supported by the Deanship of Scientific Research, Vice Presidency for Graduate Studies and Scientific Research, King Faisal University, Saudi Arabia [Grant No. KFU263720].

## Supplementary Material

RA-OLF-D6RA03577A-s001

## Data Availability

The data supporting this article have been included in the manuscript and as part of the supplementary information (SI). Supplementary information is available. See DOI: https://doi.org/10.1039/d6ra03577a.
