# Supplementary material for: Novel seleno-ureido hybrids targeting head and neck cancer and beyond: design, synthesis, and apoptosis-mediated anticancer evaluation
Source: RSC Adv. 2026 Jul 15. Online ahead of print. doi: 10.1039/d6ra03577a (PMC13370745; doi:10.1039/d6ra03577a)
Supplement: RA-OLF-D6RA03577A-s001 [file RA-OLF-D6RA03577A-s001.pdf]

This journal is © The Royal Society of Chemistry 2026

Novel Seleno-Ureido Hybrids Targeting Head and Neck Cancer and Beyond: Design, Synthesis, and Apoptosis-Mediated Anticancer Evaluation

Saad Shaaban[1,¥], Samia S. Hawas[2,¥], Ohud alzaidi[3], Marwa Sharaky[4], Fatema S. Alatawi[5], Khadra B. Alomari[6], Zainab S. Alghamdi[7], Hussein Ba-Ghazal[1], Arwa Omar Al Khatib[8], Hany M. Abd El-Lateef[1], Tarek A. Yousef[9], Mohamed Alaasar[10], Ahmed A. Al-Karmalawy[2,*]

[1] Department of Chemistry, College of Science, King Faisal University, Al-Ahsa 31982, Saudi Arabia.
[2] Department of Pharmaceutical Chemistry, Faculty of Pharmacy, Horus University-Egypt, New Damietta 34518, Egypt.
[3] Department of Chemistry, College of Science-Al Khurma, Taif University, Taif 21944, Saudi Arabia.
[4] Cancer Biology Department, National Cancer Institute (NCI), Cairo University, Cairo, Egypt.
[5] Department of Biochemistry, Faculty of Science, University of Tabuk, Tabuk, Saudi Arabia.
[6] Jazan University, Department of Physical Sciences, Chemistry Division, Jazan 45142, Kingdom of Saudi Arabia.
[7] Department of Chemistry, College of Science, Imam Abdulrahman Bin Faisal University, Dammam 31441, Saudi Arabia.
[8] Faculty of Pharmacy, Al-Ahliyya Amman University, Amman, Jordan.
[9] College of Science, Chemistry Department, Imam Mohammad Ibn Saud Islamic University (IMSIU), Riyadh 11623, Saudi Arabia.
[10] Department of Chemistry, Faculty of Science, Cairo University, Giza, Egypt.

* Correspondence:

Ahmed A. Al-Karmalawy: Email: akarmalawy@horus.edu.eg

Saad Shaaban: Email: sibrahim@kfu.edu.sa

Chemistry: Methods and Materials

All solvents and reagents were purchased from Sigma-Aldrich and were used without any prior purification. Melting points (MP) in ºC were recorded on the Gallenkamp instrument. The IR spectra (ATR, ύ cm^{-1}) were recorded at King Faisal University on a Mattson 5000 FTIR Spectrophotometer.

The structure characterization of the prepared materials is based on ^{1}H-NMR, ^{13}C-NMR (Agilent Technologies 400, 500, and 600 MHz VNMRS in DMSO-d_6 solution, with tetramethylsilane as internal standard. The high-resolution mass spectra were measured at Martin Luther University, Halle, Germany, on Thermo Scientific Q Exactive Plus mit HESI, APCI, and LIFDI-Quelle (Linden CMS).

HPLC analysis was carried out using a Shimadzu Prominence-i LC-2030C 3D Plus system equipped with a PDA detector. Chromatographic separation was achieved on a C18 column using an isocratic mobile phase of acetonitrile/water (90:10, v/v) at a flow rate of 0.6 mL min^{-1}. The run time was 10 min.

OSe amines **2**-**6** were synthesized following our reported method (experimental details are also listed in the Supporting Information) [1-4].

4-Selenocyanatoaniline (2) [5]

Selenium dioxide (6 mmol) was added under stirring to a solution of malononitrile (3 mmol) in DMSO (15 mL). The mixture was stirred at room temperature for 15 min in order to obtain triselenium dicyanide. When the exothermic reaction had finished, aniline (5 mmol) was added. The mixture was stirred for 20 min. Water (150 mL) was added to the reaction mixture, and the resulting precipitate (4-selenocyanatobenzenamine) was filtered off, dried, and used without further purifications. 4-Aminophenylselenocyanate (**2**) is a yellow solid, melting point = 73–74 °C. ^{1}H NMR (400 MHz, $CDCl_3$) δ 7.44 (d, J = 8.4 Hz, 2H, Ar-H), 6.64 (d, J = 8.4 Hz, 2H, Ar-H), 3.95 (s, 2H, NH_2).

Synthesis of 4,4'-diselanediyldianiline (3) [5]

Under argon, $NaBH_4$ (3 mmol) was added in small portions with caution to a solution of 4-selenocyanatobenzenamine (**2**) (1 mmol) in absolute ethanol (40 mL). The mixture was stirred at room temperature for 2 h. The solvent was removed under reduced pressure, and the remaining residue was dissolved in dichloromethane, washed with water (350 mL). The organic layer was

separated, dried with anhydrous Na_2SO_4, and removed under vacuum. The residue was purified by chromatography on silica gel (petroleum ether: ethyl acetate 4:2). 4,4′-Diselanediyldianiline (**3**) was obtained as pale-yellow crystals (82% yield. Mp: 78–80 °C. ^{1}H NMR (400 MHz, $CDCl_3$) δ 7.3 (m, 4H, Ar-H), 6.5 (m, 4H, Ar-H), 3.7 ppm (s, 4H, NH_2).

Synthesis of Synthesis of 4-(methylselanyl)aniline (4) [6]

4-(methylselanyl)aniline (**4**) was prepared in 57 % yield from 4,4′-diselanediyldianiline (**3**) (2 mmol), methyl iodide (4.4 mmol), NaOH (2 mol), and $NaBH_4$ (50 mmol) in MeOH (30 mL). The reaction continued at RT for approximately 2 hr. The reaction was extracted using CH_2Cl_2 and removed under vacuum to give a brown oil in 91 % yield. 4-(Methylselanyl)aniline (**4**) was synthesized as a brown oil (91% yield). ^{1}H NMR (400 MHz, $CDCl_3$) δ 7.31 (d, J = 9.0 Hz, 2H, Ar-H), 6.61(d, J = 9.0 Hz, 2H, Ar-H), 3.72 (br s, 2H, NH_2), 2.66 (s, 3H, CH_3).

Synthesis of 4-(allylselanyl)aniline (**5**)

A mixture of dimethyl 4,4'-diselanediyldianiline (**3**) (2 mmol), allyl bromide (4.4 mmol), and NaOH was dissolved in ethanol (25 mL). Sodium borohydride (6 mmol) was then introduced gradually over the course of one hour, and the reaction was stirred for an additional two hours. The organic phase was subsequently dried and concentrated under reduced pressure, affording the intermediate 4-(allylselanyl)aniline (**5**) as a brown oil, which was directly used in the following step without purification.

Synthesis of 4-(Benzylselanyl)aniline (6) [7]

Compound **6** was synthesized from 4,4′-diselanediyldianiline (**3**) (170 mg, 0.5 mmol), $PhCH_2Cl$ (126.5 ml, 1.1 mmol), Starks' catalyst (22.5 mg, 2.5% mol), and $NaBH_4$ (57 mg, 1.5 mmol). The product formation was followed by TLC petroleum ether: EtOAc = 6:1, Rf = 0.36, purified by column silica gel chromatography with petroleum ether: EtOAc = 6:1.5. Colourless oil; Yield: 224 mg (85%). 4-(Benzylselanyl)aniline (**6**) was isolated as colourless oil; Yield: 224 mg (85%). ^{1}H NMR (300 MHz, $CDCl_3$) 7.28-7.18 (m, 2H, Ar-H), 7.14-7.08 (m, 2H, Ar-H), 6.94-6.81 (m, 2H, Ar-H), 6.50-6.39 (m, 2H, Ar-H), 3.85 (s, 2H, $SeCH_2$), 3.62 (s, 2H, NH_2); ^{13}C NMR (100 MHz, $CDCl_3$) δ 146.68, 138.65, 137.03, 131.32, 130.48, 120.33, 116.48, 115.63, 32.51; MS (ESI): m/z = found 263.85 [M^++1]; calcd. 263.02 [M^+].

Copies of the ^{1}H-NMR & ^{13}C-NMR, IR, and MS spectra

1,1'-(1,4-phenylene)bis(3-(4-(methylselanyl)phenyl)urea) (HB197)

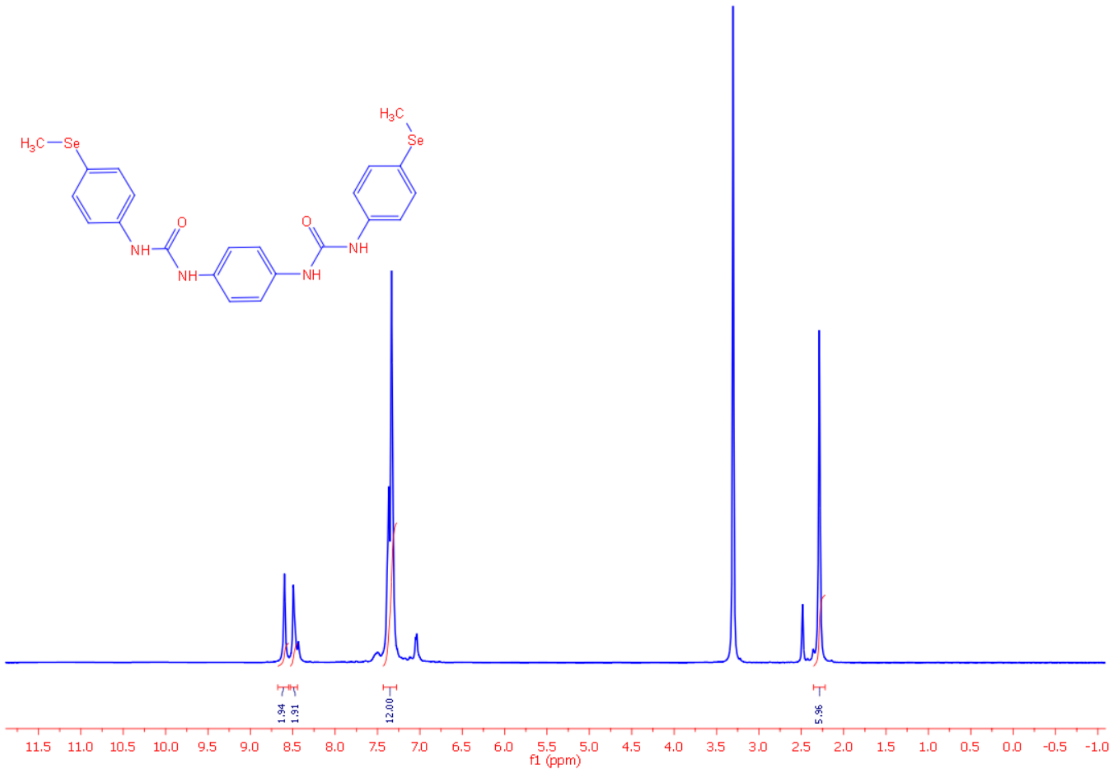

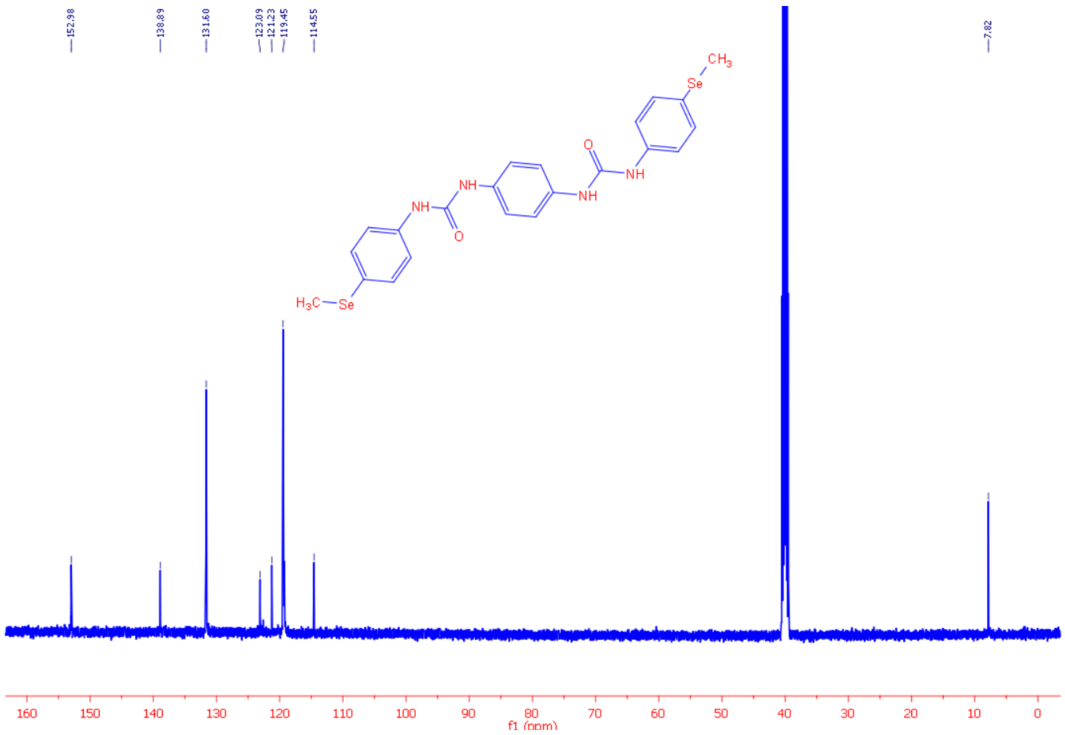

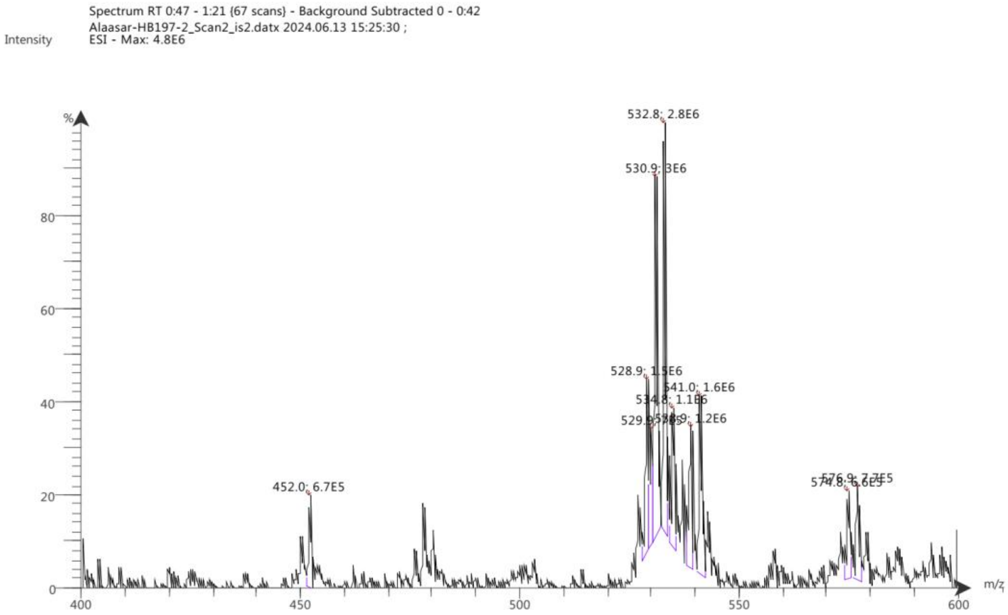
Spectrum RT 0:47 - 1:21 (67 scans) - Background Subtracted 0 - 0:42
Alaasar-HB197-2_Scan2_is2.datx 2024.06.13 15:25:30 ;
Intensity
ESI - Max: 4.8E6
532.8; 2.8E6
530.9; 3E6
528.9; 1.5E6
541.0; 1.6E6
534.8; 1.1E6
539.9; 1.2E6
452.0; 6.7E5
m/z

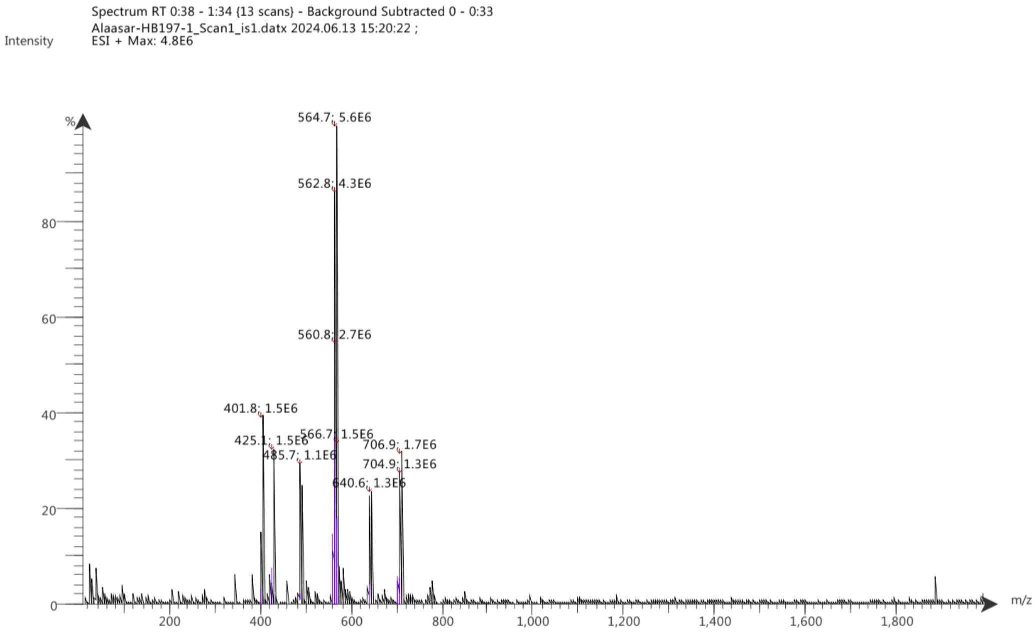
Spectrum RT 0:38 - 1:34 (13 scans) - Background Subtracted 0 - 0:33
Alaasar-HB197-1_Scan1_is1.datx 2024.06.13 15:20:22 ;
Intensity
ESI + Max: 4.8E6
564.7; 5.6E6
562.8; 4.3E6
560.8; 2.7E6
401.8; 1.5E6
425.1; 1.5E6
566.7; 1.5E6
485.7; 1.1E6
706.9; 1.7E6
704.9; 1.3E6
640.6; 1.3E6
m/z

1,1'-(1,4-phenylene)bis(3-(4-(allylselanyl)phenyl)urea) (HB193)

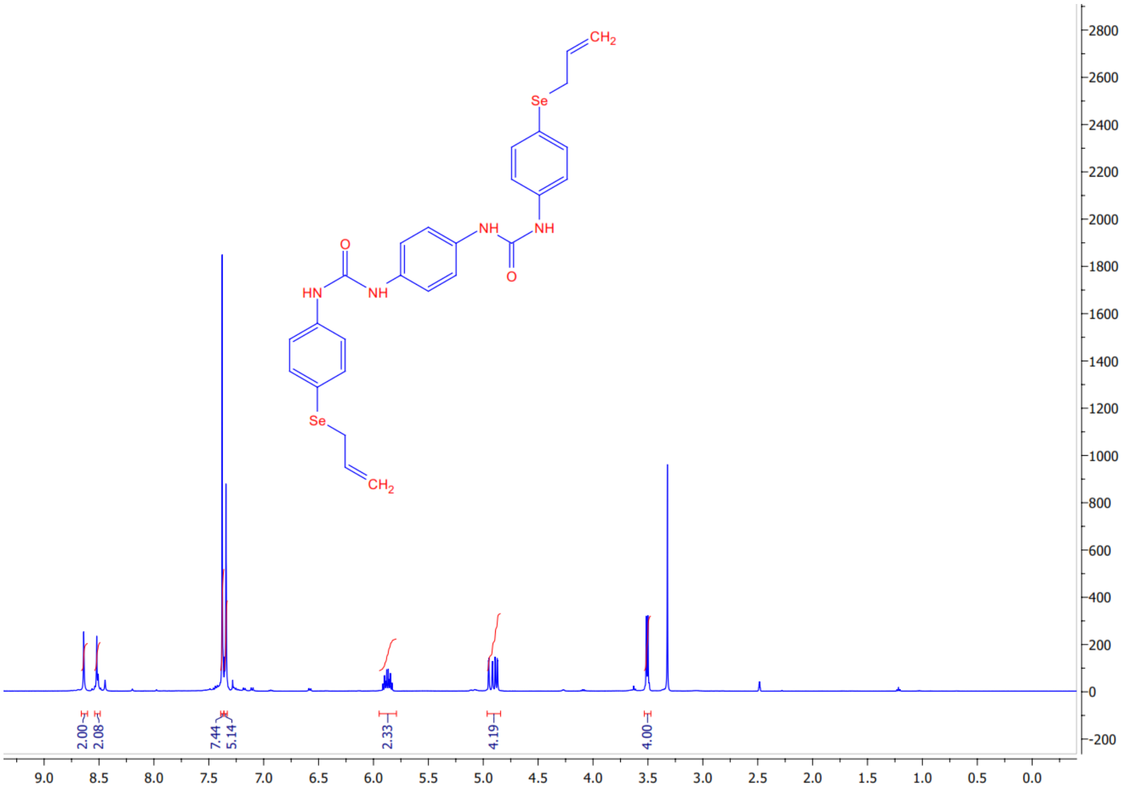

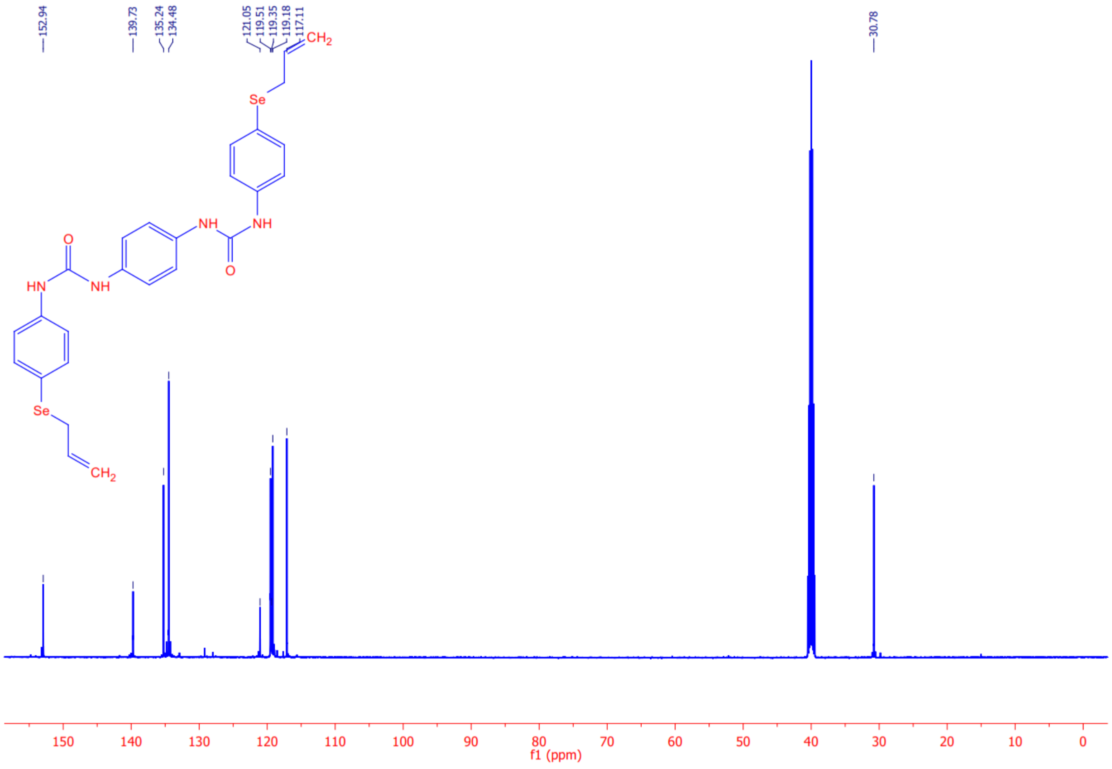

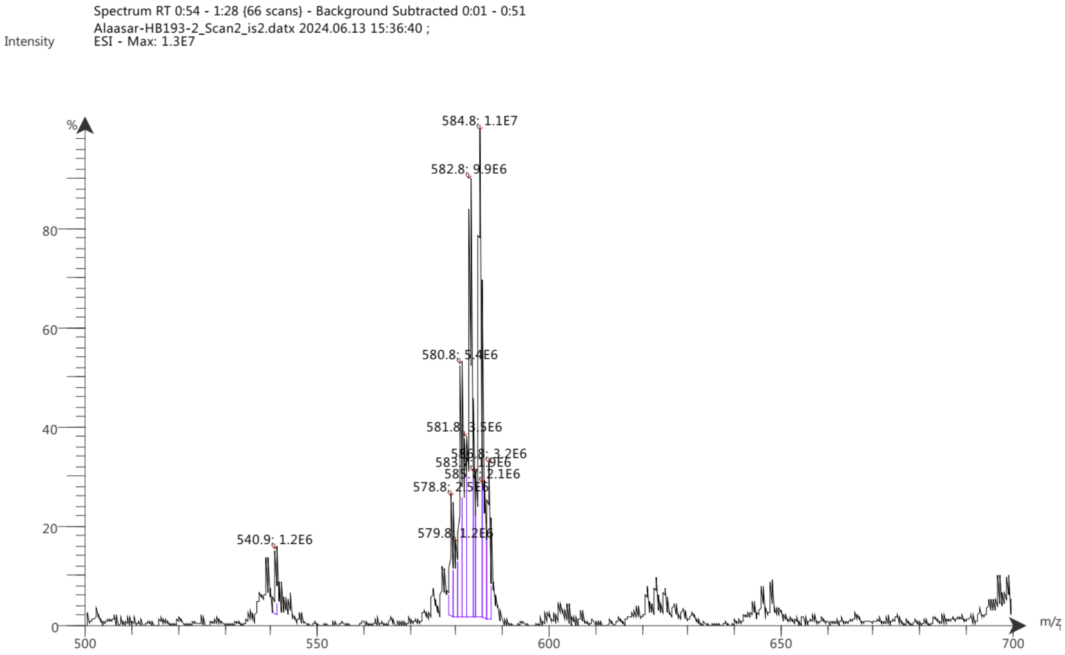
Spectrum RT 0:54 - 1:28 (66 scans) - Background Subtracted 0:01 - 0:51
Alaasar-HB193-2_Scan2_is2.datx 2024.06.13 15:36:40 ;
Intensity
ESI - Max: 1.3E7
584.8; 1.1E7
582.8; 9.9E6
580.8; 5.4E6
581.8; 3.5E6
578.8; 2.5E6
579.8; 1.2E6
540.9; 1.2E6
%
m/z

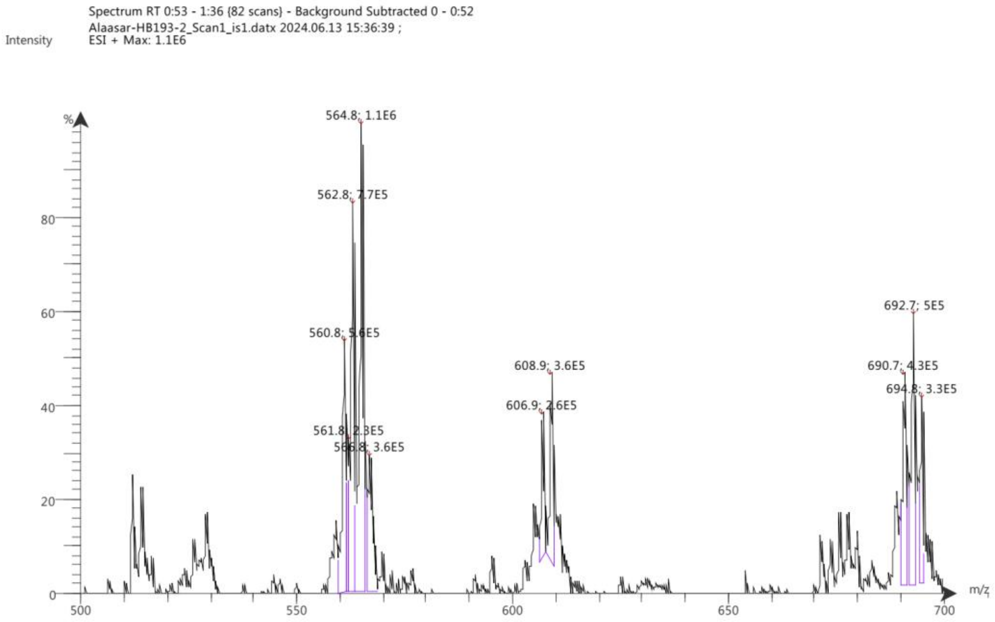
Spectrum RT 0:53 - 1:36 (82 scans) - Background Subtracted 0 - 0:52
Alaasar-HB193-2_Scan1_is1.datx 2024.06.13 15:36:39 ;
Intensity
ESI + Max: 1.1E6
564.8; 1.1E6
562.8; 7.7E5
560.8; 5.6E5
561.8; 2.3E5
566.8; 3.6E5
608.9; 3.6E5
606.9; 2.6E5
692.7; 5E5
690.7; 4.3E5
694.8; 3.3E5
%
m/z

1,1'-(1,4-phenylene)bis(3-(4-(benzylselanyl)phenyl)urea) (HB202)

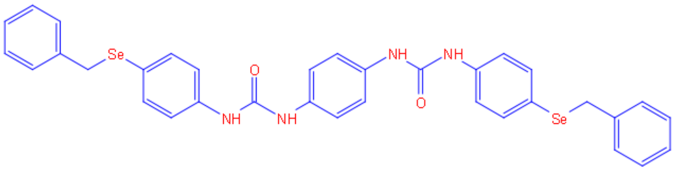

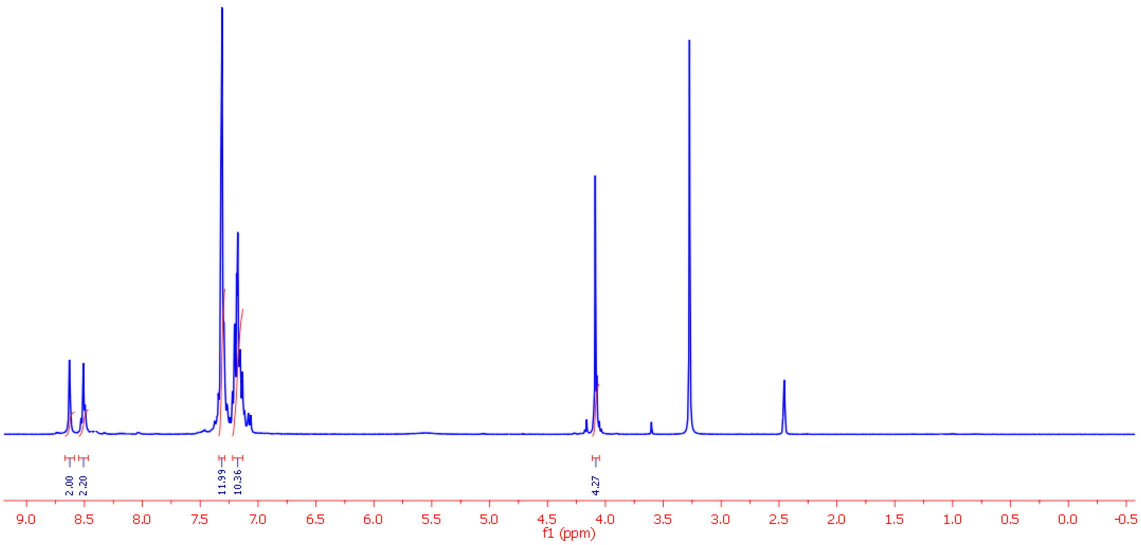

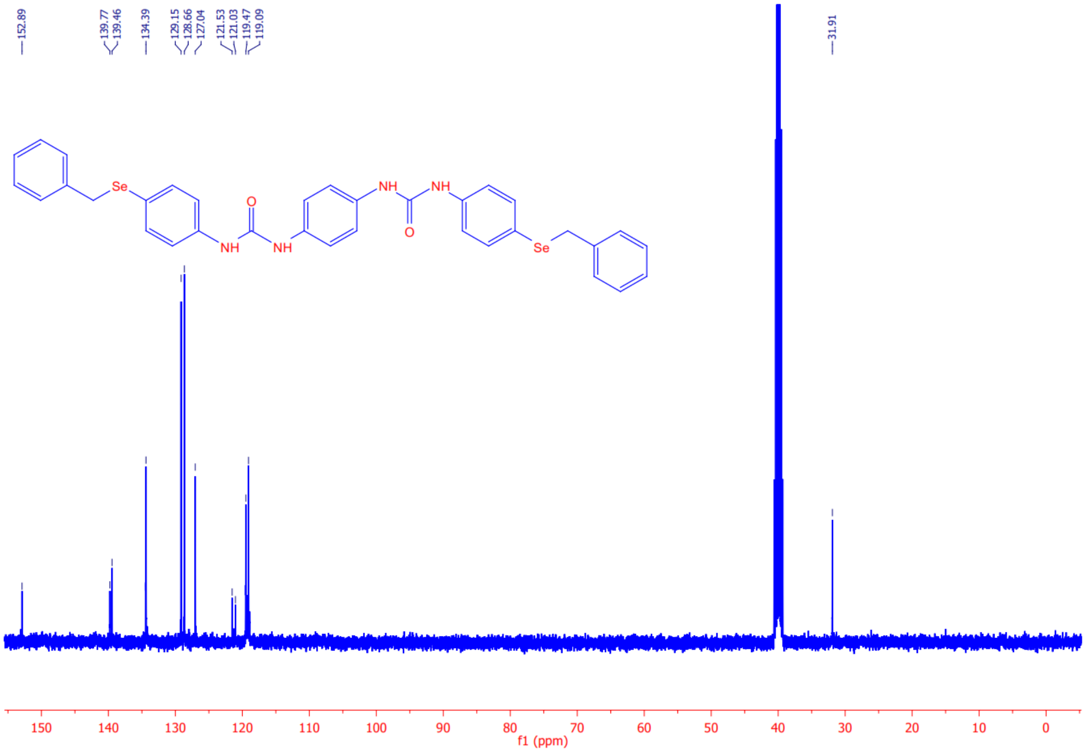

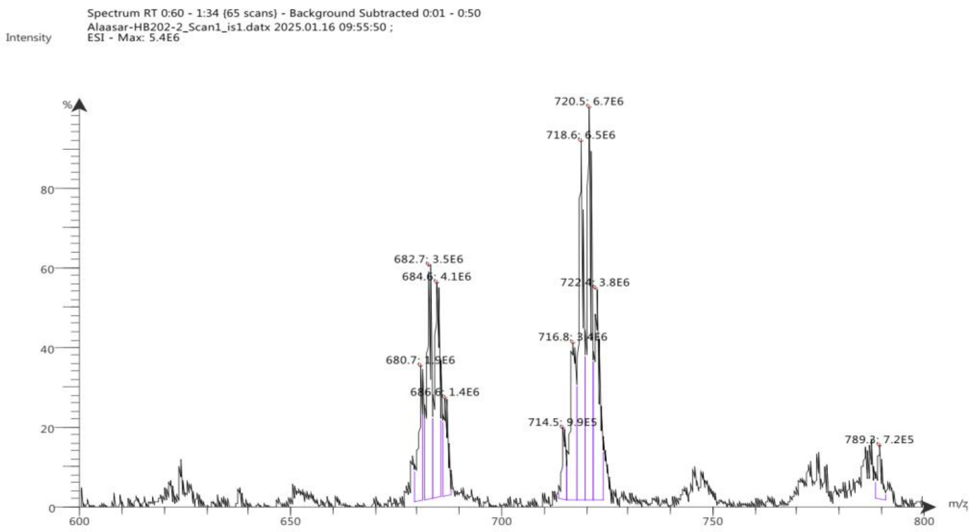
Spectrum RT 0:60 - 1:34 (65 scans) - Background Subtracted 0:01 - 0:50
Alaasar-HB202-2_Scan1_is1.datx 2025.01.16 09:55:50 ;
ESI - Max: 5.4E6
Intensity
720.5; 6.7E6
718.6; 6.5E6
682.7; 3.5E6
684.6; 4.1E6
722.4; 3.8E6
716.8; 3.4E6
680.7; 1.5E6
686.6; 1.4E6
714.5; 9.9E5
789.3; 7.2E5
m/z

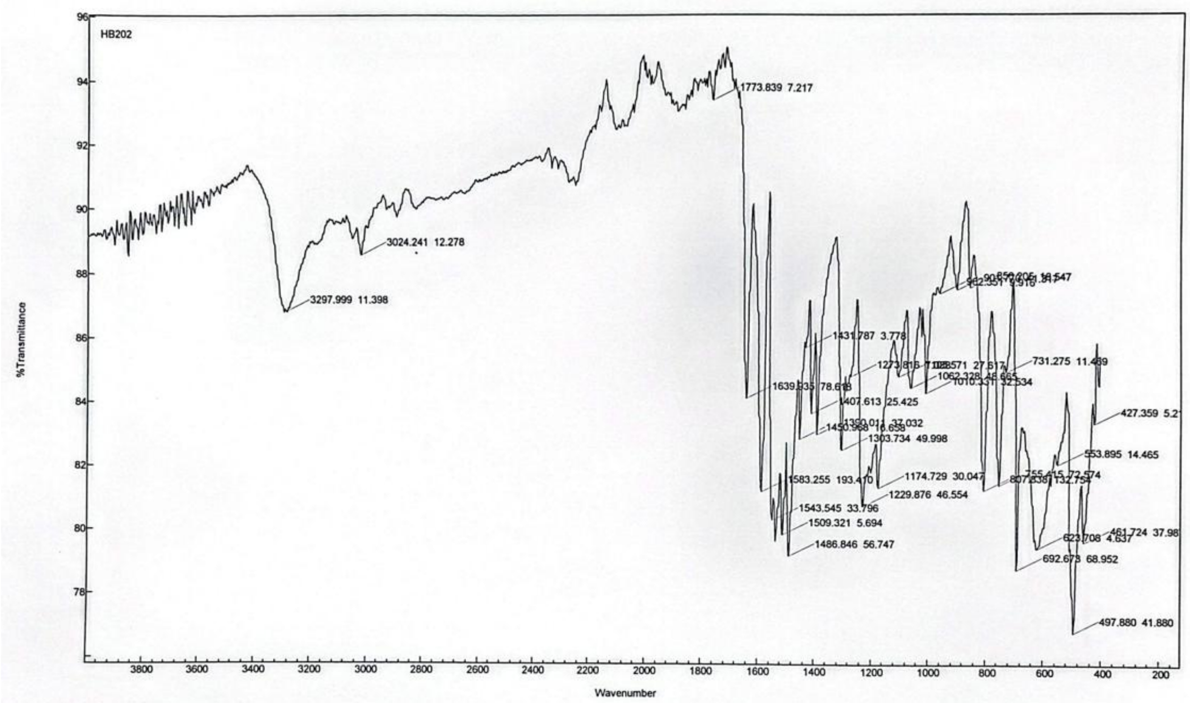
HB202
%Transmittance
Wavenumber
1773.839 7.217
3024.241 12.278
3297.999 11.398
1431.787 3.778
731.275 11.489
427.359
553.895 14.465
1407.613 25.425
1303.734 49.998
1583.255 193.410
1174.729 30.047
1229.876 46.554
1543.545 33.796
1509.321 5.694
1486.846 56.747
692.673 68.952
497.880 41.880
461.724 37.98

1,1'-(methylenebis(4,1-phenylene))bis(3-(4-(methylselanyl)phenyl)urea) (HB190)

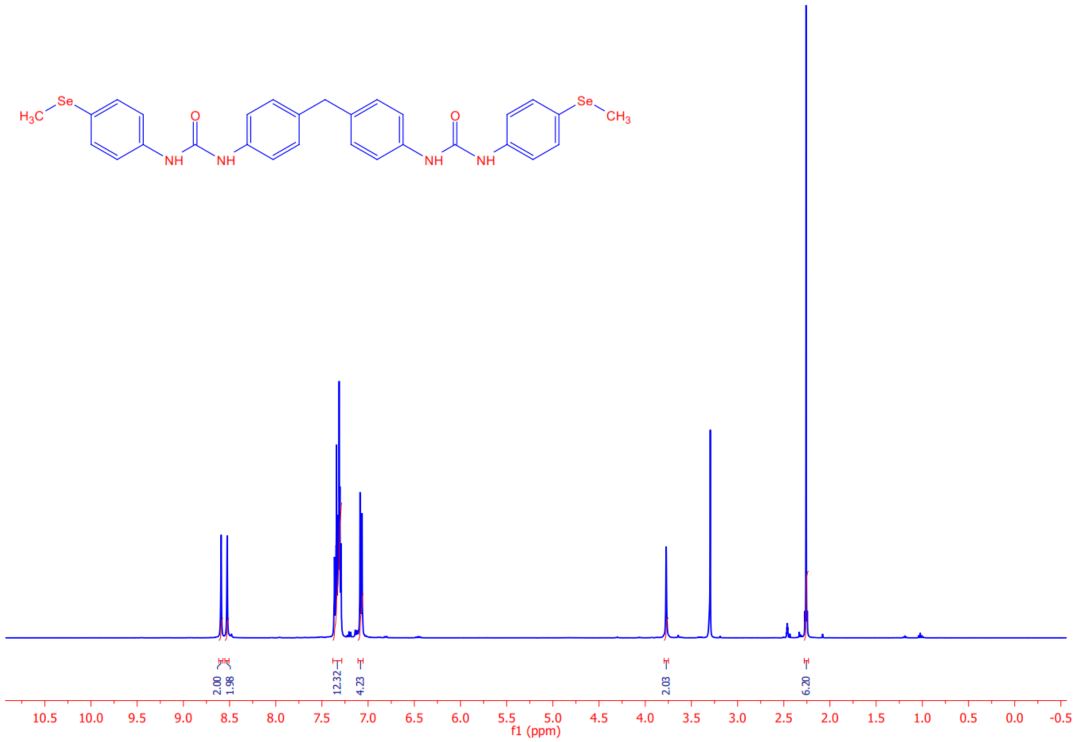

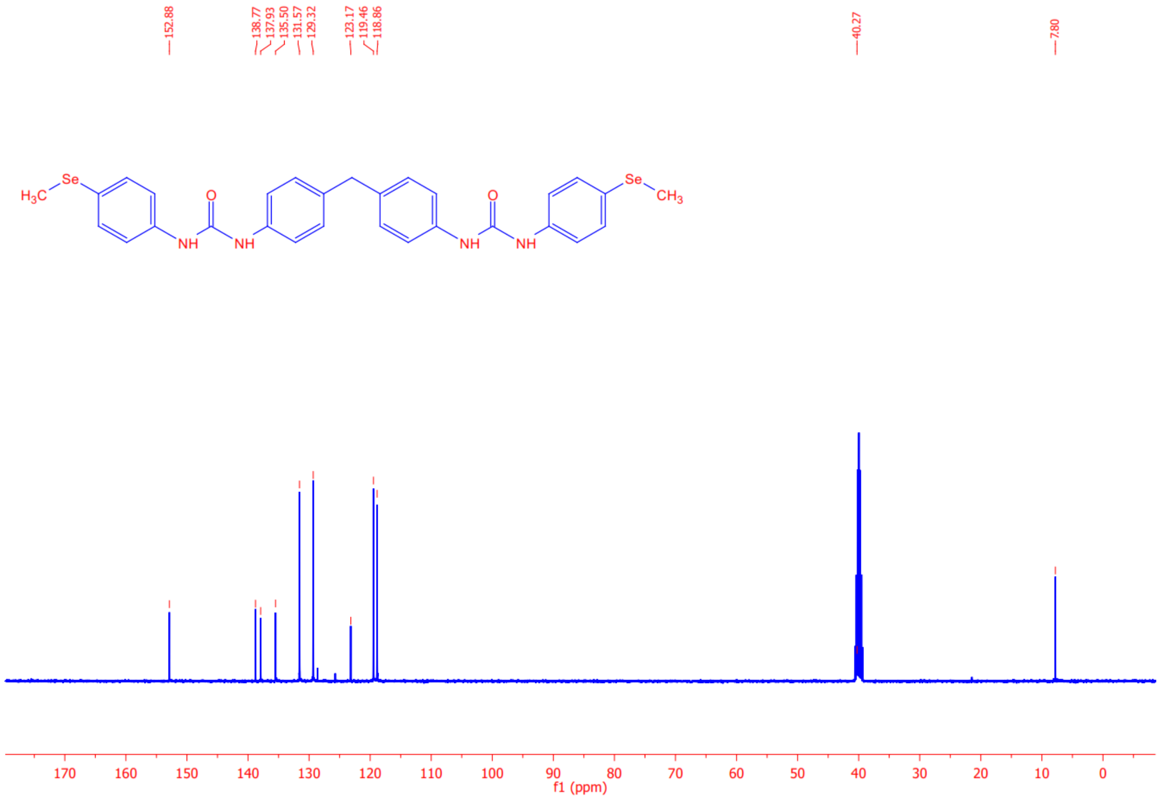

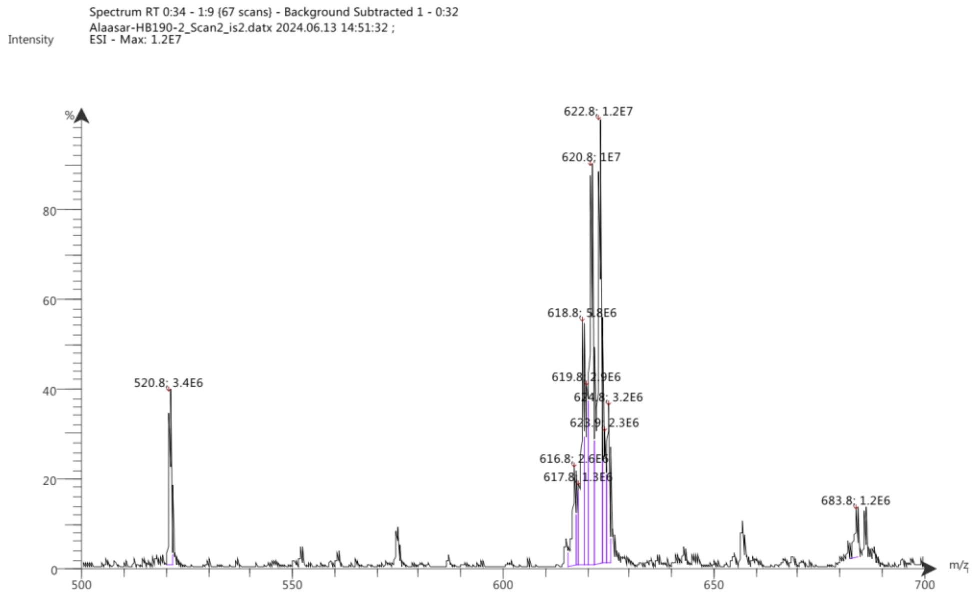

Spectrum RT 0:34 - 1:9 (67 scans) - Background Subtracted 1 - 0:32
Alaasar-HB190-2_Scan2_is2.datx 2024.06.13 14:51:32 ;
Intensity
ESI - Max: 1.2E7
622.8; 1.2E7
620.8; 1E7
618.8; 5.8E6
520.8; 3.4E6
619.8; 2.9E6
624.8; 3.2E6
623.9; 2.3E6
616.8; 2.6E6
617.8; 1.3E6
683.8; 1.2E6
m/z

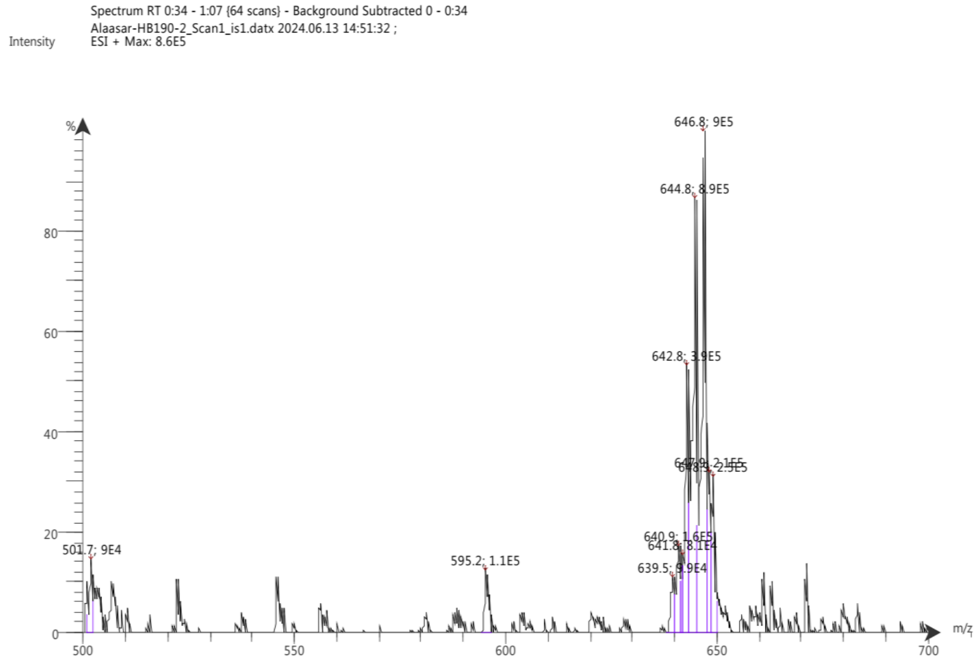

Spectrum RT 0:34 - 1:07 (64 scans) - Background Subtracted 0 - 0:34
Alaasar-HB190-2_Scan1_is1.datx 2024.06.13 14:51:32 ;
Intensity
ESI + Max: 8.6E5
646.8; 9E5
644.8; 8.9E5
642.8; 3.9E5
501.7; 9E4
595.2; 1.1E5
639.5; 9.9E4
m/z

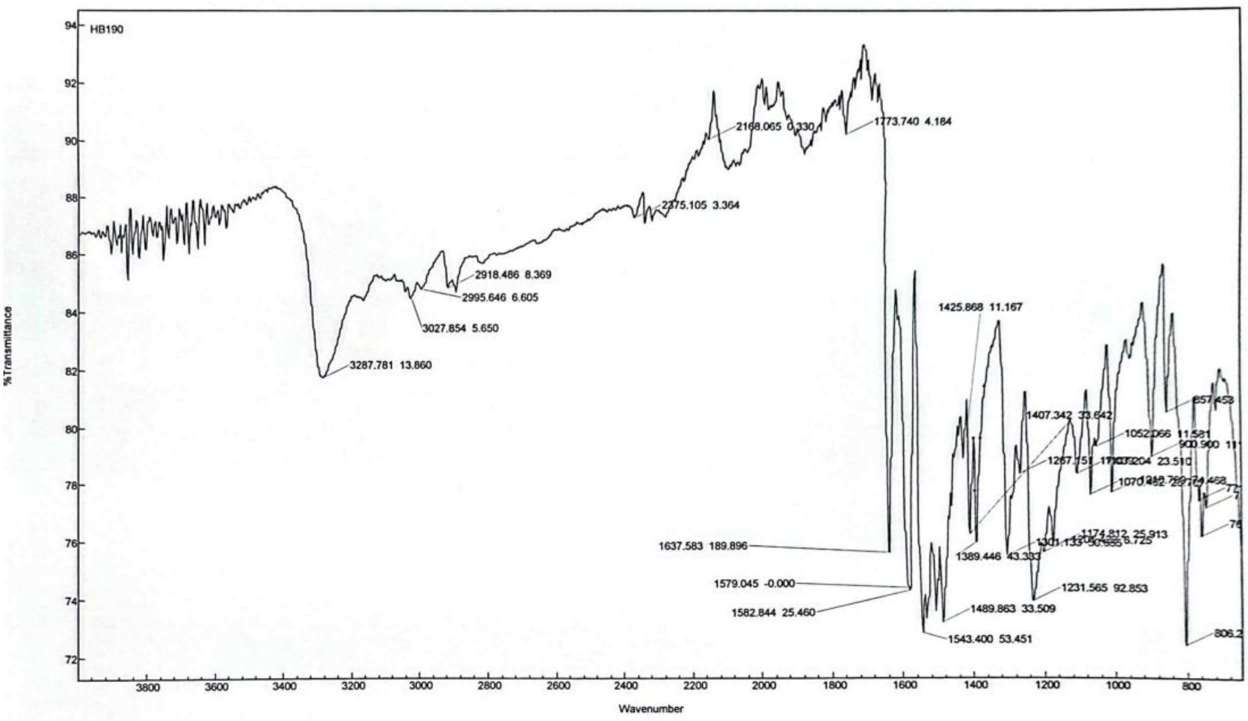
HB190
%Transmittance
Wavenumber
3287.781 13.860
3027.854 5.650
2995.646 6.605
2918.486 8.369
2375.105 3.364
2168.065 0.330
1773.740 4.184
1637.583 189.896
1579.045 -0.000
1582.844 25.460
1543.400 53.451
1489.863 33.509
1425.868 11.167
1407.342 33.642
1389.446 43.333
1231.565 92.853
1052.066 11.581

1,1'-(methylenebis(4,1-phenylene))bis(3-(4-(allylselanyl)phenyl)urea) (HB200)

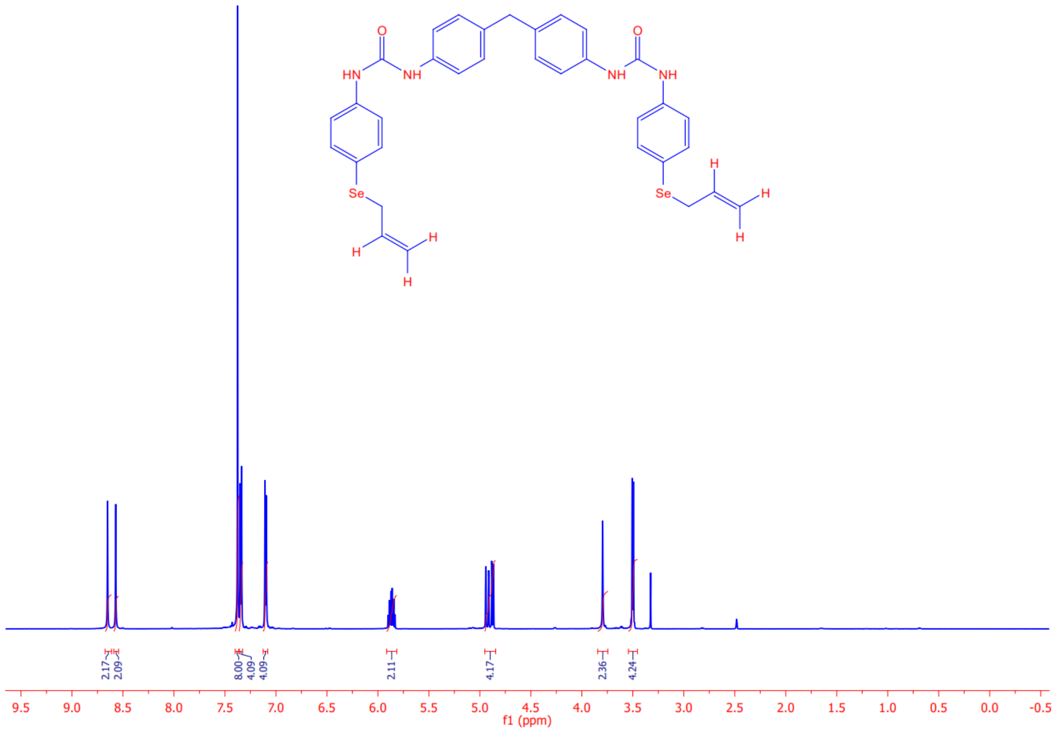

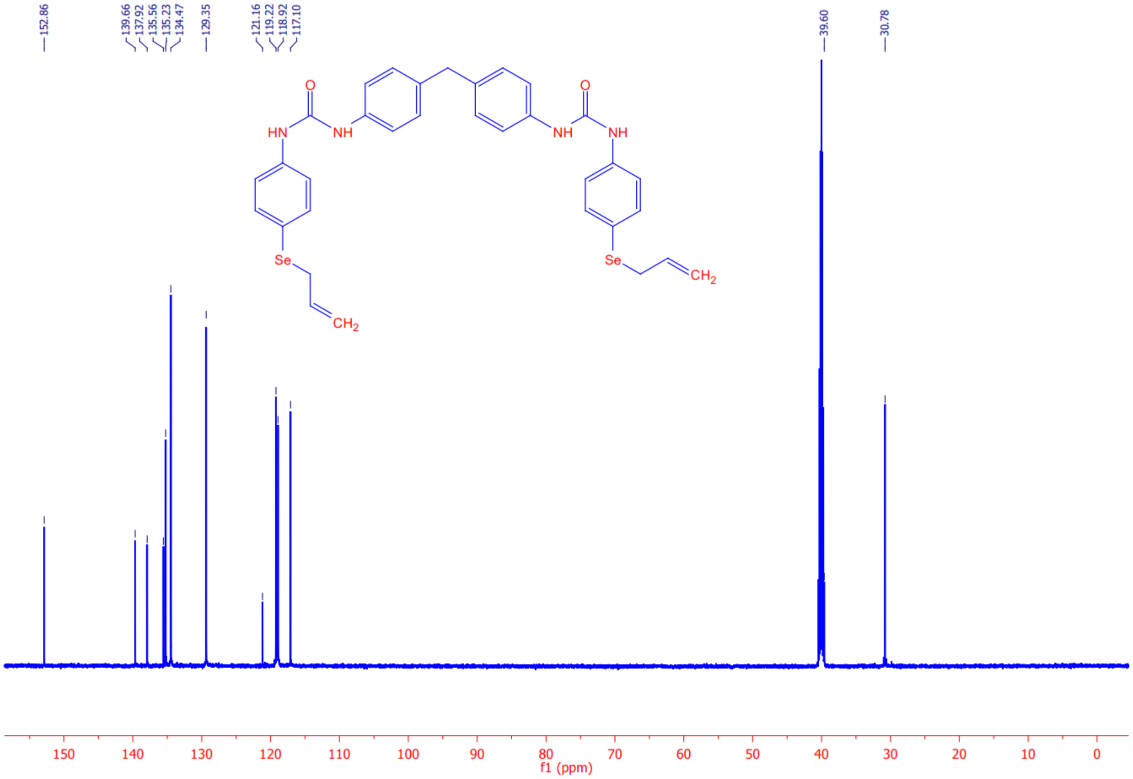

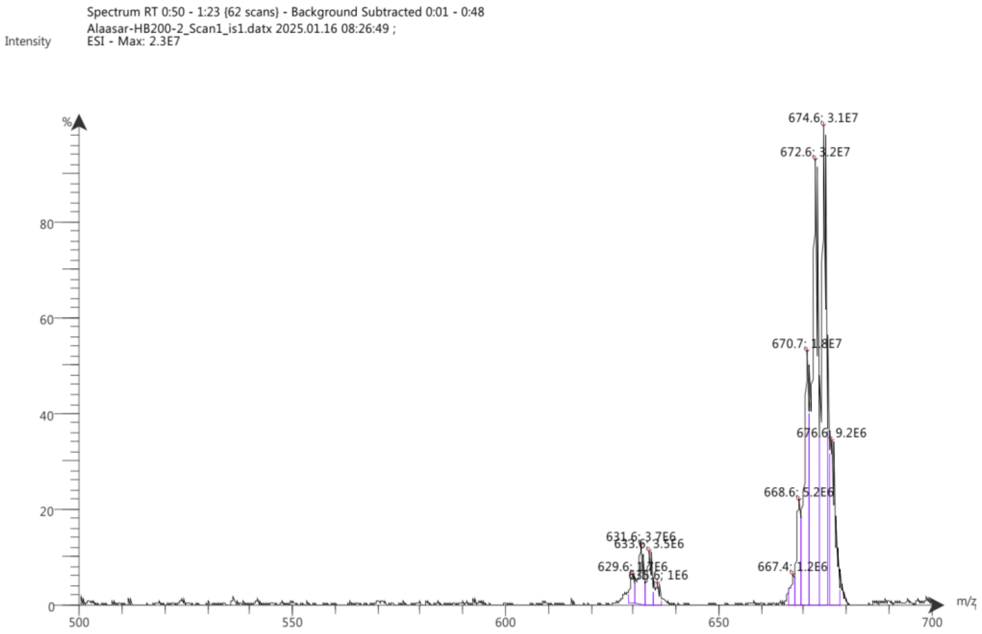
Spectrum RT 0:50 - 1:23 (62 scans) - Background Subtracted 0:01 - 0:48
Alaasar-HB200-2_Scan1_is1.datx 2025.01.16 08:26:49 ;
Intensity
ESI - Max: 2.3E7
674.6; 3.1E7
672.6; 3.2E7
670.7; 1.8E7
676.6; 9.2E6
668.6; 5.2E6
667.4; 1.2E6
m/z

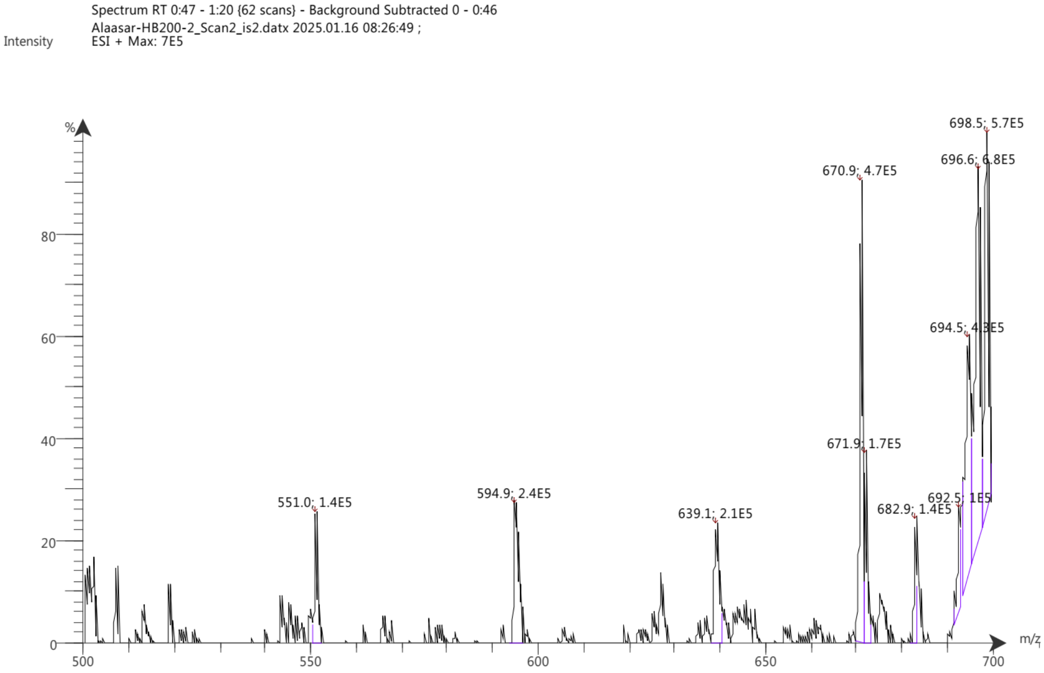
Spectrum RT 0:47 - 1:20 (62 scans) - Background Subtracted 0 - 0:46
Alaasar-HB200-2_Scan2_is2.datx 2025.01.16 08:26:49 ;
Intensity
ESI + Max: 7E5
698.5; 5.7E5
696.6; 6.8E5
670.9; 4.7E5
694.5; 4.3E5
671.9; 1.7E5
551.0; 1.4E5
594.9; 2.4E5
639.1; 2.1E5
682.9; 1.4E5
692.5; 1E5
m/z

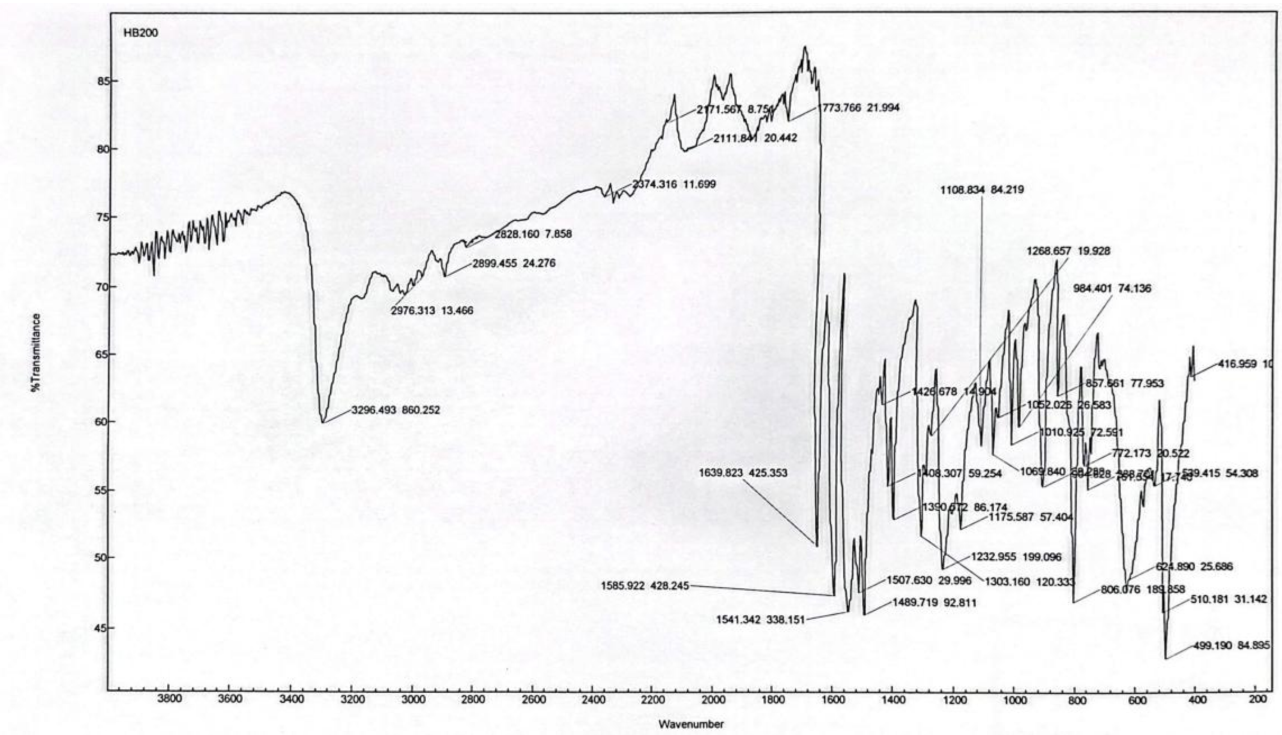
HB200
%Transmittance
Wavenumber
3296.493 860.252
2976.313 13.466
2899.455 24.276
2828.160 7.858
2374.316 11.699
2171.567 8.751
2111.841 20.442
1773.766 21.994
1639.823 425.353
1585.922 428.245
1541.342 338.151
1507.630 29.996
1489.719 92.811
1108.834 84.219
1268.657 19.928
984.401 74.136
1232.955 199.096
1303.160 120.333
1175.587 57.404
857.661 77.953
772.173 20.522
624.890 25.686
806.076 189.858
510.181 31.142
499.190 84.895
539.415 54.308

1,1'-(methylenebis(4,1-phenylene))bis(3-(4-(benzylselanyl)phenyl)urea) (HB201)

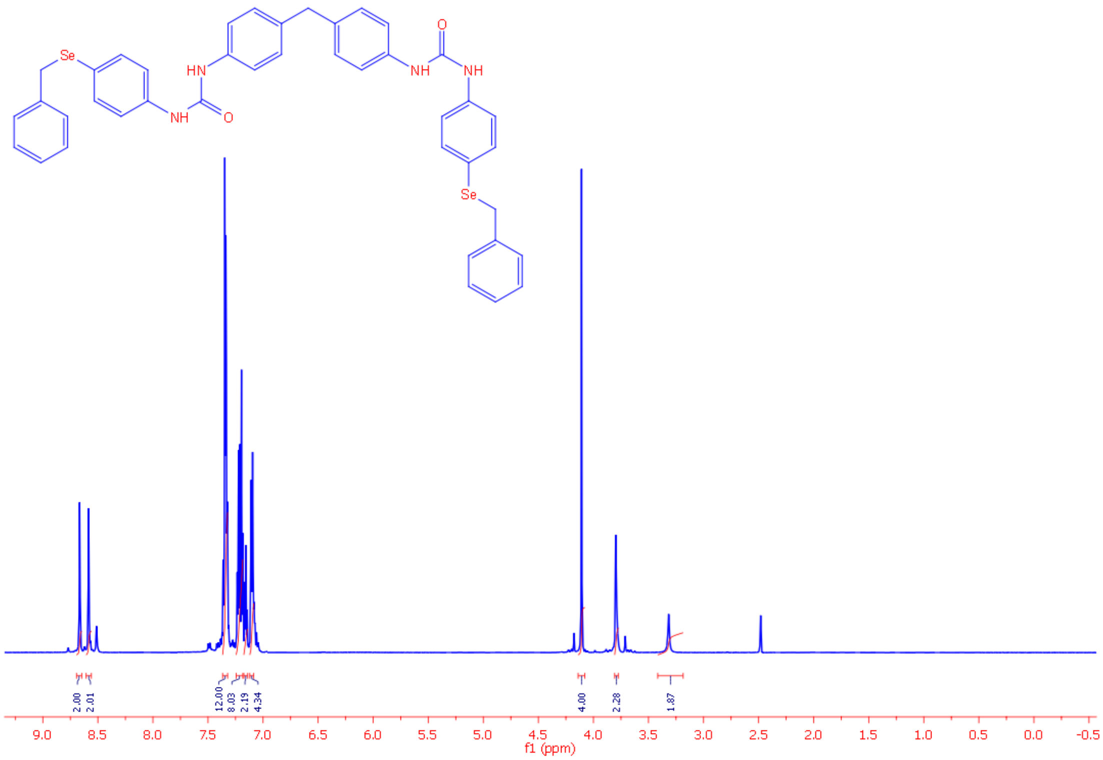

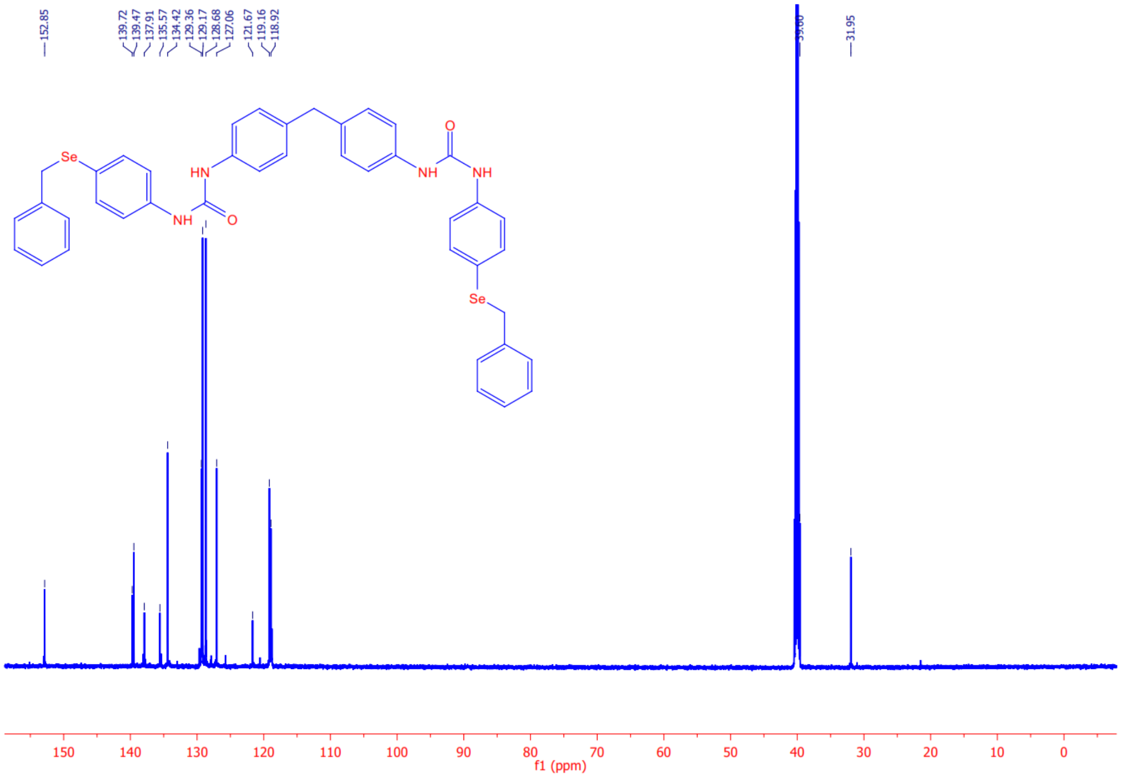

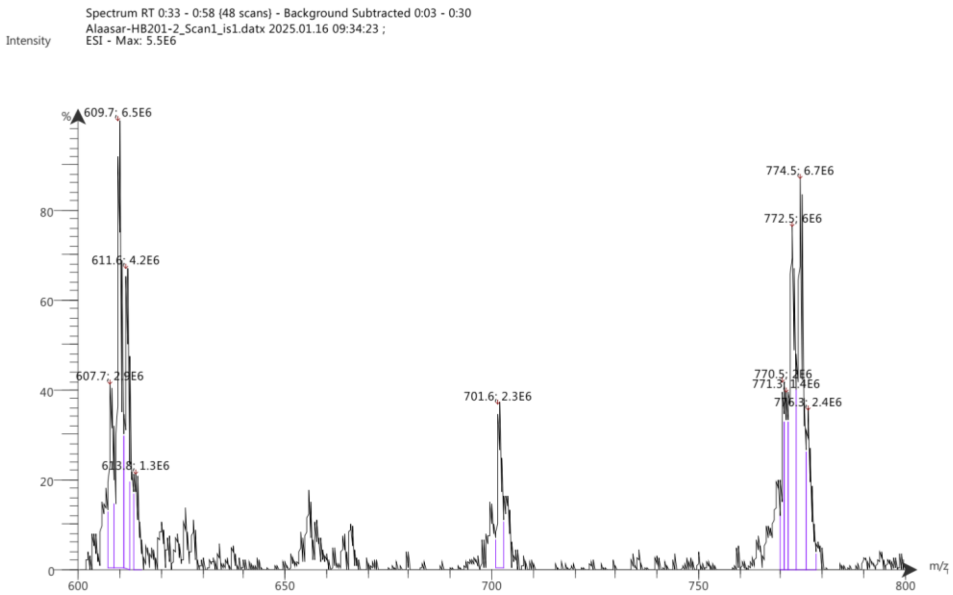

Spectrum RT 0:33 - 0:58 (48 scans) - Background Subtracted 0:03 - 0:30
Alaasar-HB201-2_Scan1_is1.datx 2025.01.16 09:34:23 ;
Intensity
ESI - Max: 5.5E6
609.7; 6.5E6
774.5; 6.7E6
772.5; 6E6
611.6; 4.2E6
607.7; 2.9E6
770.5; 2E6
771.3; 1.4E6
701.6; 2.3E6
776.3; 2.4E6
613.8; 1.3E6
m/z

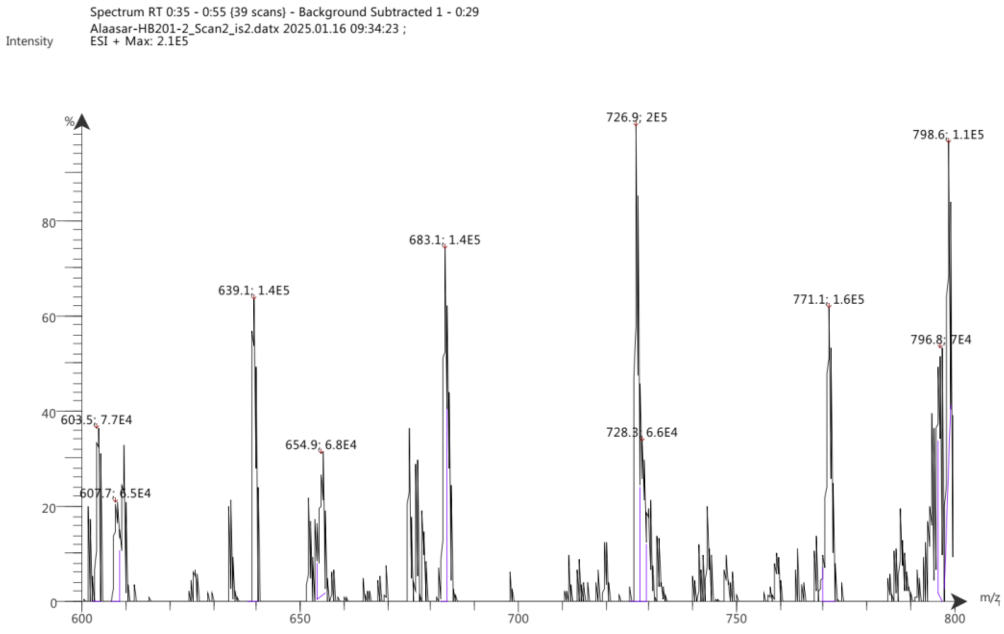

Spectrum RT 0:35 - 0:55 (39 scans) - Background Subtracted 1 - 0:29
Alaasar-HB201-2_Scan2_is2.datx 2025.01.16 09:34:23 ;
Intensity
ESI + Max: 2.1E5
726.9; 2E5
798.6; 1.1E5
683.1; 1.4E5
639.1; 1.4E5
771.1; 1.6E5
796.8; 7E4
603.5; 7.7E4
654.9; 6.8E4
728.3; 6.6E4
607.7; 6.5E4
m/z

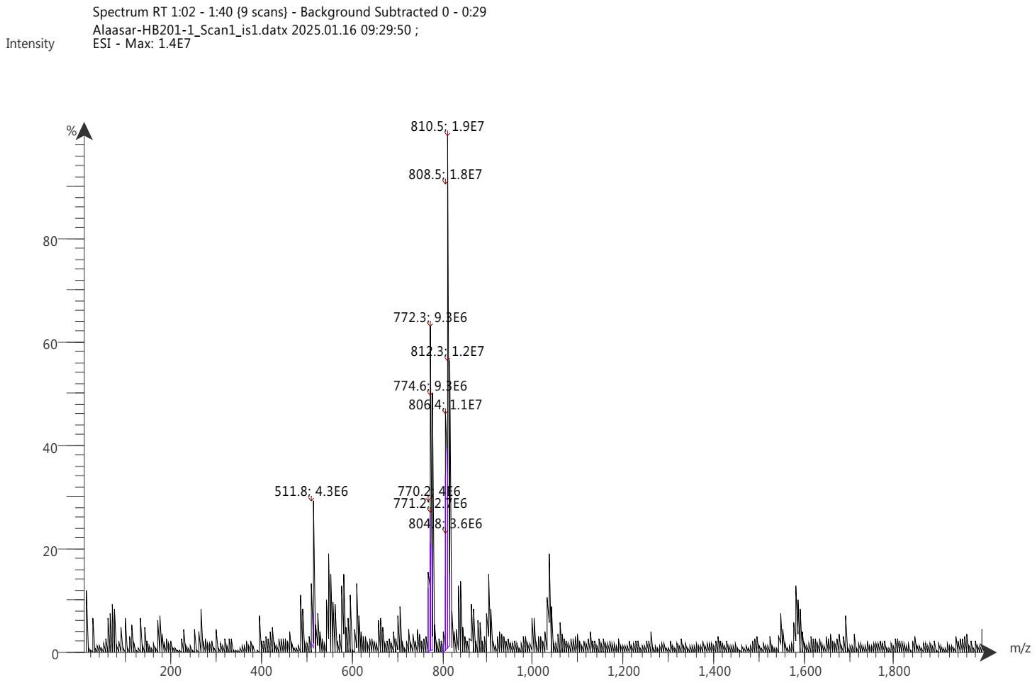

Spectrum RT 1:02 - 1:40 (9 scans) - Background Subtracted 0 - 0:29
Alaasar-HB201-1_Scan1_is1.datx 2025.01.16 09:29:50 ;
Intensity
ESI - Max: 1.4E7
%
810.5; 1.9E7
808.5; 1.8E7
772.3; 9.3E6
812.3; 1.2E7
774.6; 9.3E6
806.4; 1.1E7
511.8; 4.3E6
804.8; 3.6E6
m/z

1,1'-(hexane-1,6-diyl)bis(3-(4-(methylselanyl)phenyl)urea) (HB192)

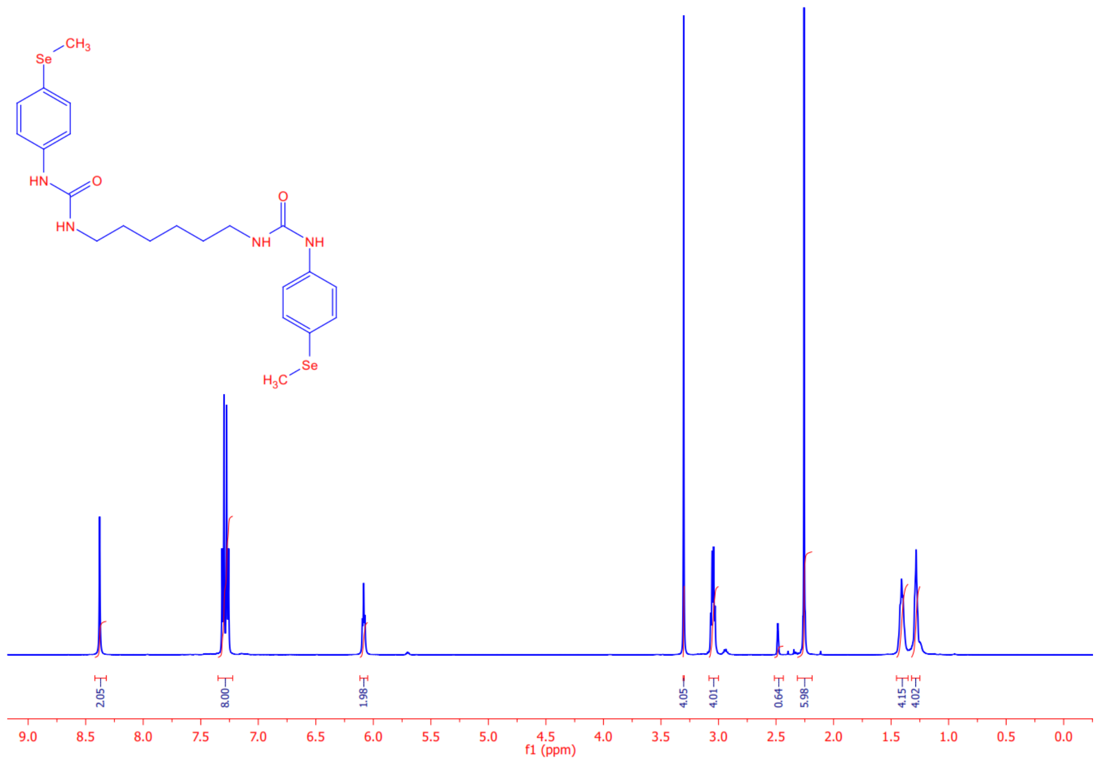

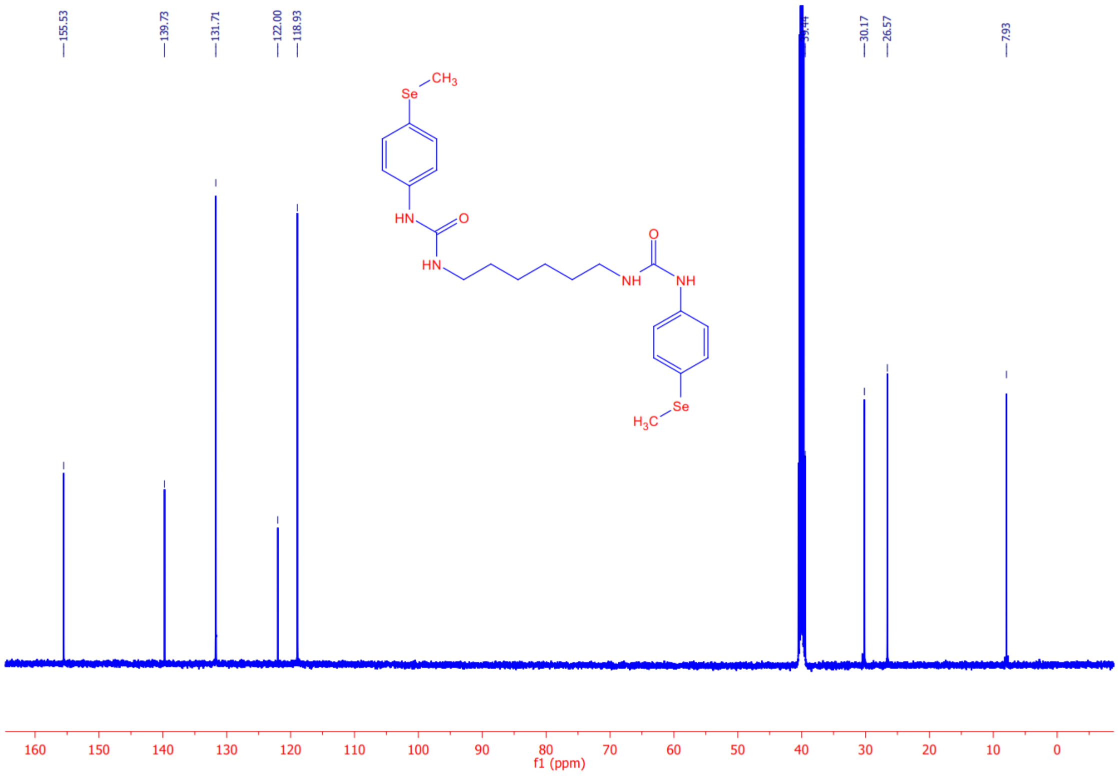

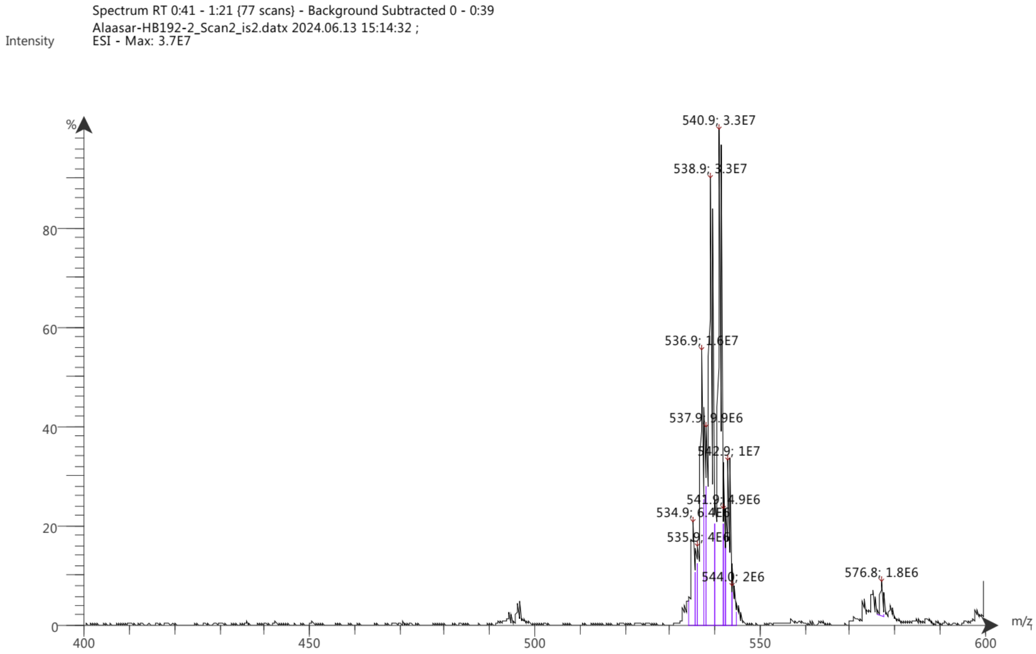
Spectrum RT 0:41 - 1:21 (77 scans) - Background Subtracted 0 - 0:39
Alaasar-HB192-2_Scan2_is2.datx 2024.06.13 15:14:32 ;
Intensity
ESI - Max: 3.7E7
540.9; 3.3E7
538.9; 3.3E7
536.9; 1.6E7
537.9; 9.9E6
542.9; 1E7
541.9; 4.9E6
534.9; 6.4E6
535.9; 4E6
544.0; 2E6
576.8; 1.8E6
%
m/z

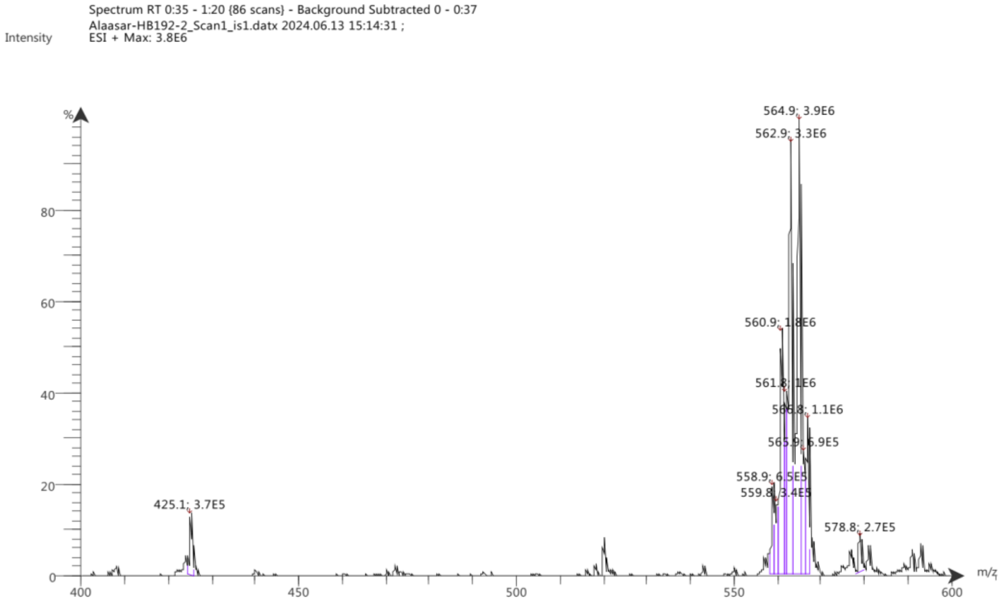
Spectrum RT 0:35 - 1:20 (86 scans) - Background Subtracted 0 - 0:37
Alaasar-HB192-2_Scan1_is1.datx 2024.06.13 15:14:31 ;
Intensity
ESI + Max: 3.8E6
564.9; 3.9E6
562.9; 3.3E6
560.9; 1.8E6
561.8; 1E6
566.8; 1.1E6
565.9; 6.9E5
558.9; 6.5E5
559.8; 3.4E5
425.1; 3.7E5
578.8; 2.7E5
%
m/z

1,1'-(hexane-1,6-diyl)bis(3-(4-(allylselanyl)phenyl)urea) (HB198)

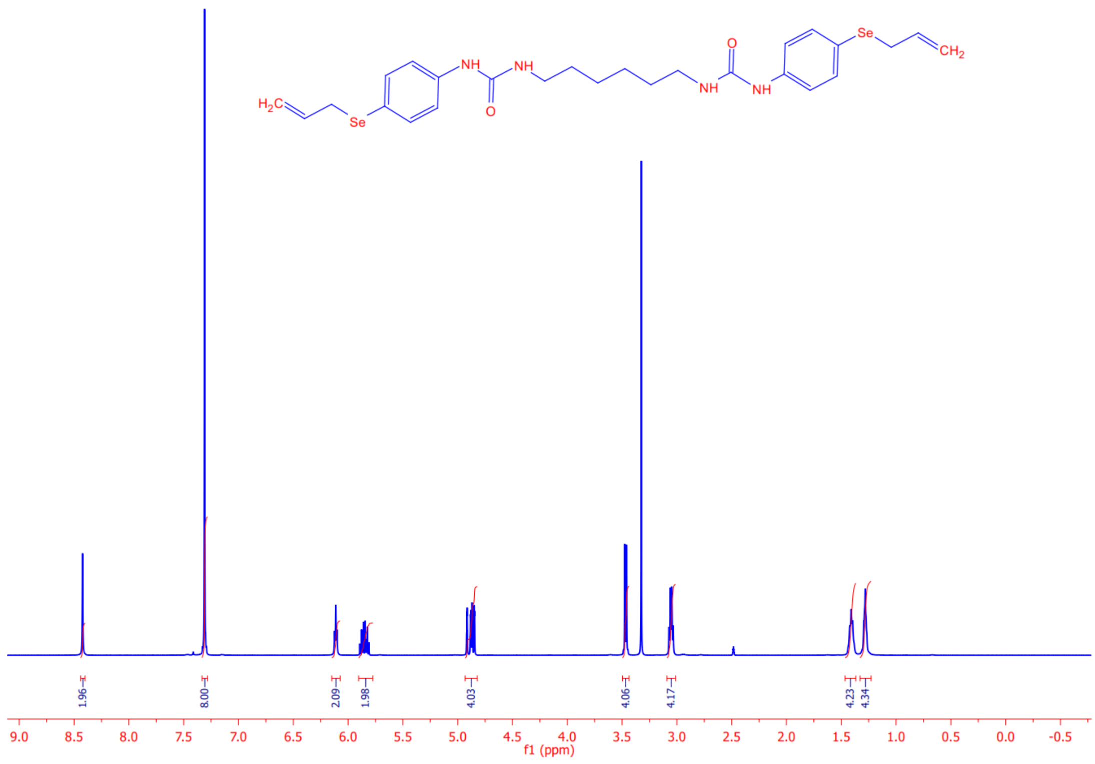

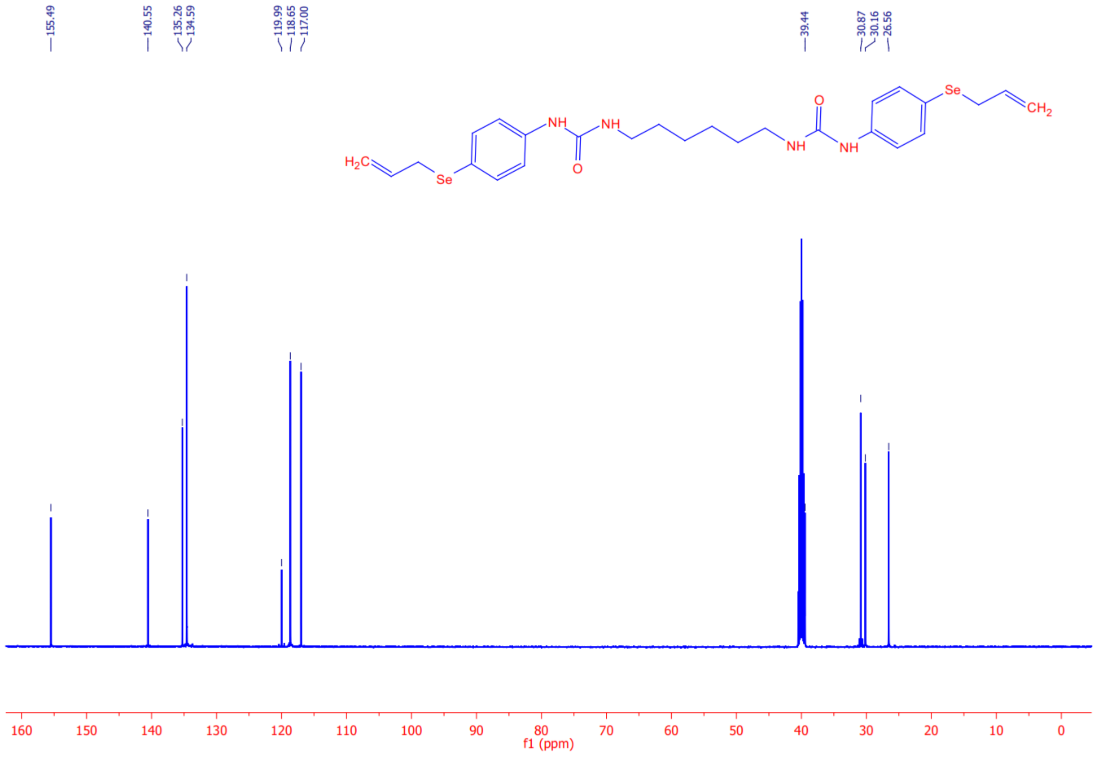

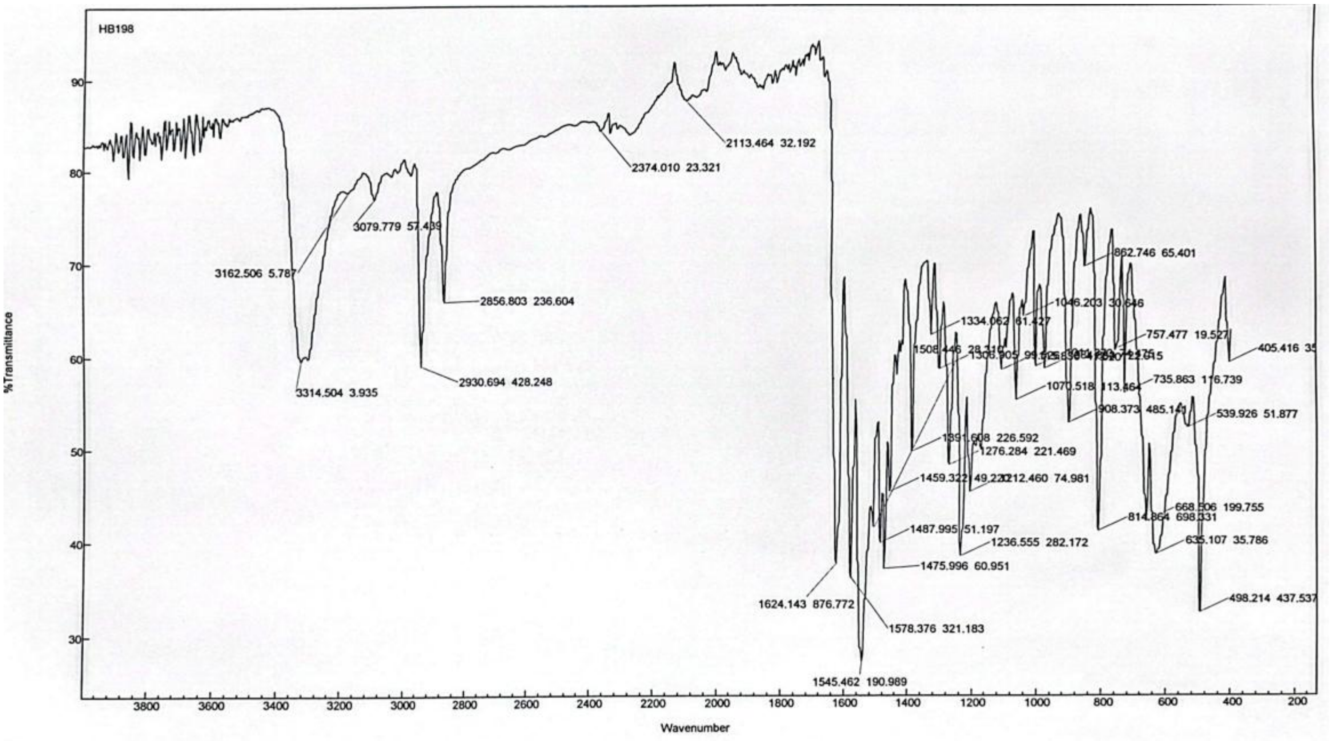
HB198
%Transmittance
Wavenumber
3314.504 3.935
3162.506 5.787
3079.779 57.439
2930.694 428.248
2856.803 236.604
2374.010 23.321
2113.464 32.192
1624.143 876.772
1578.376 321.183
1545.462 190.989
1475.996 60.951
1487.995 51.197
1236.555 282.172
1459.322 49.220
1212.460 74.981
1391.608 226.592
1276.284 221.469
1334.062 61.427
1046.203 30.646
862.746 65.401
757.477 19.527
735.863 116.739
908.373 485.141
539.926 51.877
405.416
814.864
668.506 199.755
698.331
635.107 35.786
498.214 437.537

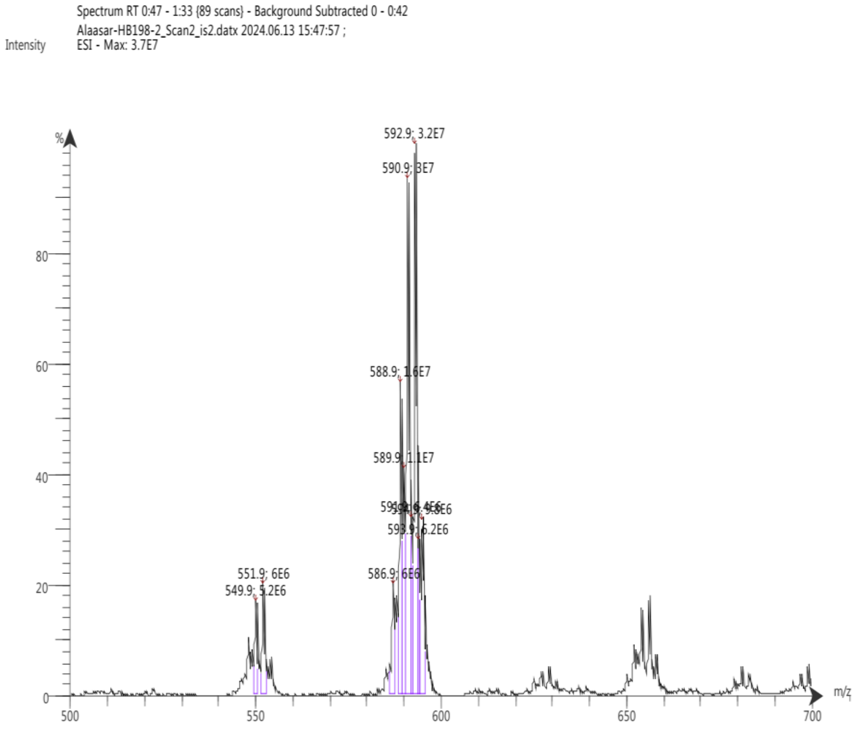
Spectrum RT 0:47 - 1:33 (89 scans) - Background Subtracted 0 - 0:42
Alaasar-HB198-2_Scan2_is2.datx 2024.06.13 15:47:57 ;
Intensity
ESI - Max: 3.7E7
592.9; 3.2E7
590.9; 3E7
588.9; 1.6E7
589.9; 1.1E7
593.9; 6.2E6
586.9; 6E6
551.9; 6E6
549.9; 5.2E6
m/z

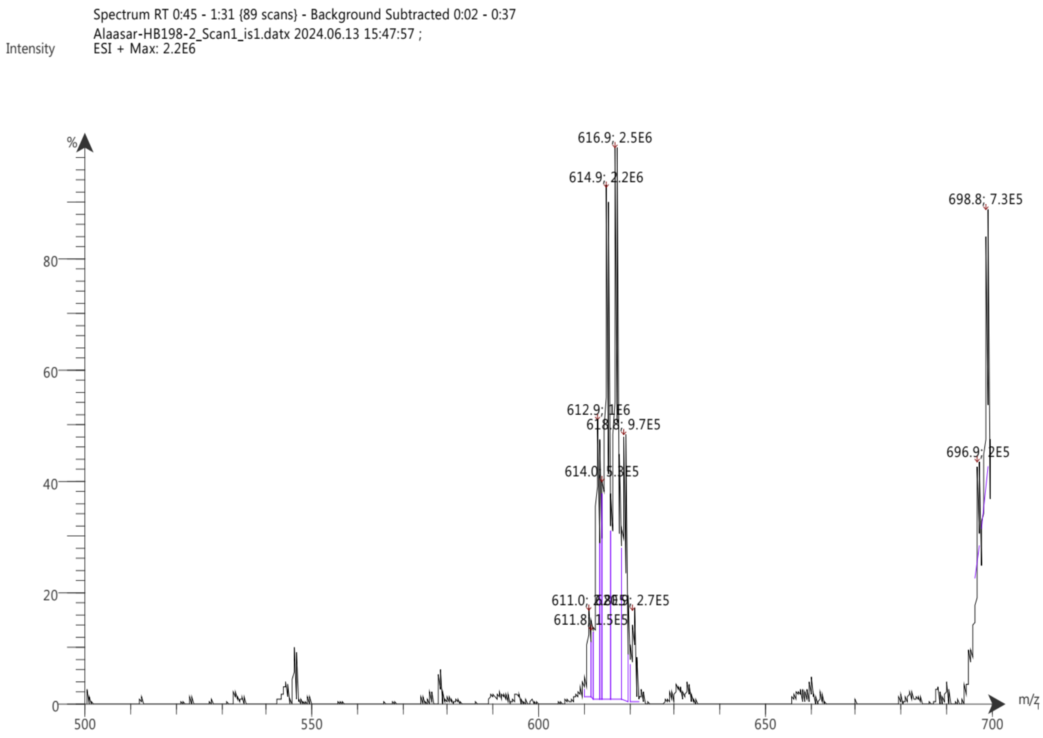

Spectrum RT 0:45 - 1:31 (89 scans) - Background Subtracted 0:02 - 0:37
Alaasar-HB198-2_Scan1_is1.datx 2024.06.13 15:47:57 ;
Intensity
ESI + Max: 2.2E6
%
616.9; 2.5E6
614.9; 2.2E6
698.8; 7.3E5
612.9; 1E6
618.8; 9.7E5
614.0; 5.3E5
696.9; 2E5
611.8; 1.5E5
m/z

1,1'-(hexane-1,6-diyl)bis(3-(4-(benzylselanyl)phenyl)urea) (HB203)

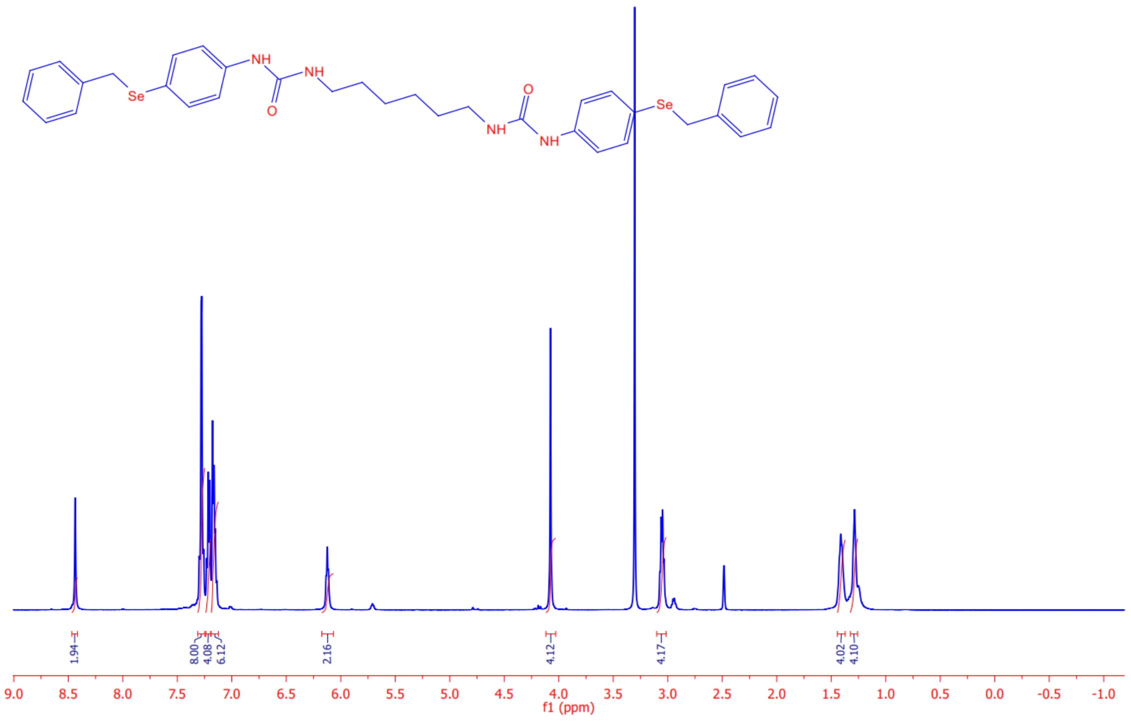

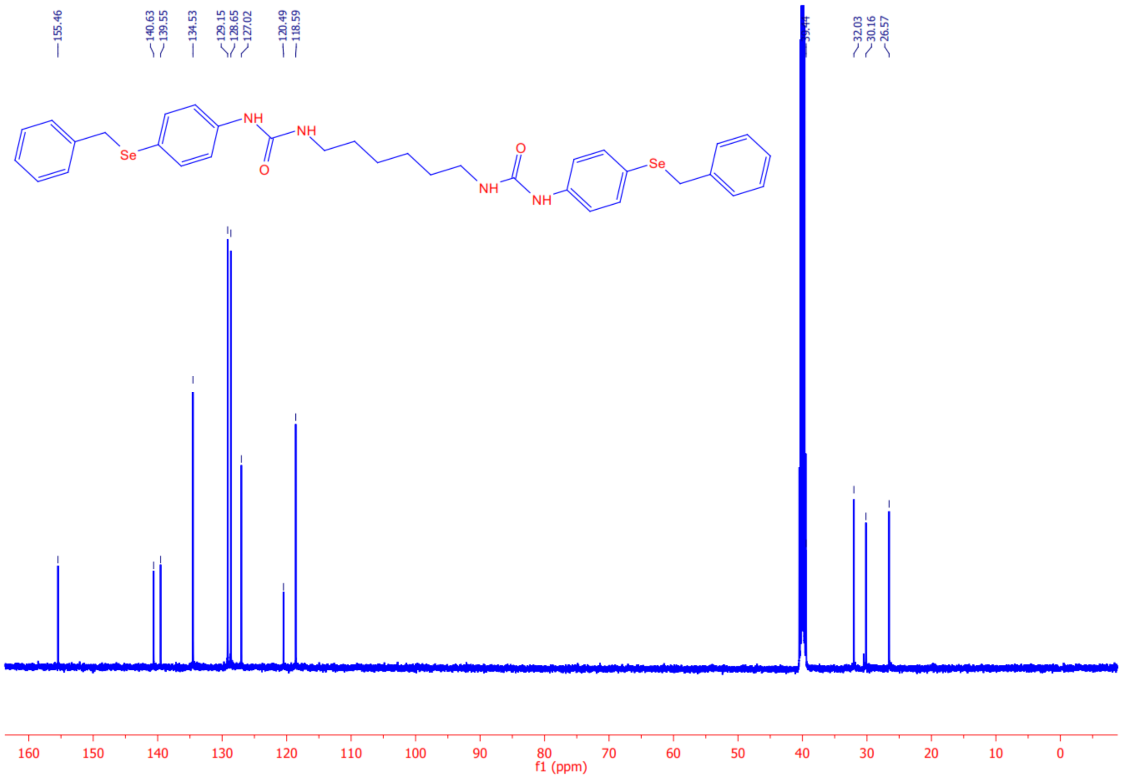

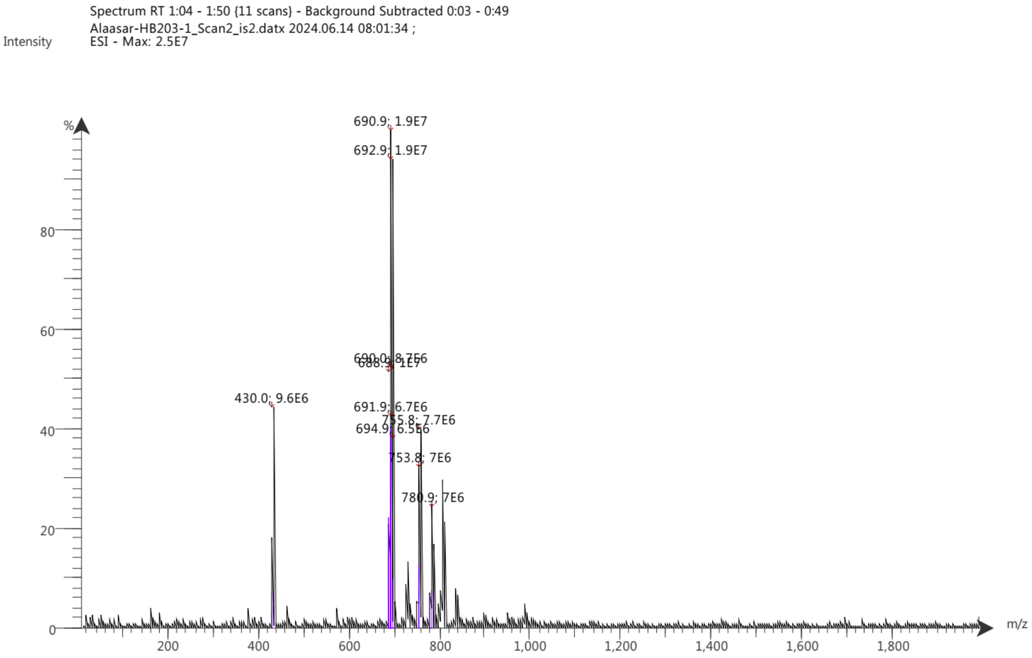
Spectrum RT 1:04 - 1:50 (11 scans) - Background Subtracted 0:03 - 0:49
Alaasar-HB203-1_Scan2_is2.datx 2024.06.14 08:01:34 ;
Intensity
ESI - Max: 2.5E7
690.9; 1.9E7
692.9; 1.9E7
430.0; 9.6E6
691.9; 6.7E6
755.8; 7.7E6
694.9; 6.5E6
753.8; 7E6
780.9; 7E6
m/z

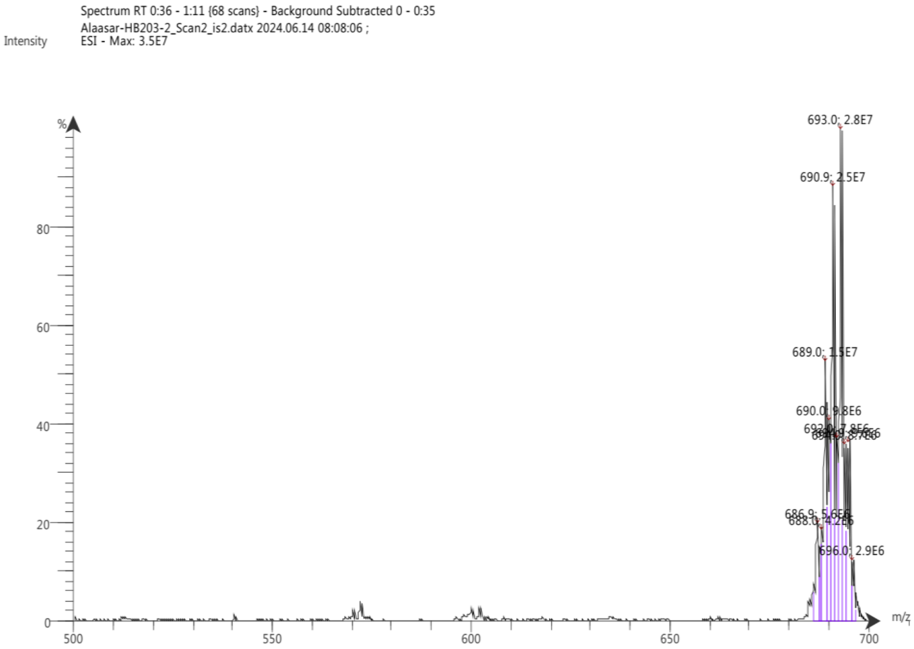
Spectrum RT 0:36 - 1:11 (68 scans) - Background Subtracted 0 - 0:35
Alaasar-HB203-2_Scan2_is2.datx 2024.06.14 08:08:06 ;
Intensity
ESI - Max: 3.5E7
693.0; 2.8E7
690.9; 2.5E7
689.0; 1.5E7
690.0; 9.8E6
696.0; 2.9E6

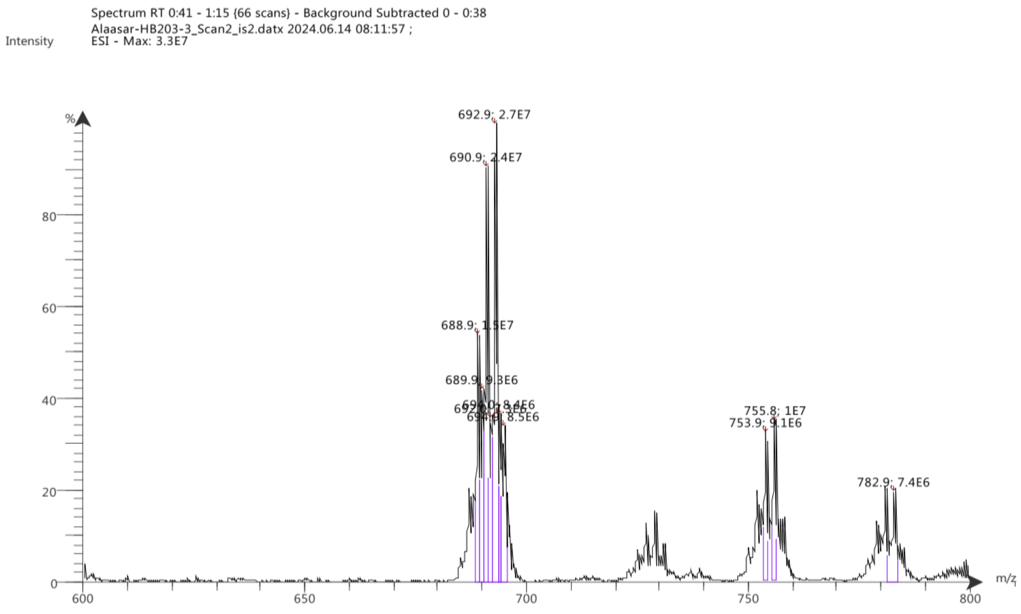
Spectrum RT 0:41 - 1:15 (66 scans) - Background Subtracted 0 - 0:38
Alaasar-HB203-3_Scan2_is2.datx 2024.06.14 08:11:57 ;
ESI - Max: 3.3E7
Intensity
692.9; 2.7E7
690.9; 2.4E7
688.9; 1.5E7
689.9; 9.3E6
694.9; 8.5E6
755.8; 1E7
753.9; 9.1E6
782.9; 7.4E6
m/z

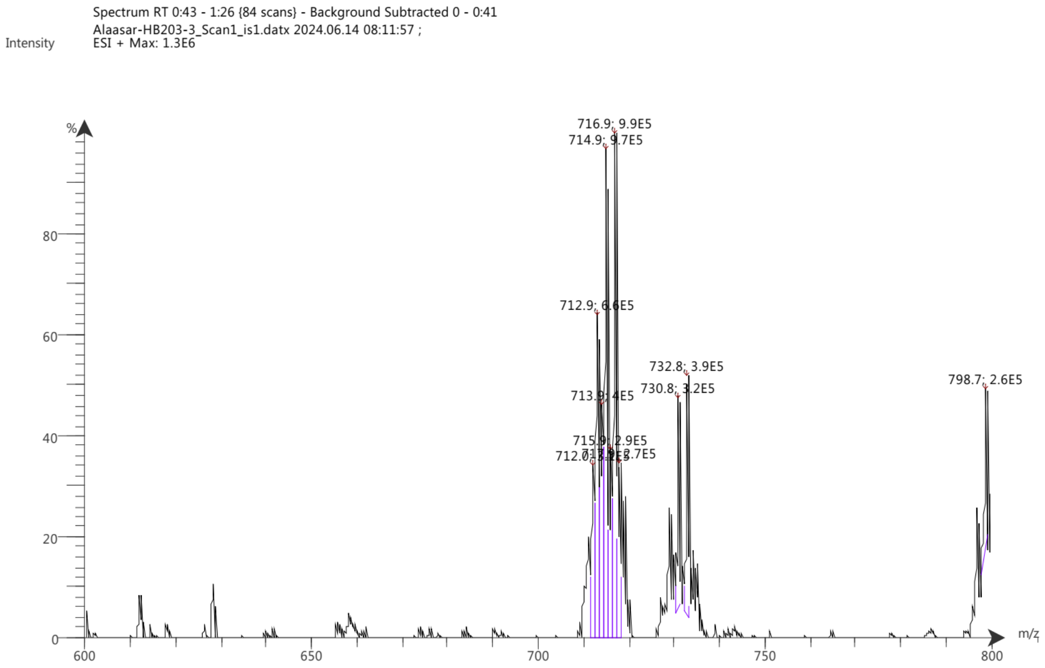
Spectrum RT 0:43 - 1:26 (84 scans) - Background Subtracted 0 - 0:41
Alaasar-HB203-3_Scan1_is1.datx 2024.06.14 08:11:57 ;
ESI + Max: 1.3E6
Intensity
716.9; 9.9E5
714.9; 9.7E5
712.9; 6.6E5
732.8; 3.9E5
730.8; 3.2E5
715.9; 2.9E5
798.7; 2.6E5
m/z

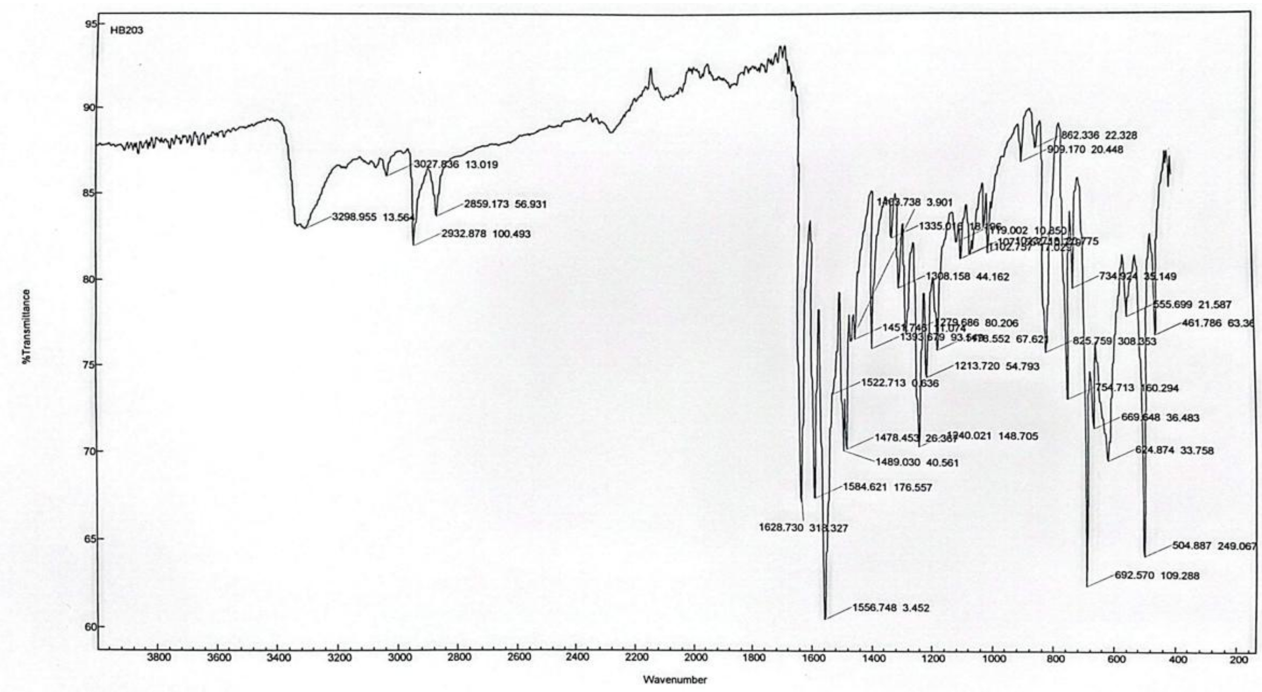
HB203
%Transmittance
Wavenumber
3298.955 13.564
3027.836 13.019
2932.878 100.493
2859.173 56.931
1628.730 318.327
1584.621 176.557
1556.748 3.452
1522.713 0.636
1489.030 40.561
1308.158 44.162
1240.021 148.705
1213.720 54.793
1279.686 80.206
909.170 20.448
862.336 22.328
825.759 308.353
754.713 160.294
734.924 35.149
692.570 109.288
669.648 36.483
624.874 33.758
555.699 21.587
504.887 249.067

1-(4-(methylselanyl)phenyl)-3-((1,3,3-trimethyl-5-(3-(4-(methylselanyl)phenyl)ureido)cyclohexyl)methyl)urea (HB188)

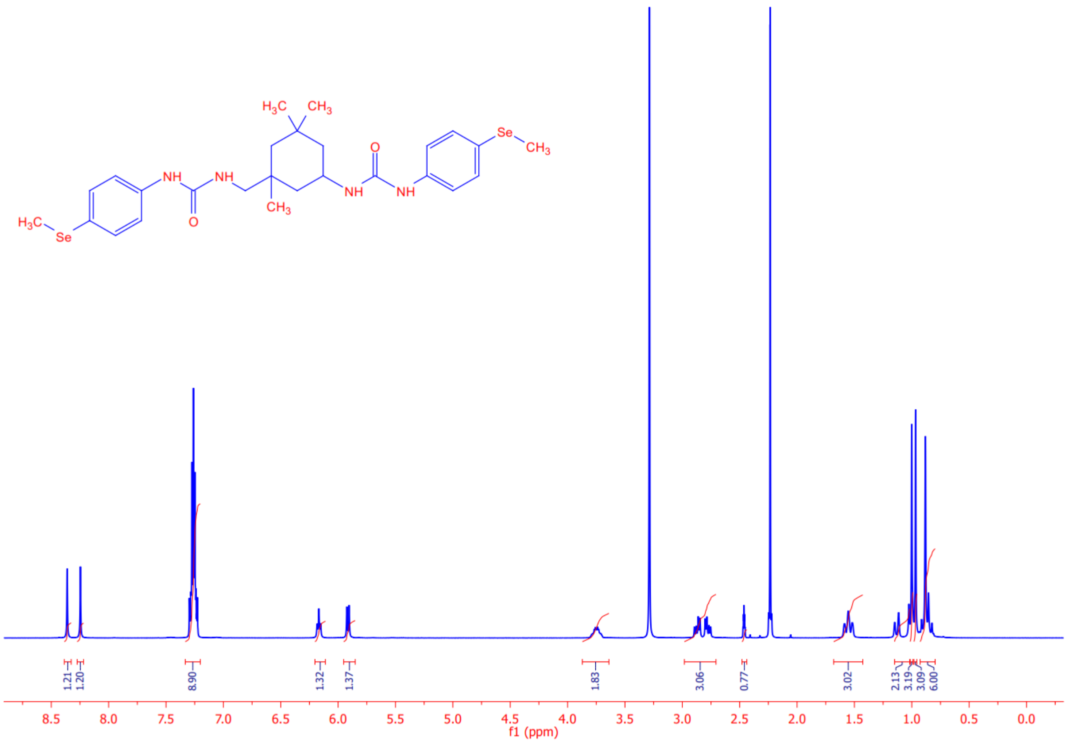

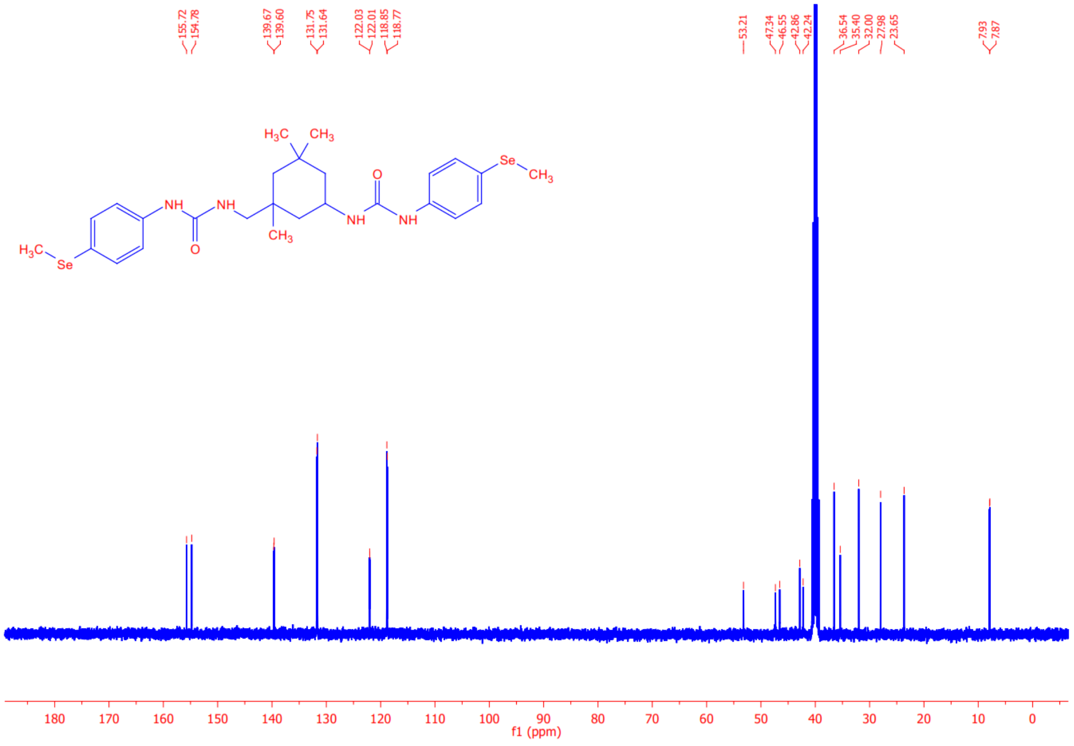

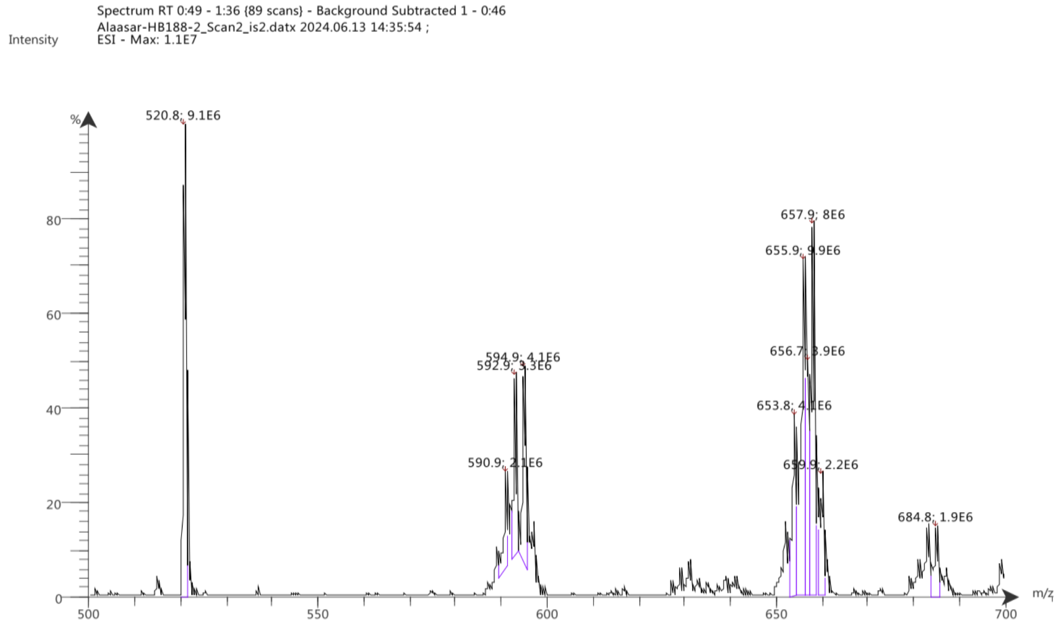
Spectrum RT 0:49 - 1:36 (89 scans) - Background Subtracted 1 - 0:46
Alaasar-HB188-2_Scan2_is2.datx 2024.06.13 14:35:54 ;
Intensity
ESI - Max: 1.1E7
520.8; 9.1E6
657.9; 8E6
655.9; 9.9E6
594.9; 4.1E6
592.9; 3.3E6
656.7; 3.9E6
653.8; 4.1E6
590.9; 2.1E6
659.9; 2.2E6
684.8; 1.9E6
%
m/z

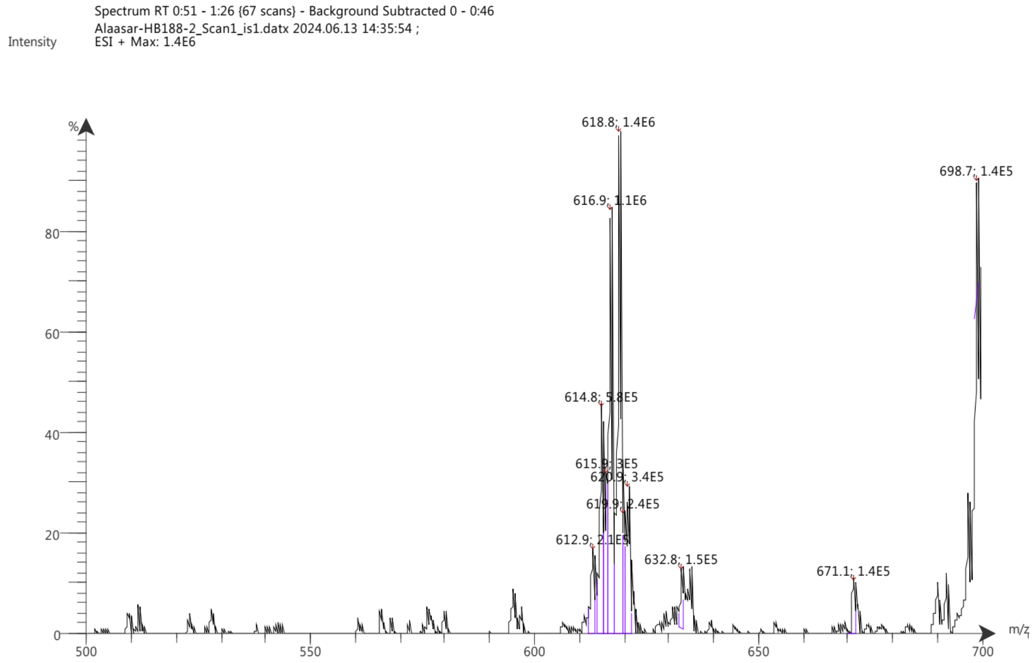
Spectrum RT 0:51 - 1:26 (67 scans) - Background Subtracted 0 - 0:46
Alaasar-HB188-2_Scan1_is1.datx 2024.06.13 14:35:54 ;
Intensity
ESI + Max: 1.4E6
618.8; 1.4E6
616.9; 1.1E6
698.7; 1.4E5
614.8; 5.8E5
615.9; 3E5
620.9; 3.4E5
619.9; 2.4E5
612.9; 2.1E5
632.8; 1.5E5
671.1; 1.4E5
%
m/z

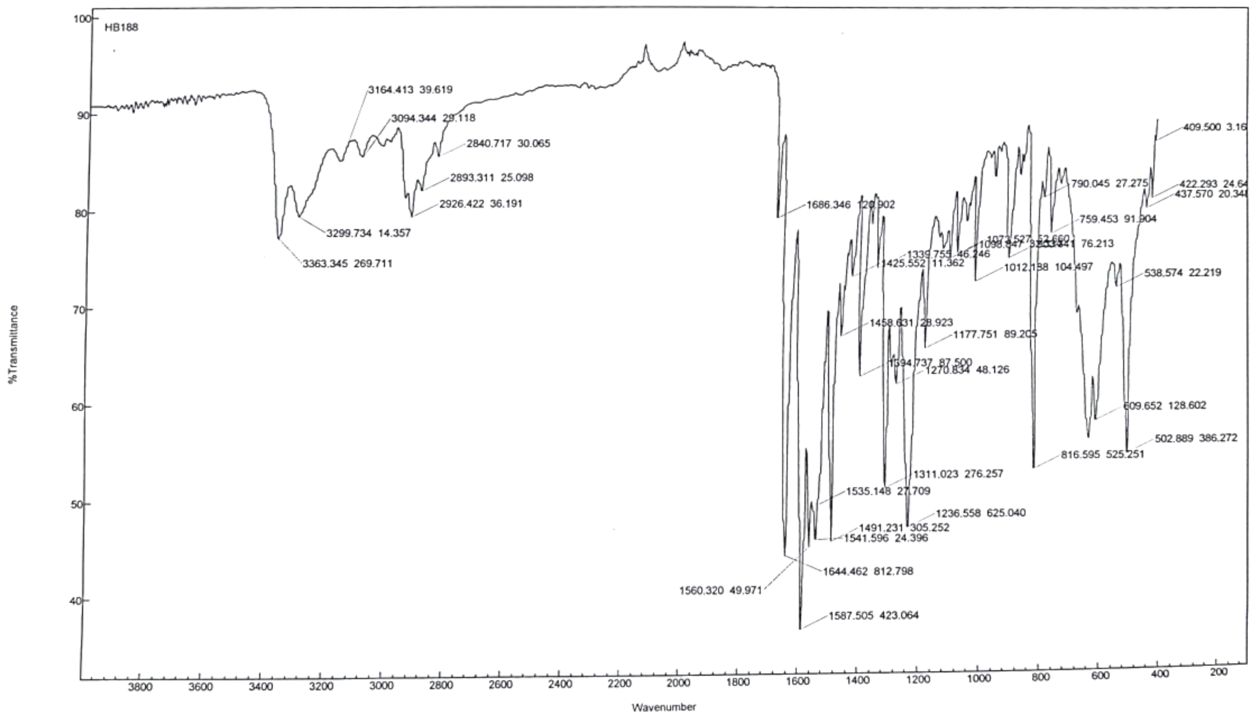
HB188
%Transmittance
Wavenumber
100
90
80
70
60
50
40
3800
3600
3400
3200
3000
2800
2600
2400
2200
2000
1800
1600
1400
1200
1000
800
600
400
200
3164.413 39.619
3094.344 29.118
2840.717 30.065
2893.311 25.098
2926.422 36.191
3299.734 14.357
3363.345 269.711
1686.346 120.902
1339.755 46.246
1425.552 11.362
1073.527 52.660
1012.138 104.497
790.045 27.275
759.453 91.904
409.500 3.16
422.293 24.64
437.570 20.34
538.574 22.219
1458.631 28.923
1177.751 89.205
1394.737 87.500
1270.834 48.126
609.652 128.602
502.889 386.272
816.595 525.251
1311.023 276.257
1535.148 27.709
1236.558 625.040
1491.231 305.252
1541.596 24.396
1644.462 812.798
1560.320 49.971
1587.505 423.064

1-(4-(allylselanyl)phenyl)-3-((5-(3-(4-(allylselanyl)phenyl)ureido)-1,3,3-trimethylcyclohexyl)methyl)urea (HB199)

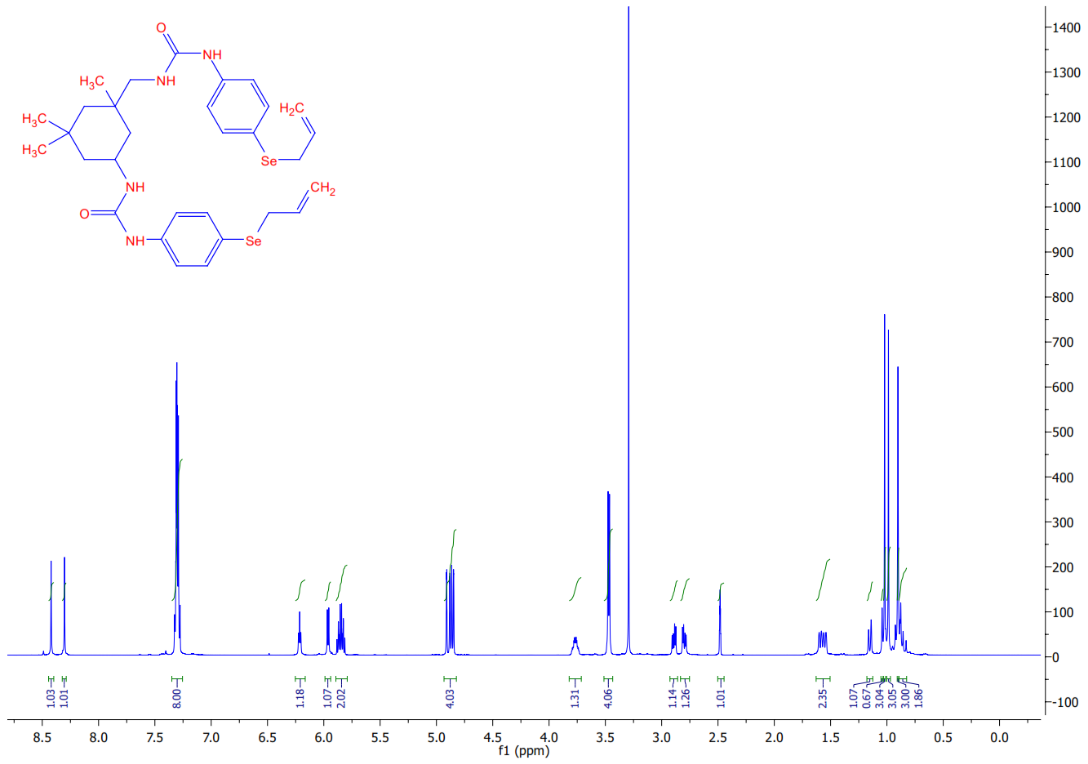

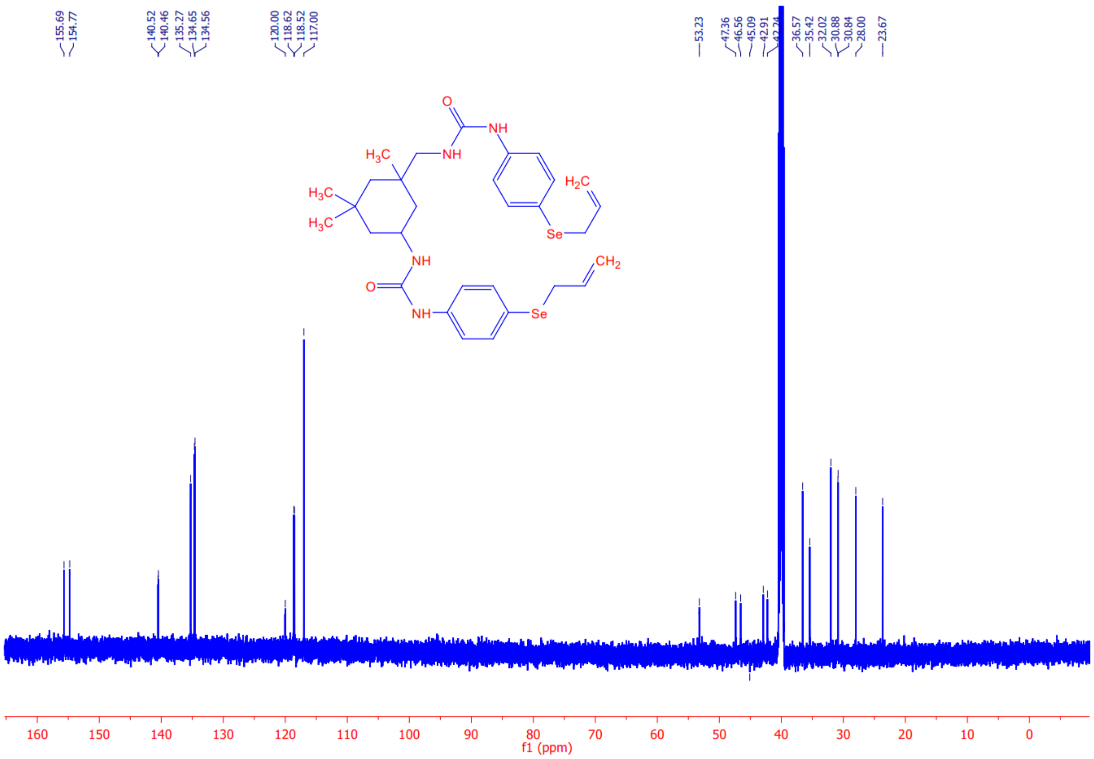

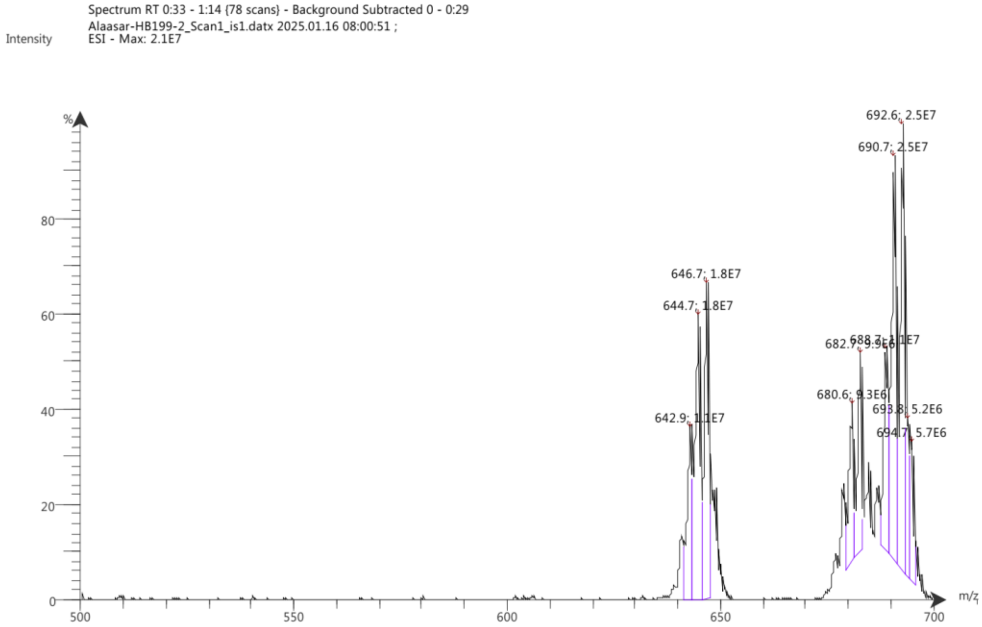

Spectrum RT 0:33 - 1:14 (78 scans) - Background Subtracted 0 - 0:29
Alaasar-HB199-2_Scan1_is1.datx 2025.01.16 08:00:51 ;
Intensity
ESI - Max: 2.1E7
%
692.6; 2.5E7
690.7; 2.5E7
646.7; 1.8E7
644.7; 1.8E7
680.6; 9.3E6
693.8; 5.2E6
694.7; 5.7E6
642.9; 1.1E7
80
60
40
20
0
500
550
600
650
700
m/z

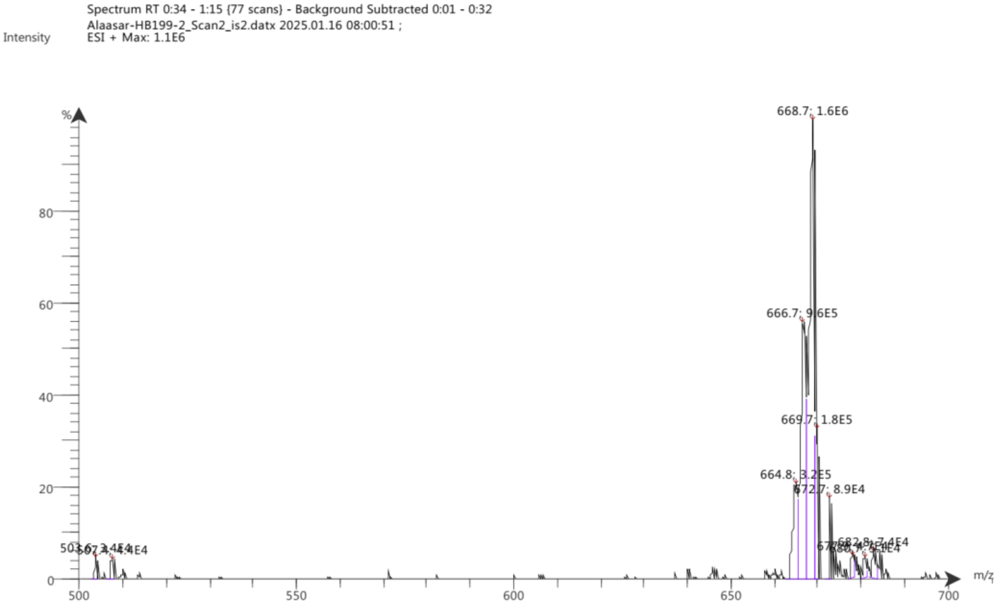

Spectrum RT 0:34 - 1:15 (77 scans) - Background Subtracted 0:01 - 0:32
Alaasar-HB199-2_Scan2_is2.datx 2025.01.16 08:00:51 ;
Intensity
ESI + Max: 1.1E6
%
668.7; 1.6E6
666.7; 9.6E5
669.7; 1.8E5
664.8; 3.2E5
672.7; 8.9E4
80
60
40
20
0
500
550
600
650
700
m/z

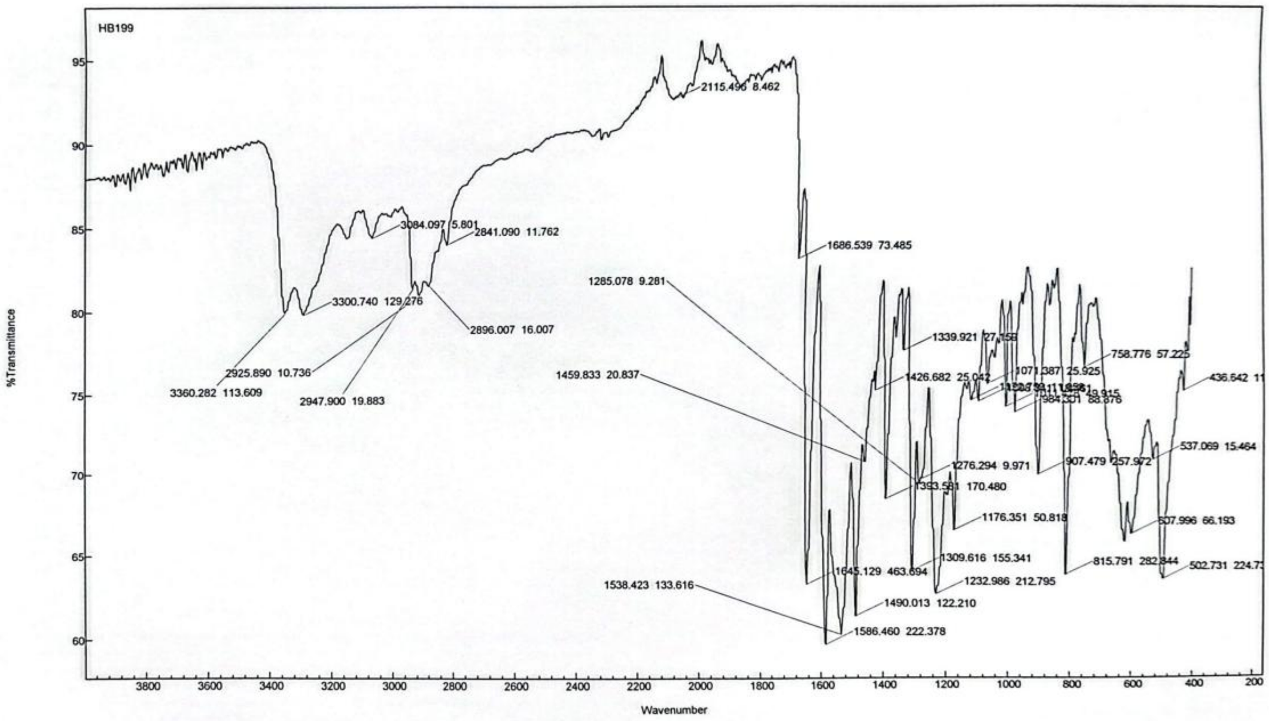
HB199
95
90
85
80
75
70
65
60
%Transmittance
3800
3600
3400
3200
3000
2800
2600
2400
2200
2000
1800
1600
1400
1200
1000
800
600
400
200
Wavenumber
2115.496 8.462
3084.097 5.801
2841.090 11.762
3300.740 129.276
2896.007 16.007
2925.890 10.736
3360.282 113.609
2947.900 19.883
1686.539 73.485
1285.078 9.281
1339.921 27.158
1459.833 20.837
1426.682 25.042
1071.387 25.925
758.776 57.225
436.642
984.301 88.876
1276.294 9.971
1393.581 170.480
907.479 257.972
537.069 15.464
1176.351 50.818
507.996 66.193
1309.616 155.341
1645.129 463.694
815.791 282.844
1232.986 212.795
1538.423 133.616
1490.013 122.210
1586.460 222.378

1-(4-(benzylselanyl)phenyl)-3-((5-(3-(4-(benzylselanyl)phenyl)ureido)-1,3,3-trimethylcyclohexyl)methyl)urea (HB206)

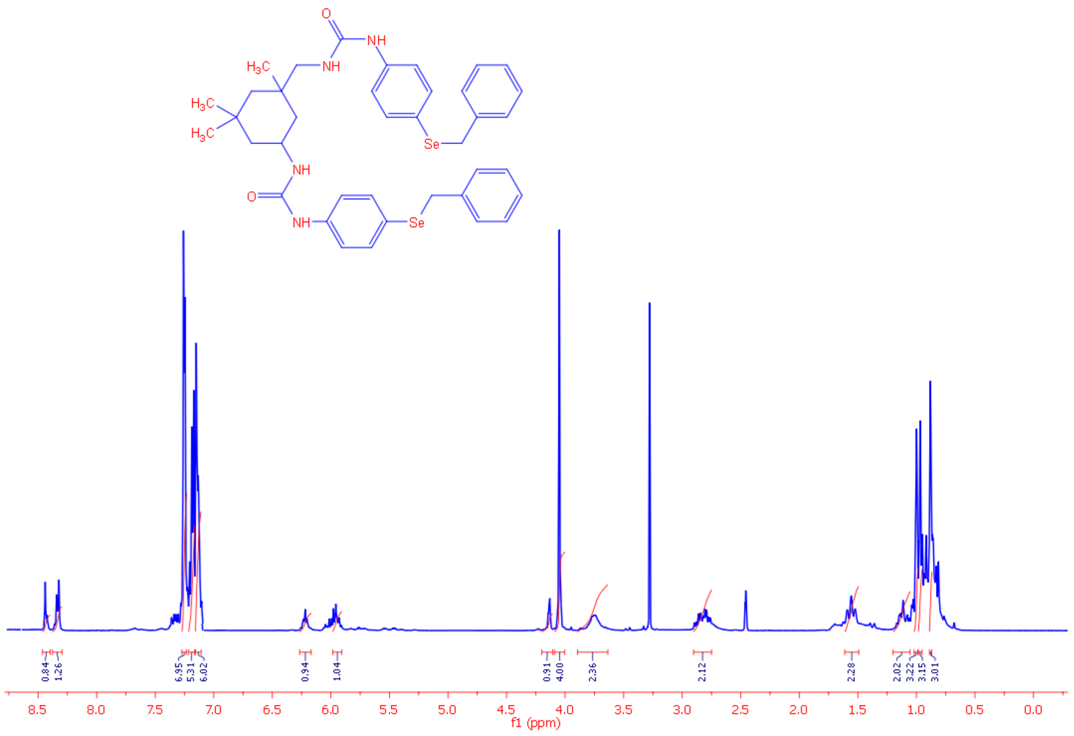

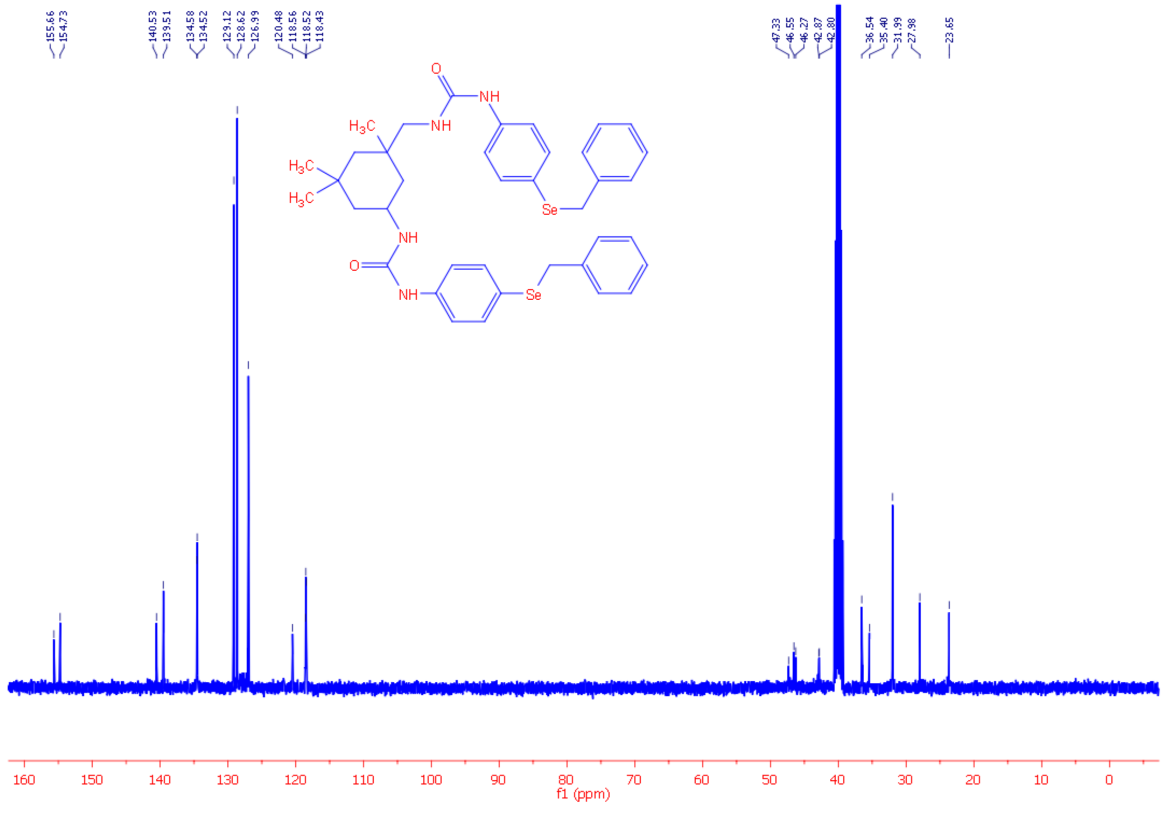

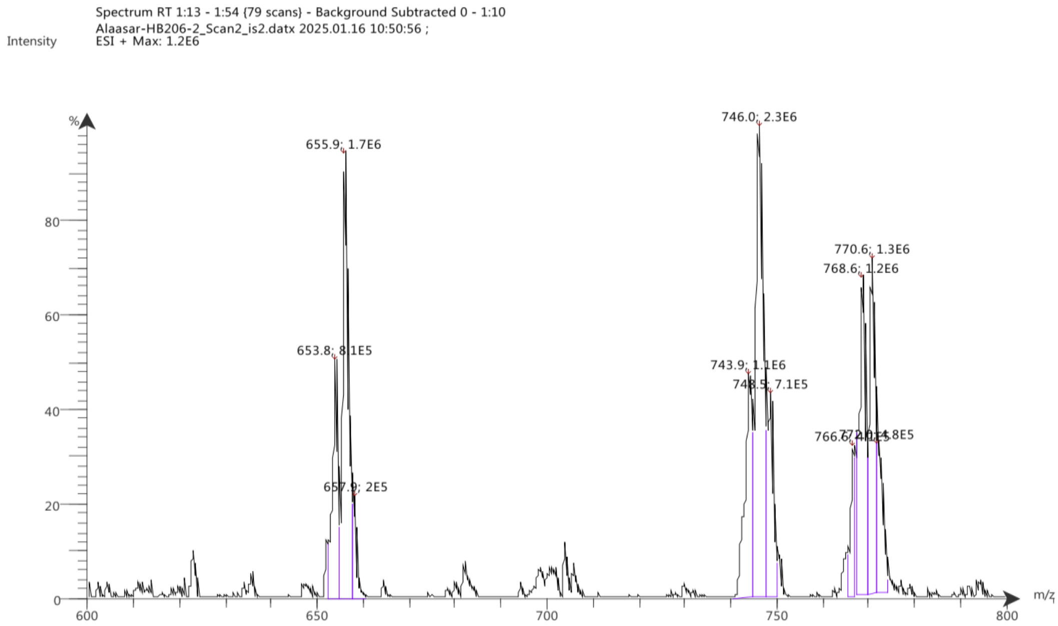
Spectrum RT 1:13 - 1:54 (79 scans) - Background Subtracted 0 - 1:10
Alaasar-HB206-2_Scan2_is2.datx 2025.01.16 10:50:56 ;
Intensity
ESI + Max: 1.2E6
%
655.9; 1.7E6
653.8; 8.1E5
657.9; 2E5
746.0; 2.3E6
743.9; 1.1E6
748.5; 7.1E5
770.6; 1.3E6
768.6; 1.2E6
m/z

1-(3-isocyanato-4-methylphenyl)-3-(4-(methylselanyl)phenyl)urea (HB191)

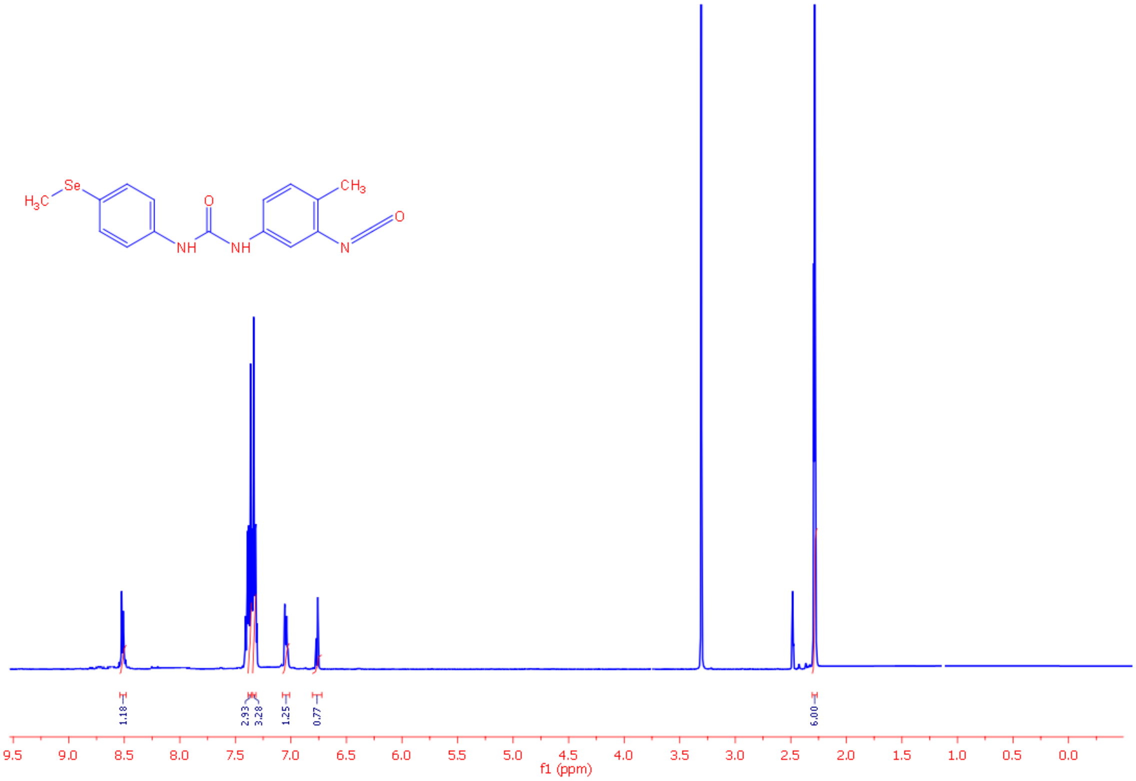

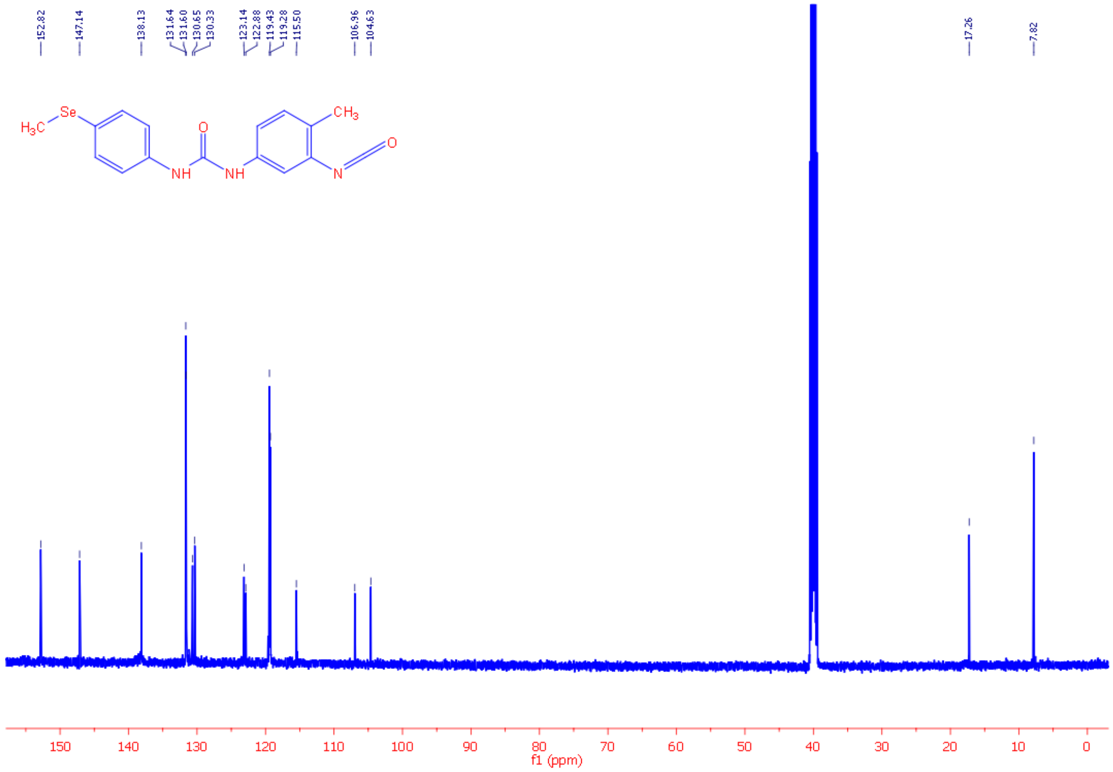

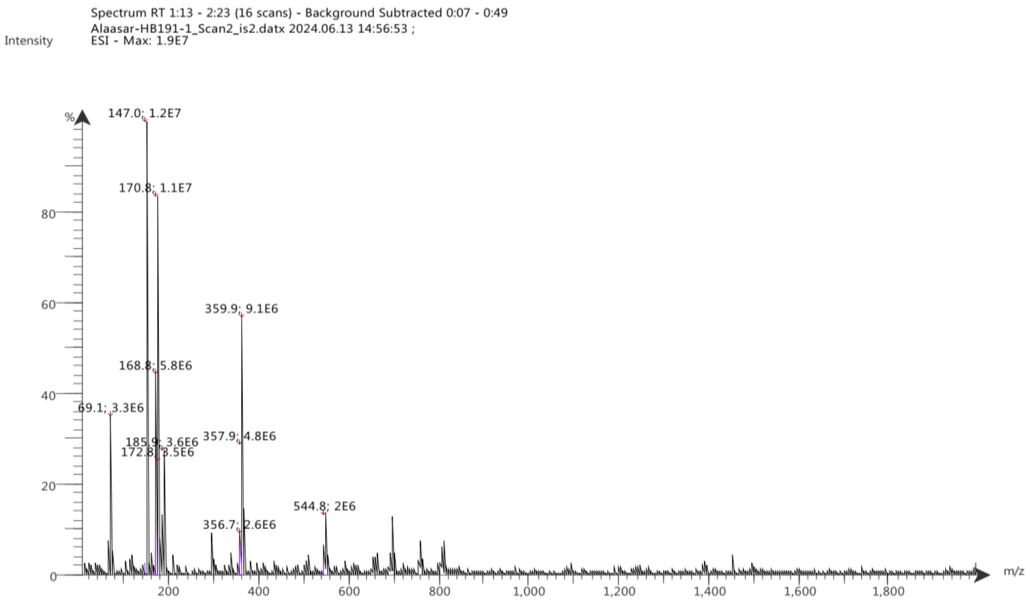

Spectrum RT 1:13 - 2:23 (16 scans) - Background Subtracted 0:07 - 0:49
Alaasar-HB191-1_Scan2_is2.datx 2024.06.13 14:56:53 ;
ESI - Max: 1.9E7
Intensity
%
147.0; 1.2E7
170.8; 1.1E7
359.9; 9.1E6
168.8; 5.8E6
69.1; 3.3E6
185.9; 3.6E6
172.8; 3.5E6
357.9; 4.8E6
544.8; 2E6
356.7; 2.6E6
m/z

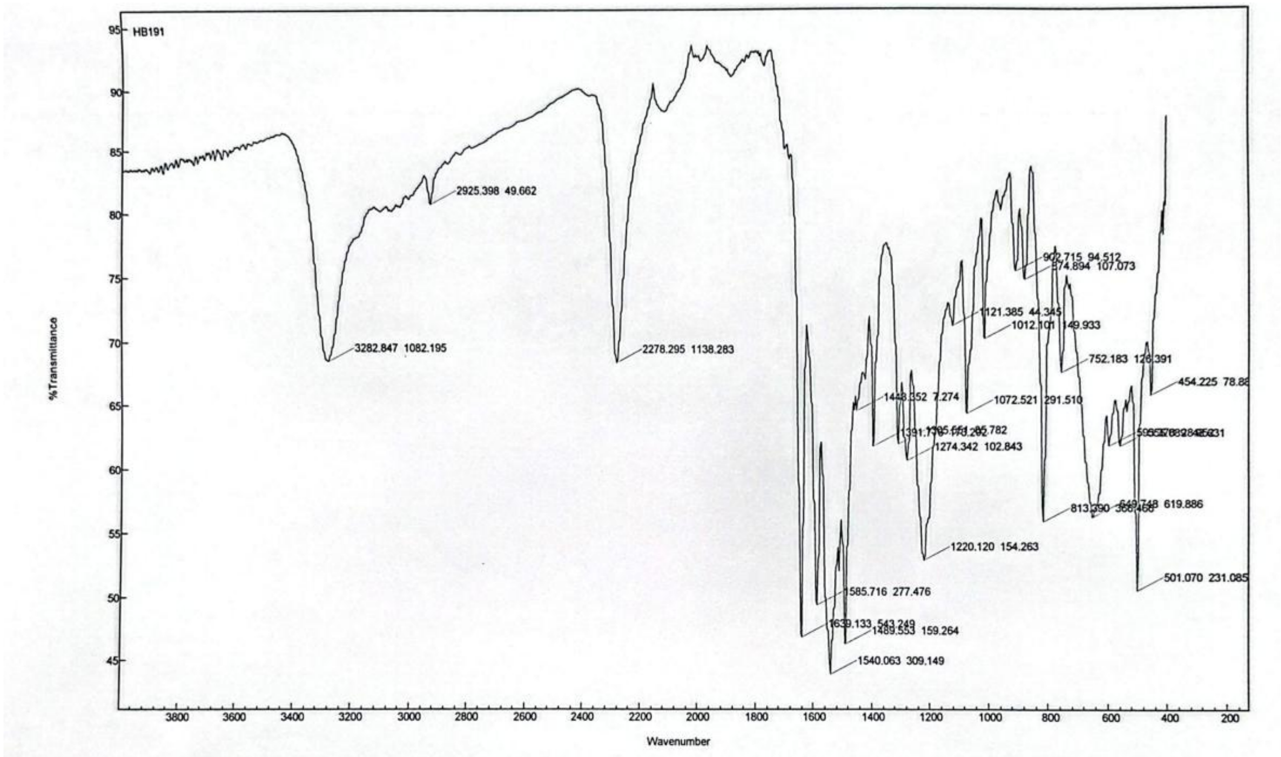

HB191
%Transmittance
Wavenumber
3282.847 1082.195
2925.398 49.662
2278.295 1138.283
1585.716 277.476
1639.133 543.249
1489.553 159.264
1540.063 309.149
1448.352 7.274
1274.342 102.843
1220.120 154.263
1121.385 44.345
1012.101 149.933
1072.521 291.510
902.715 94.512
874.894 107.073
752.183 126.391
813.390
649.748 619.886
454.225
501.070 231.085

1-(4-(allylselanyl)phenyl)-3-(3-isocyanato-4-methylphenyl)urea (HB204)

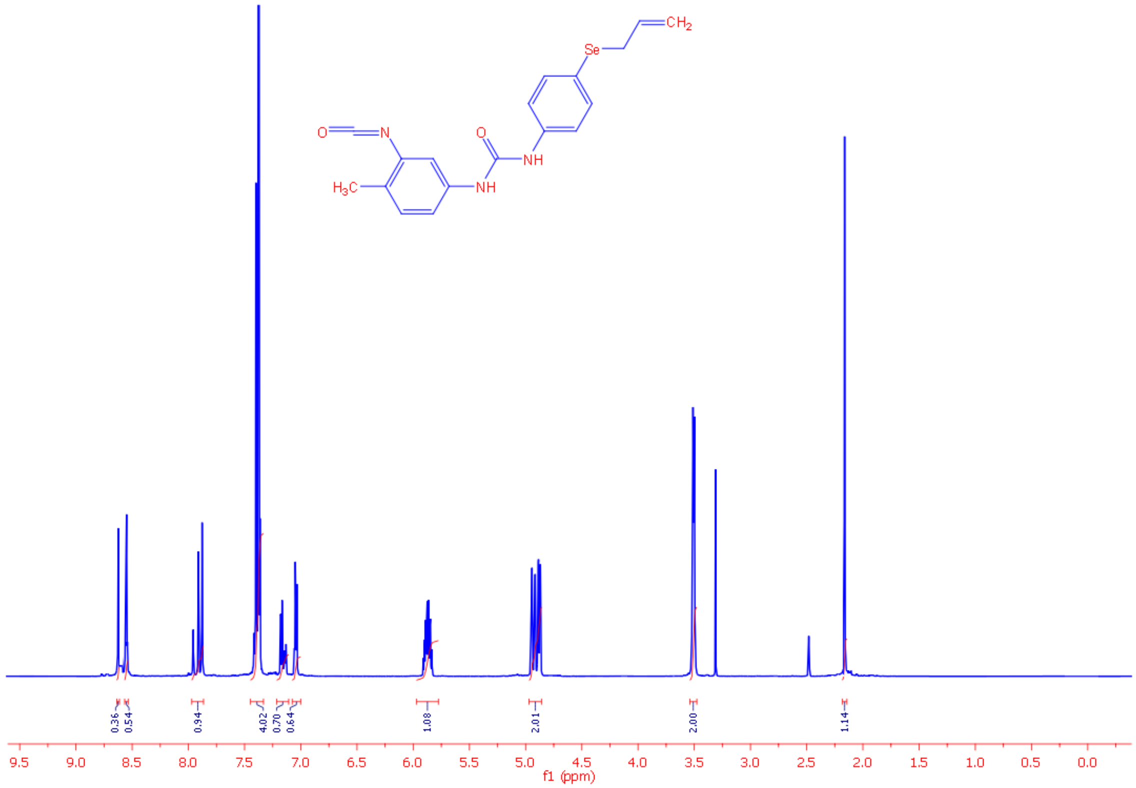

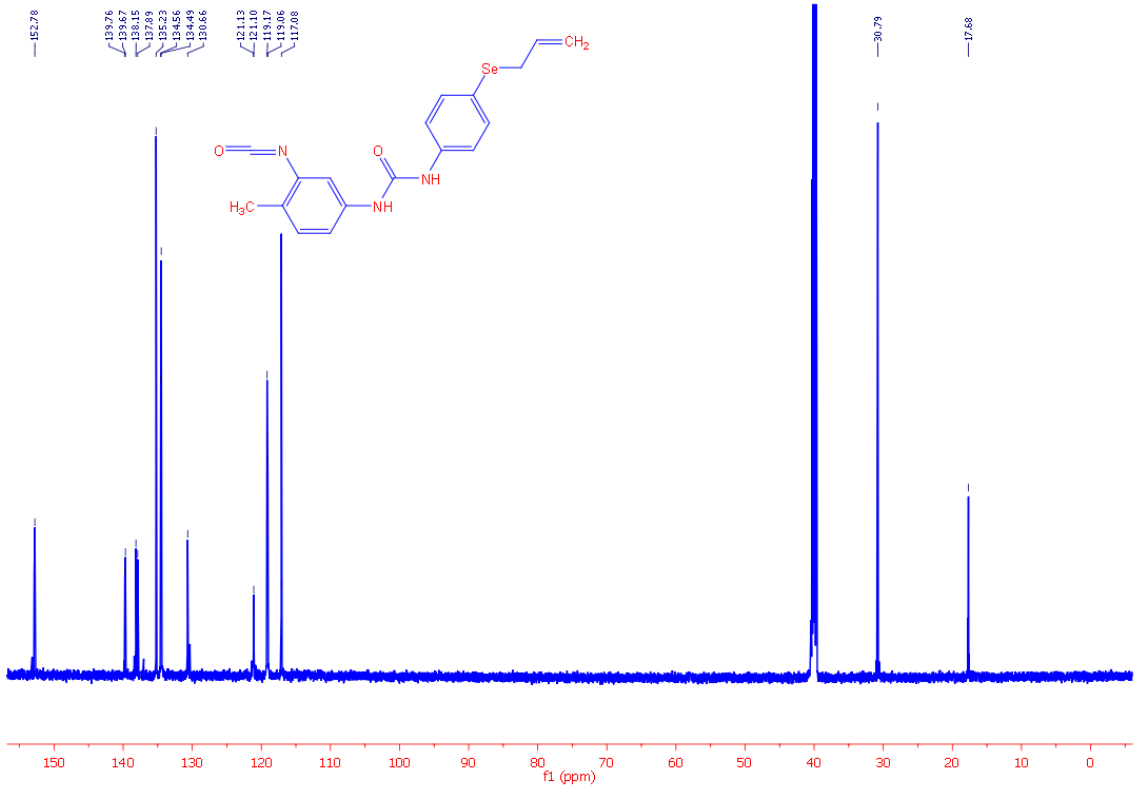

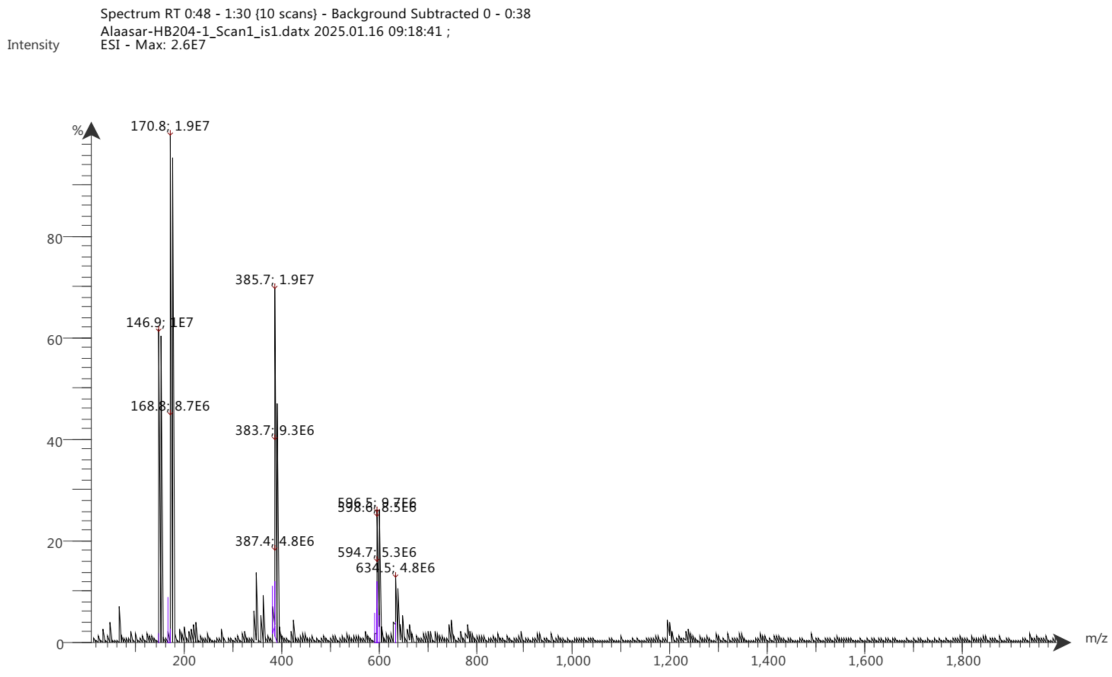
Spectrum RT 0:48 - 1:30 (10 scans) - Background Subtracted 0 - 0:38
Alaasar-HB204-1_Scan1_is1.datx 2025.01.16 09:18:41 ;
ESI - Max: 2.6E7
Intensity
%
170.8; 1.9E7
385.7; 1.9E7
146.9; 1E7
168.8; 8.7E6
383.7; 9.3E6
387.4; 4.8E6
594.7; 5.3E6
634.5; 4.8E6
m/z

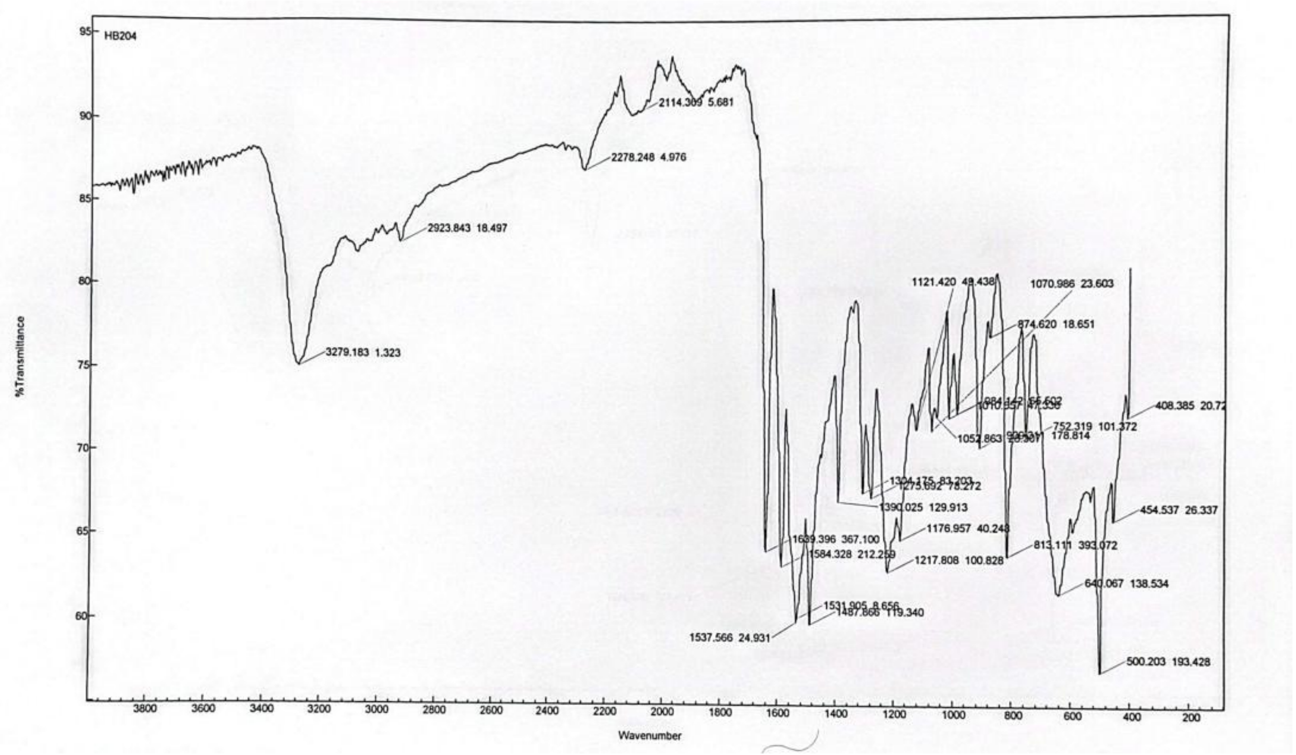
HB204
%Transmittance
Wavenumber
3279.183 1.323
2923.843 18.497
2278.248 4.976
2114.309 5.681
1121.420 48.438
1070.986 23.603
874.620 18.651
752.319 101.372
408.385 20.72
454.537 26.337
1390.025 129.913
1176.957 40.243
1639.396 367.100
1584.328 212.259
1217.808 100.828
813.111 393.072
640.067 138.534
1537.566 24.931
500.203 193.428
1052.863

1-(4-(benzylselanyl)phenyl)-3-(3-isocyanato-4-methylphenyl)urea (HB205)

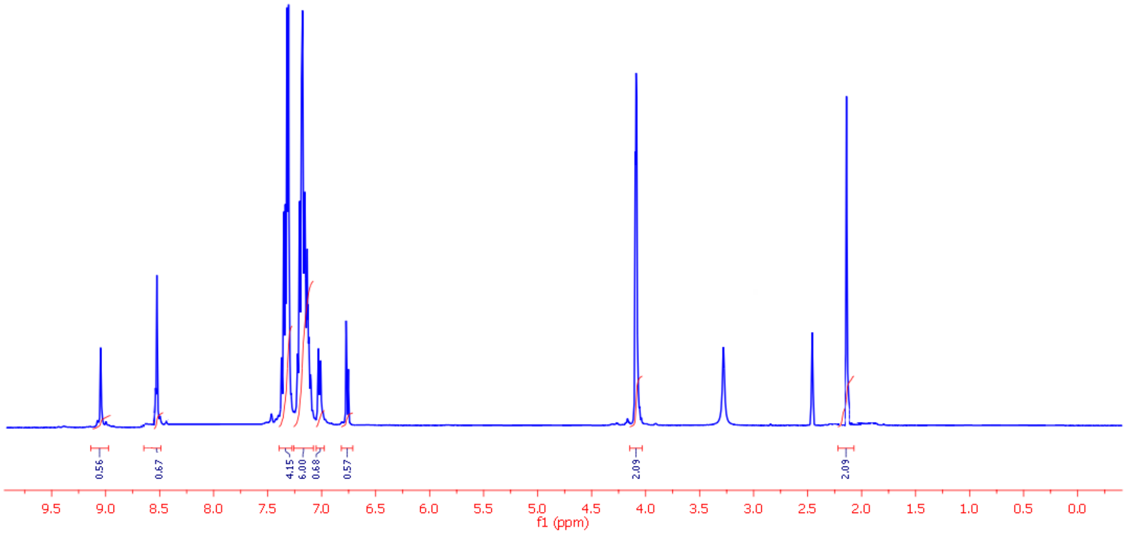

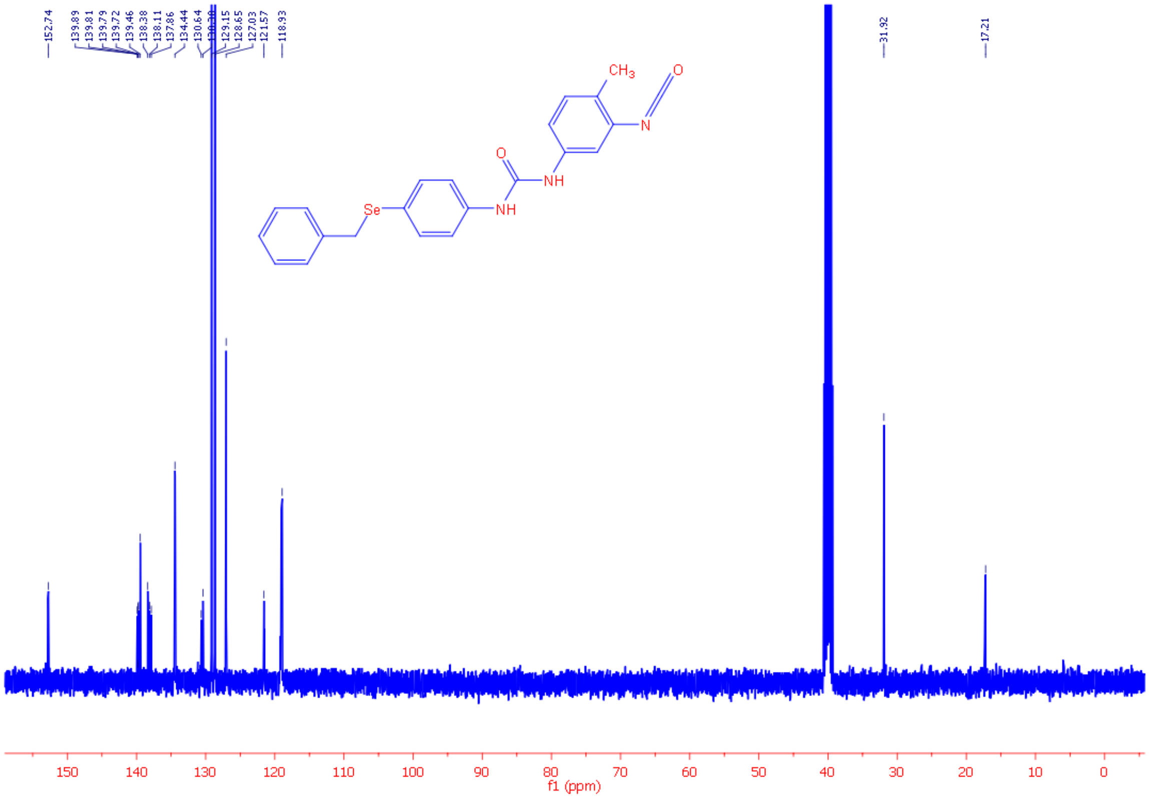

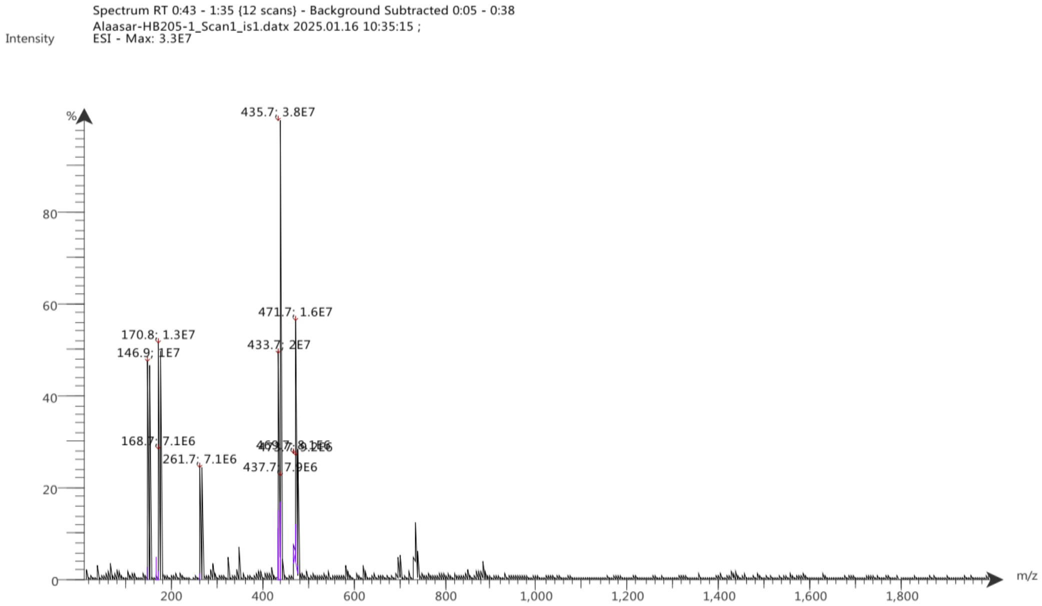
Spectrum RT 0:43 - 1:35 (12 scans) - Background Subtracted 0:05 - 0:38
Alaasar-HB205-1_Scan1_is1.datx 2025.01.16 10:35:15 ;
Intensity
ESI - Max: 3.3E7
%
435.7; 3.8E7
471.7; 1.6E7
170.8; 1.3E7
146.9; 1E7
433.7; 2E7
168.7; 7.1E6
261.7; 7.1E6
437.7; 7.9E6
m/z

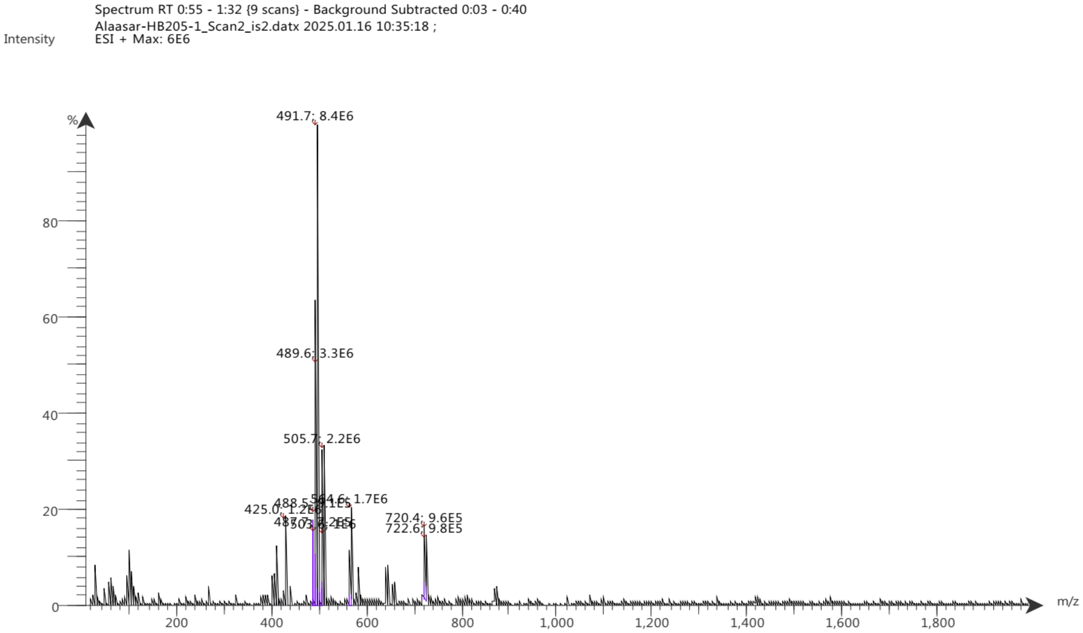
Spectrum RT 0:55 - 1:32 (9 scans) - Background Subtracted 0:03 - 0:40
Alaasar-HB205-1_Scan2_is2.datx 2025.01.16 10:35:18 ;
Intensity
ESI + Max: 6E6
%
491.7; 8.4E6
489.6; 3.3E6
505.7; 2.2E6
425.0; 1.2E6
720.4; 9.6E5
722.6; 9.8E5
m/z

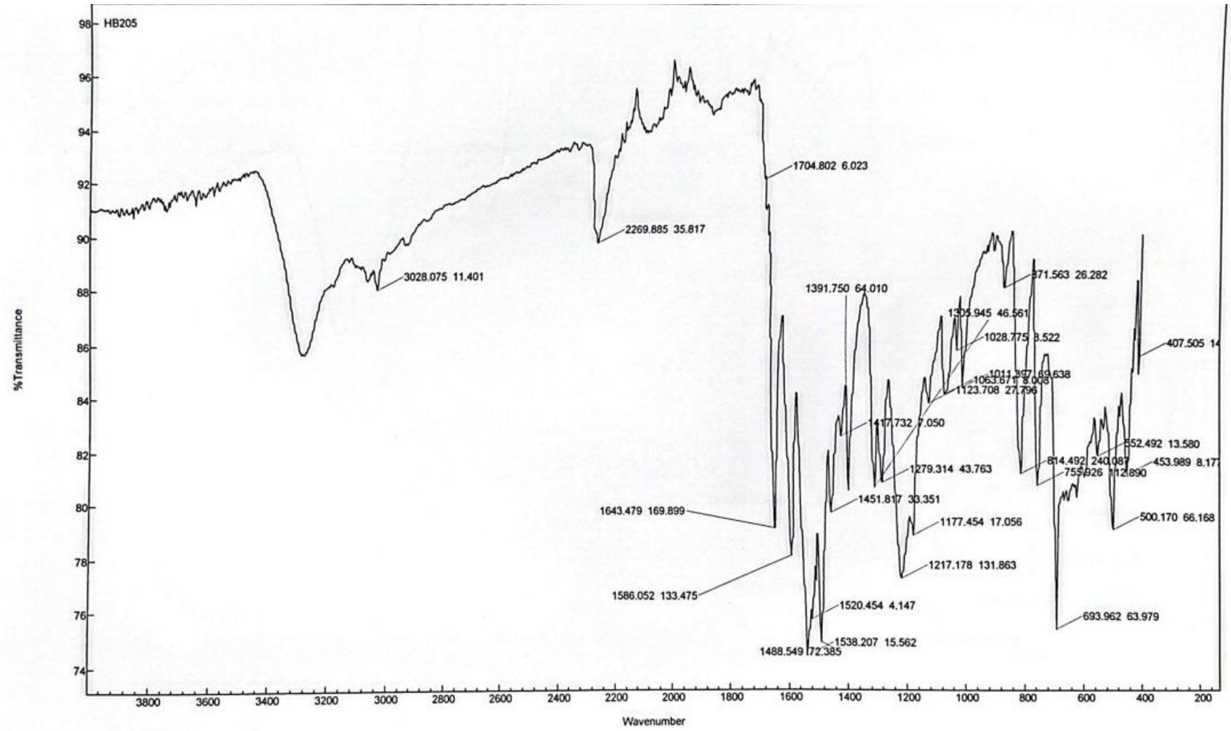
HB205
%Transmittance
Wavenumber
3028.075 11.401
2269.885 35.817
1704.802 6.023
1643.479 169.899
1586.052 133.475
1520.454 4.147
1538.207 15.562
1451.817 33.351
1391.750 64.010
1279.314 43.763
1217.178 131.863
1177.454 17.056
1123.708 27.796
1028.775 8.522
871.563 26.282
693.962 63.979
552.492 13.580
500.170 66.168

HPLC-Based Purity Assessment: Chromatograms of Synthesized Compounds (Absorbance at 254 nm *vs* Retention Time)

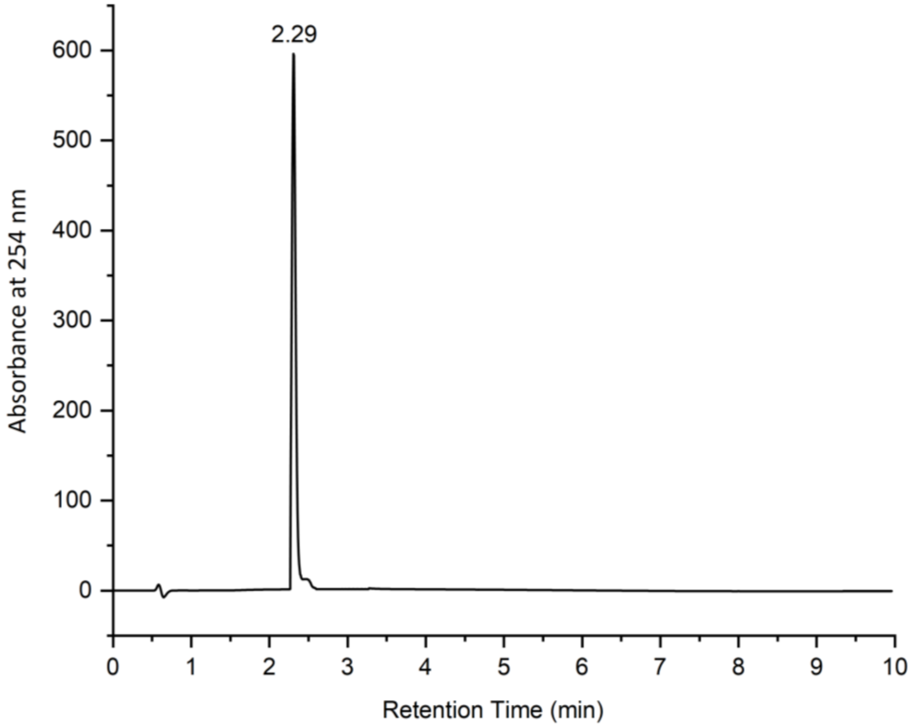

HPLC traces of purified HB188.

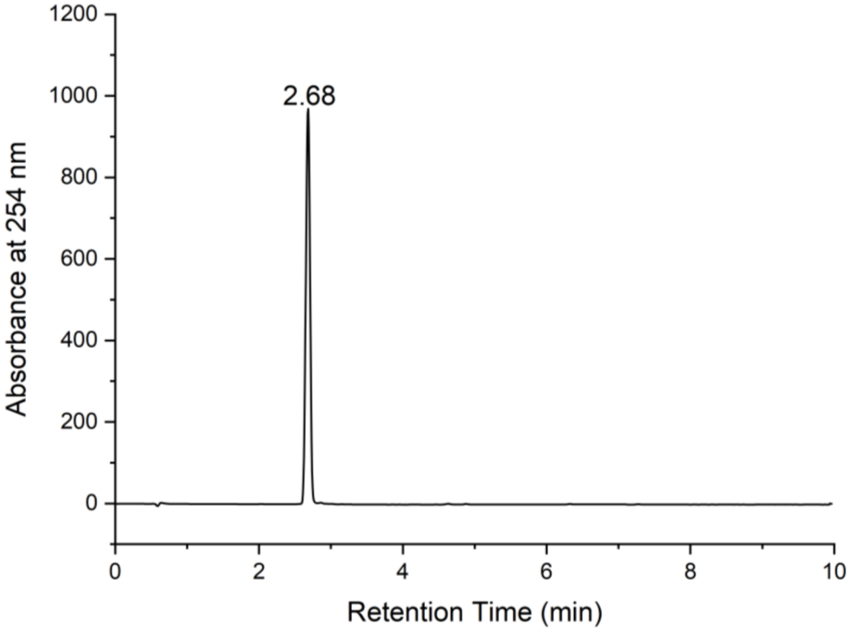

HPLC trace of purified HB190.

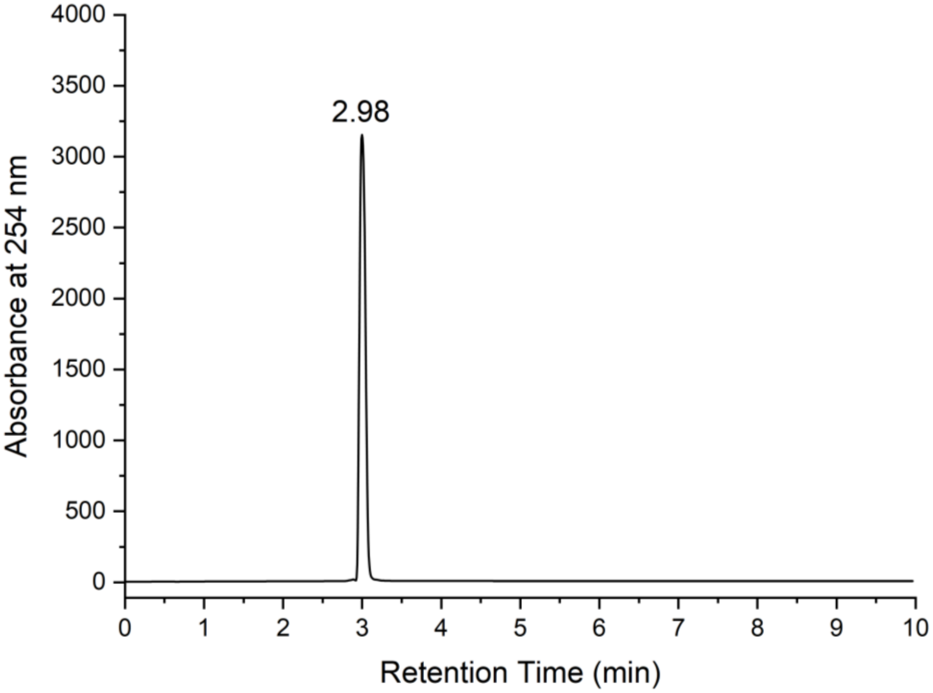

HPLC traces of purified HB198.

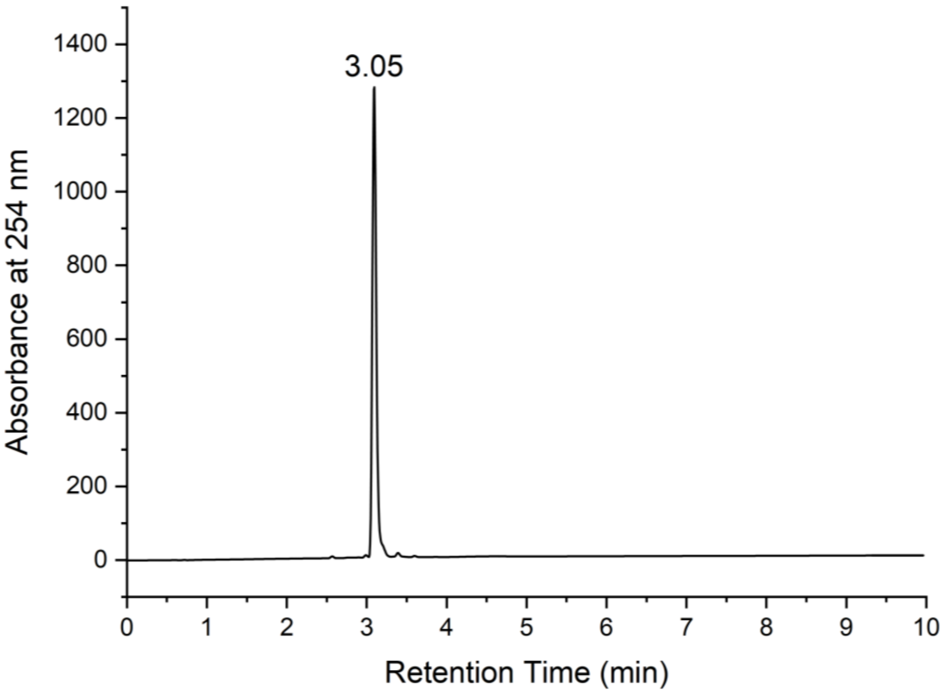

HPLC traces of purified HB204.

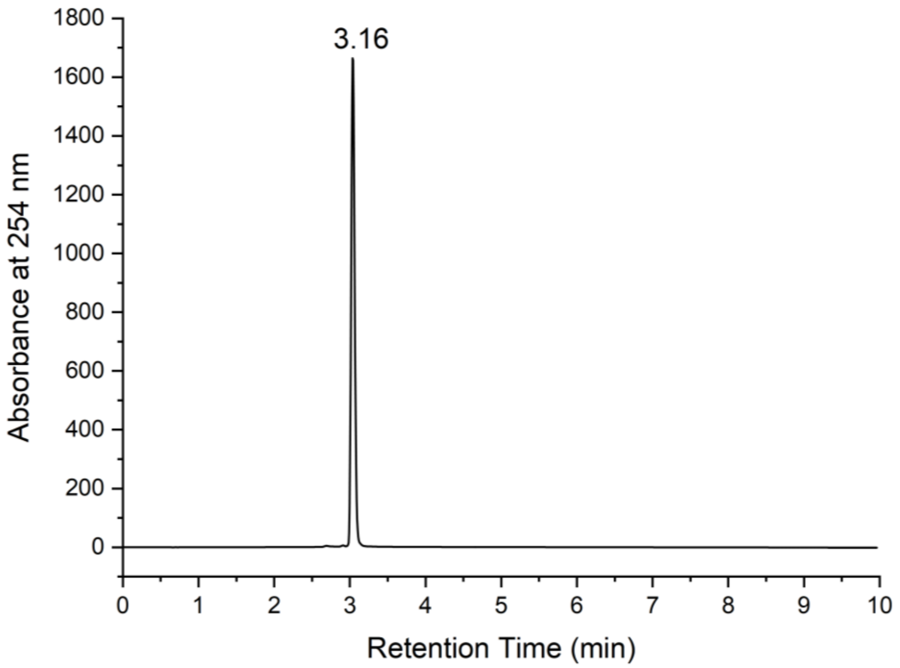

HPLC traces of purified HB193.

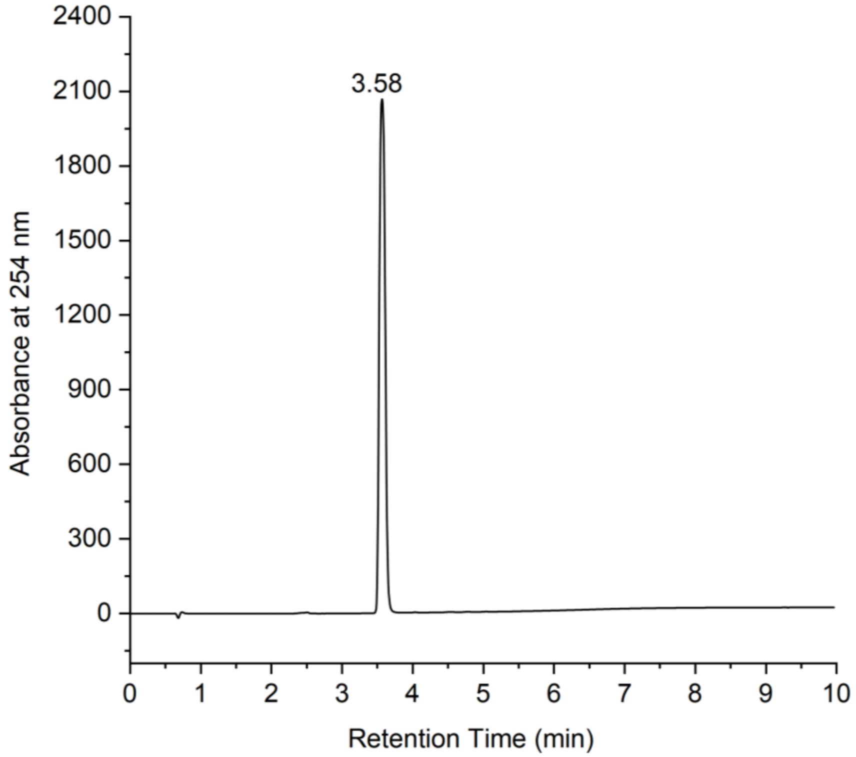

HPLC traces of purified HB200.

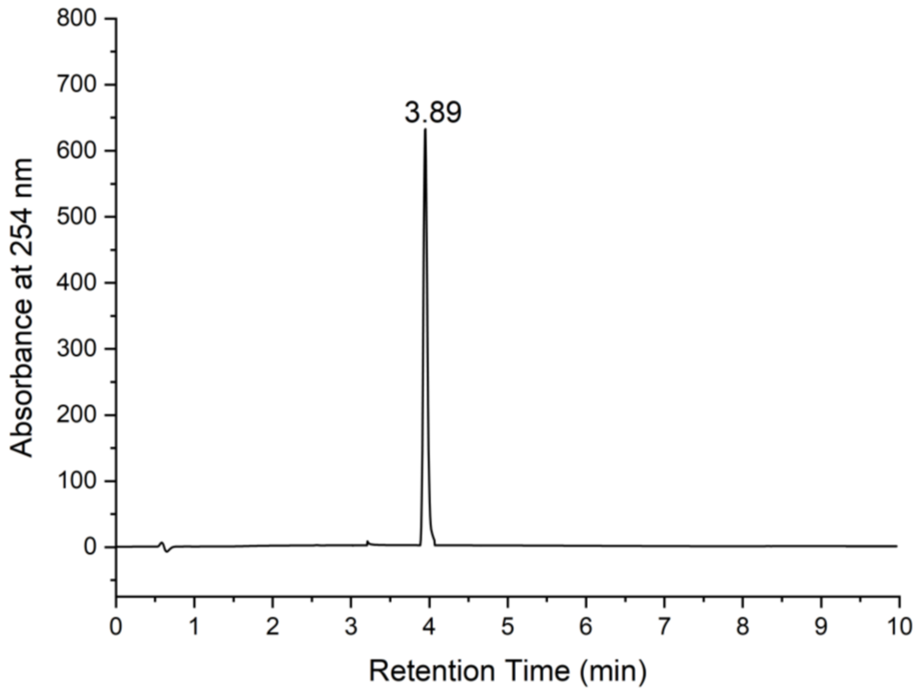

HPLC traces of purified HB199.

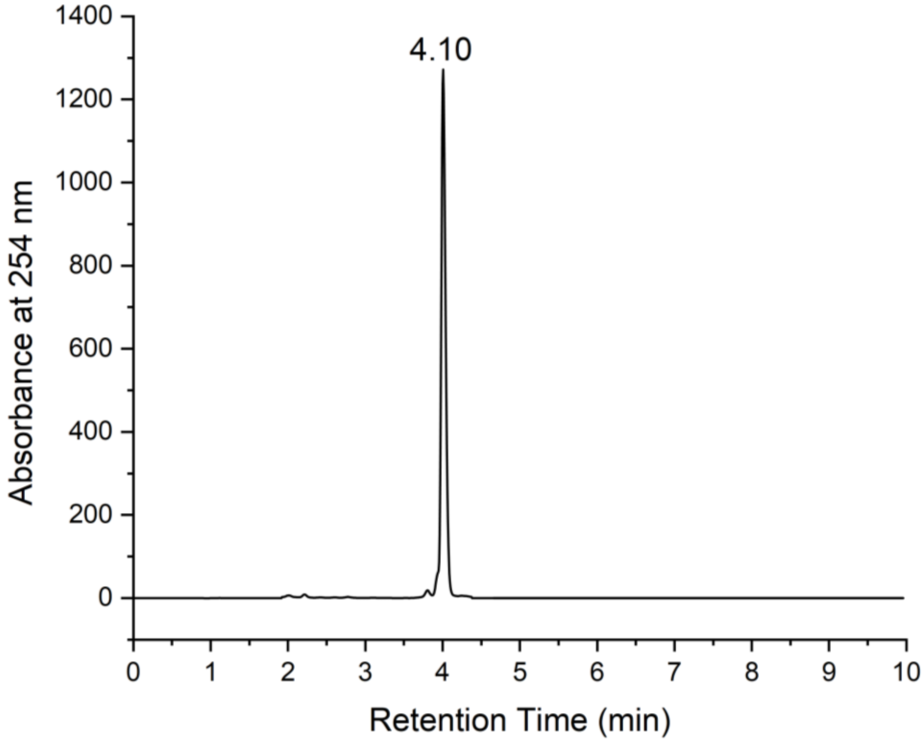

HPLC traces of purified HB203.

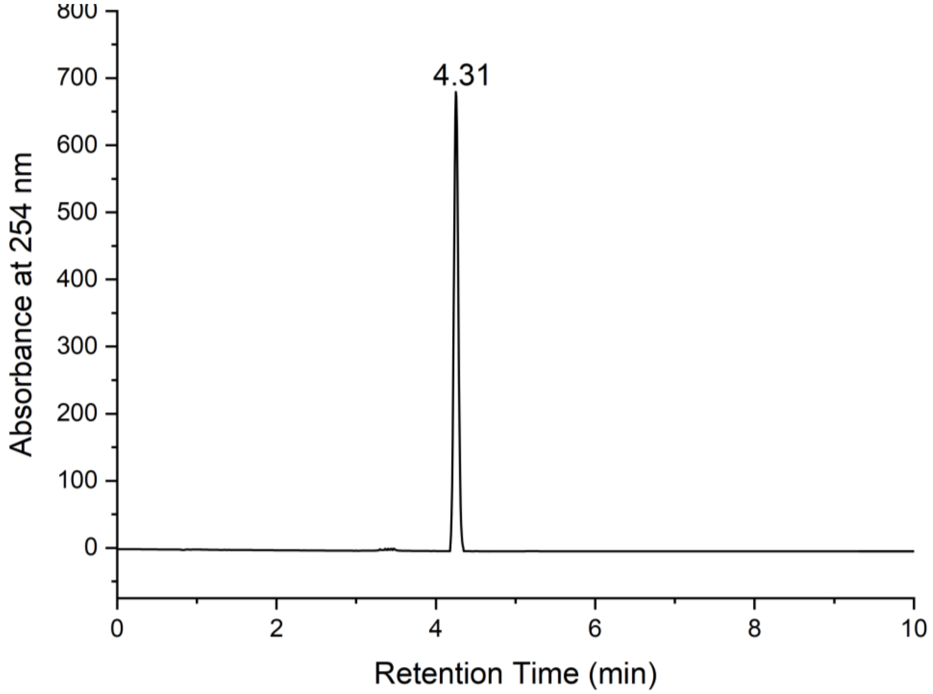

HPLC traces of purified HB202.

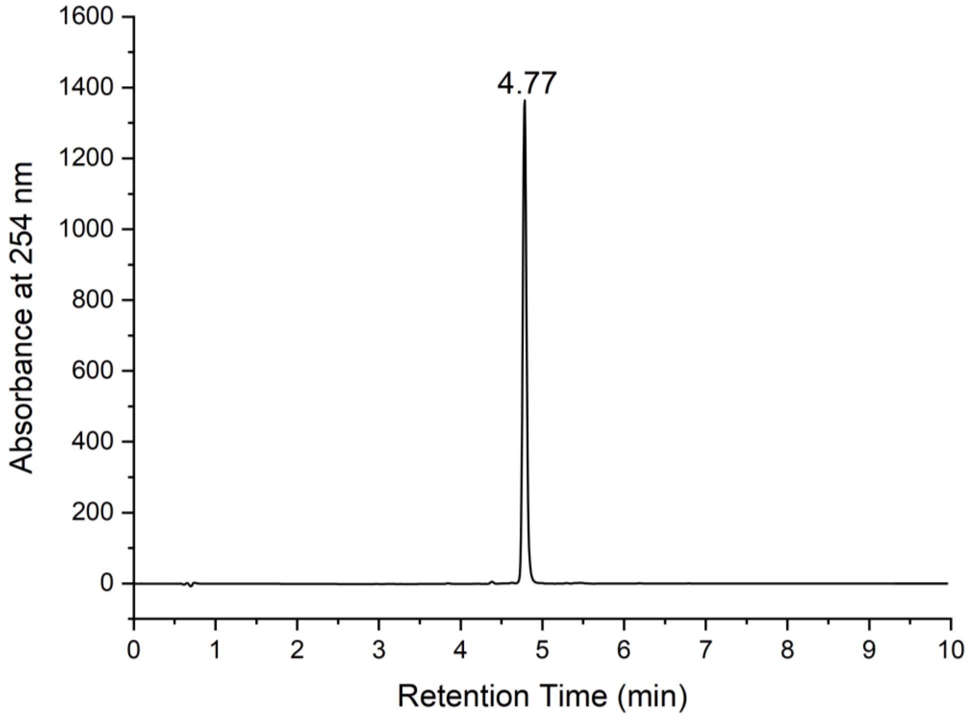

HPLC traces of purified HB206.

Biological Data

(A) HEPG2

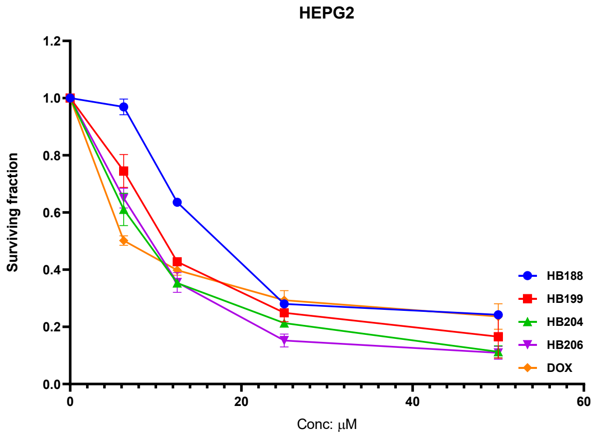

(B) A549

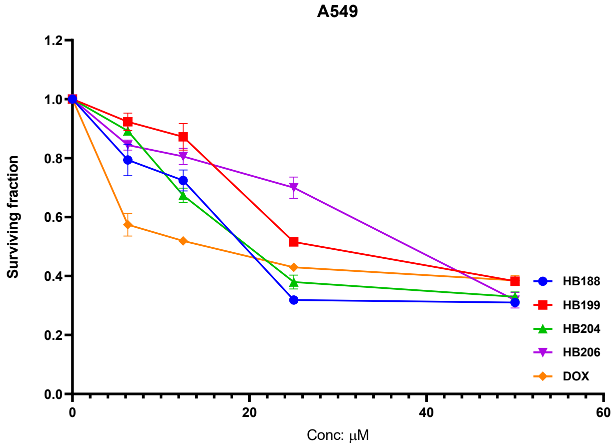

(C) HCT116

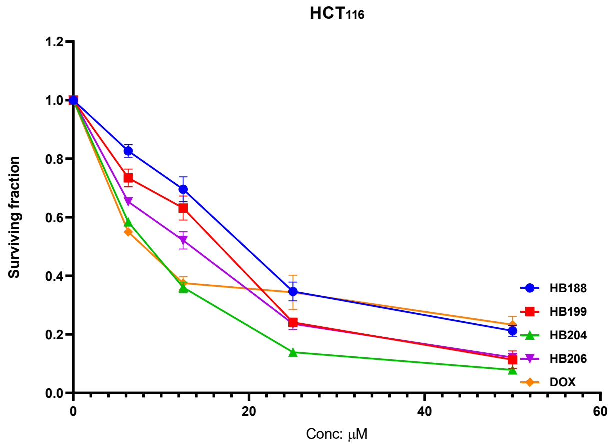

(D) HuH7

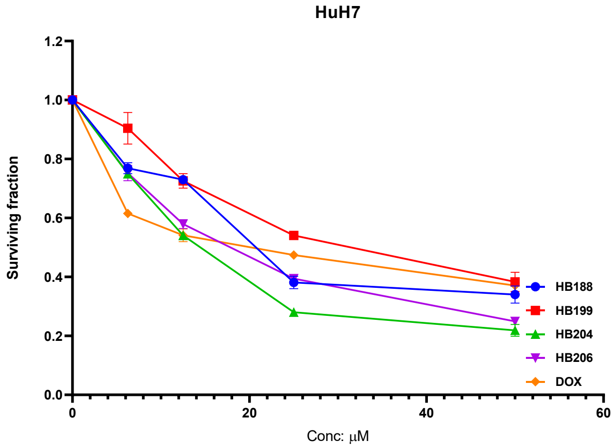

(E) FaDu

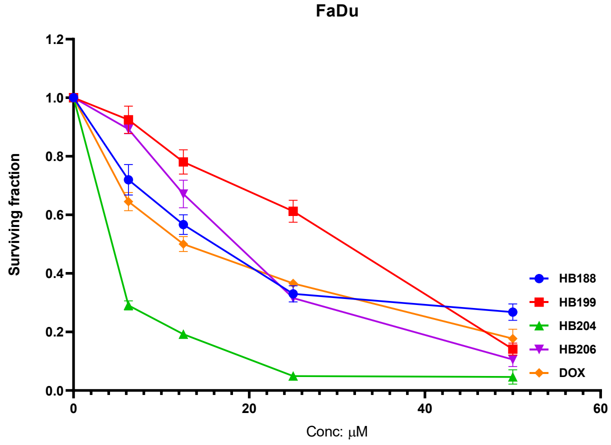

(F) MCF7

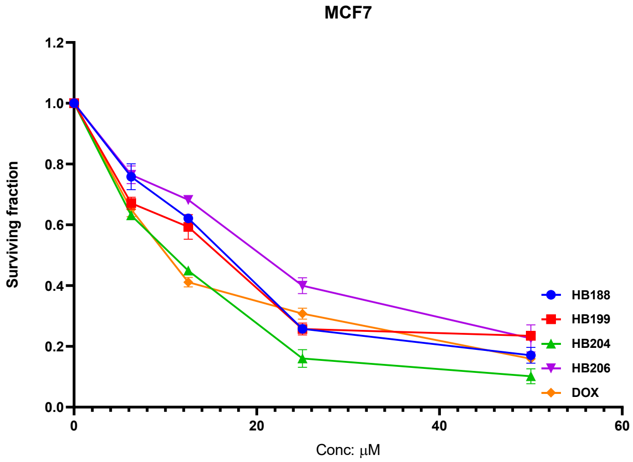

IC_{50} calculation curves of each examined cell line using the assessed compounds in detail.

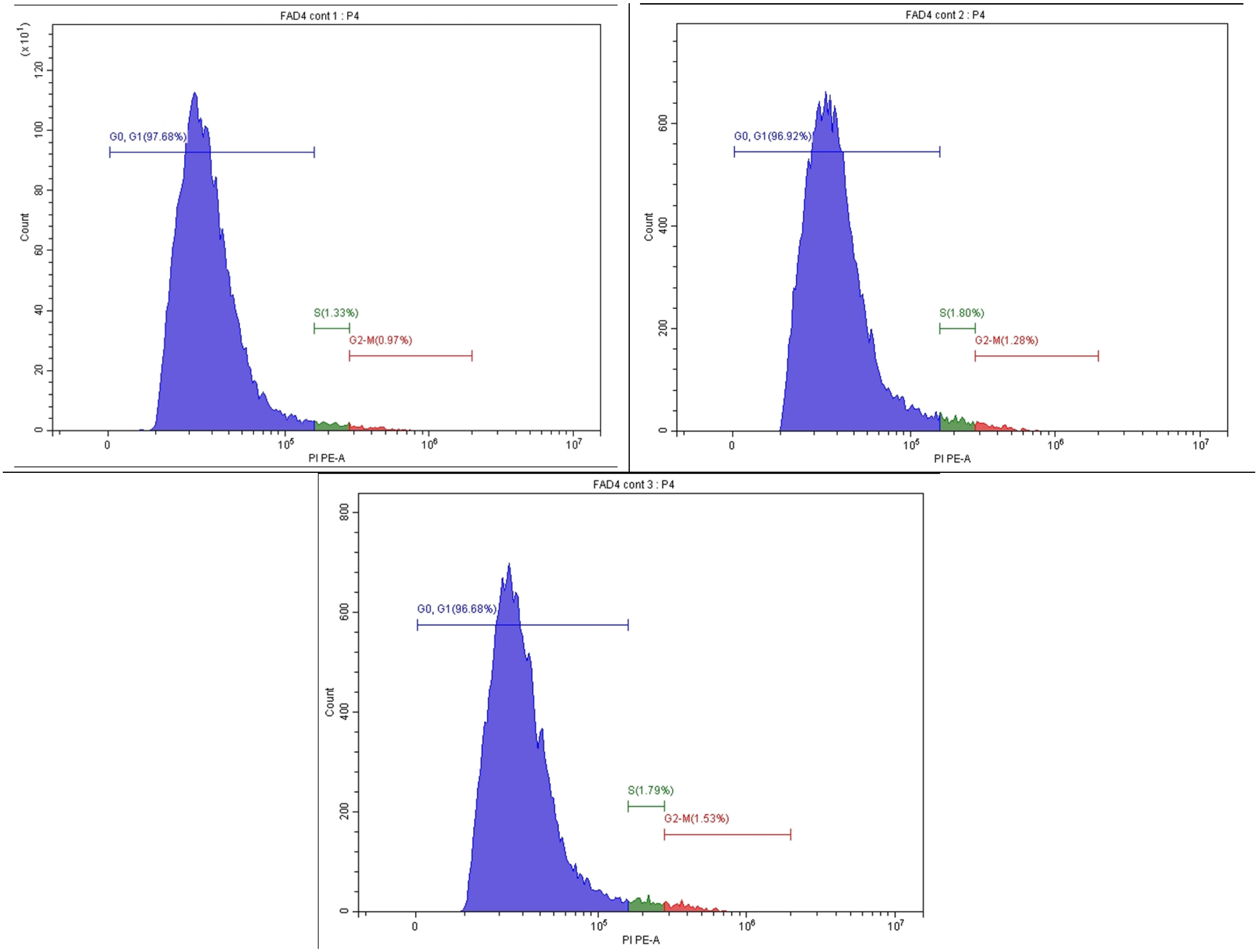

The triplicate experiments of the cell cycle analysis histograms of the untreated control FaDu cancer cell line.

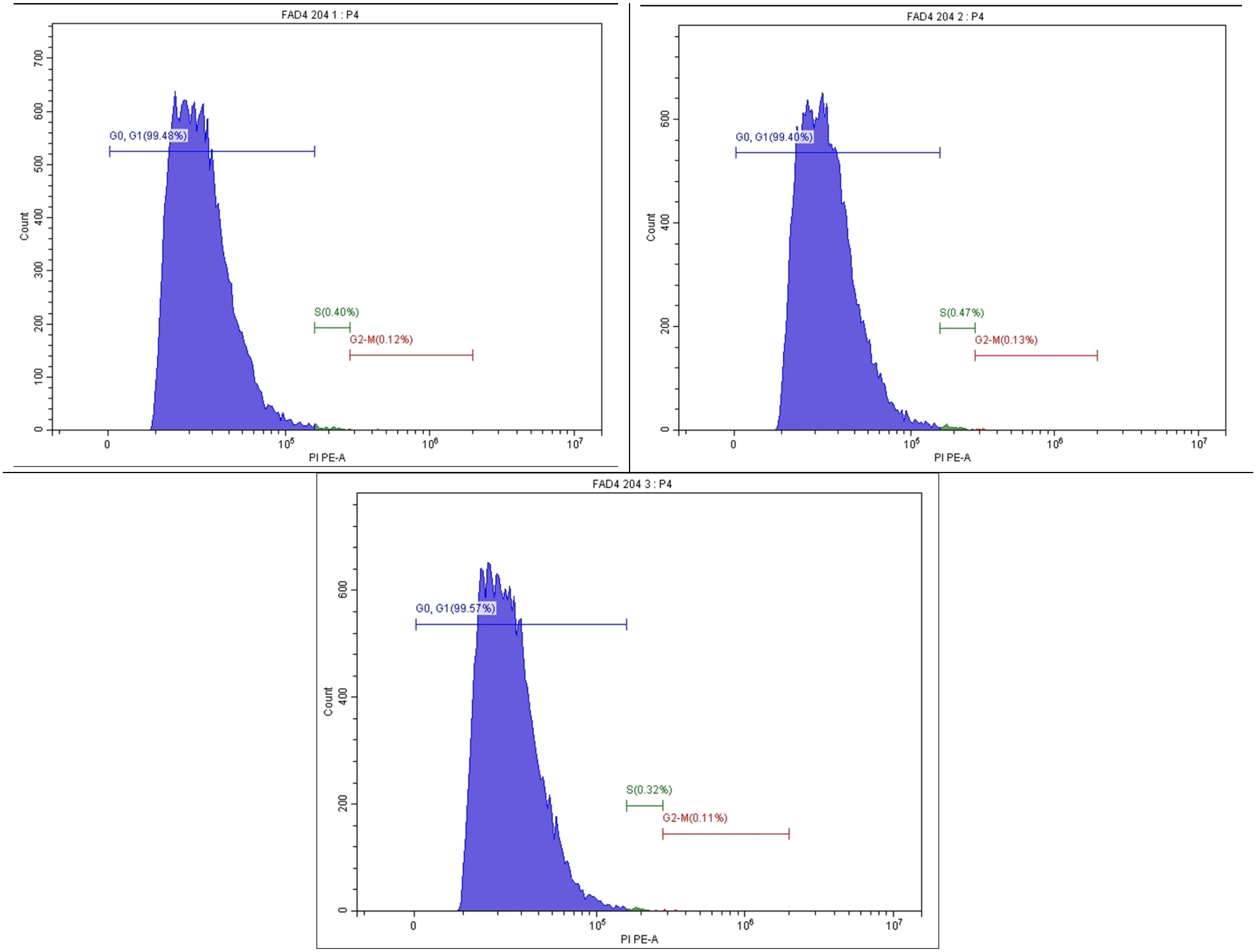

The triplicate experiments of the cell cycle analysis histograms of the HB204-treated FaDu cancer cell line.

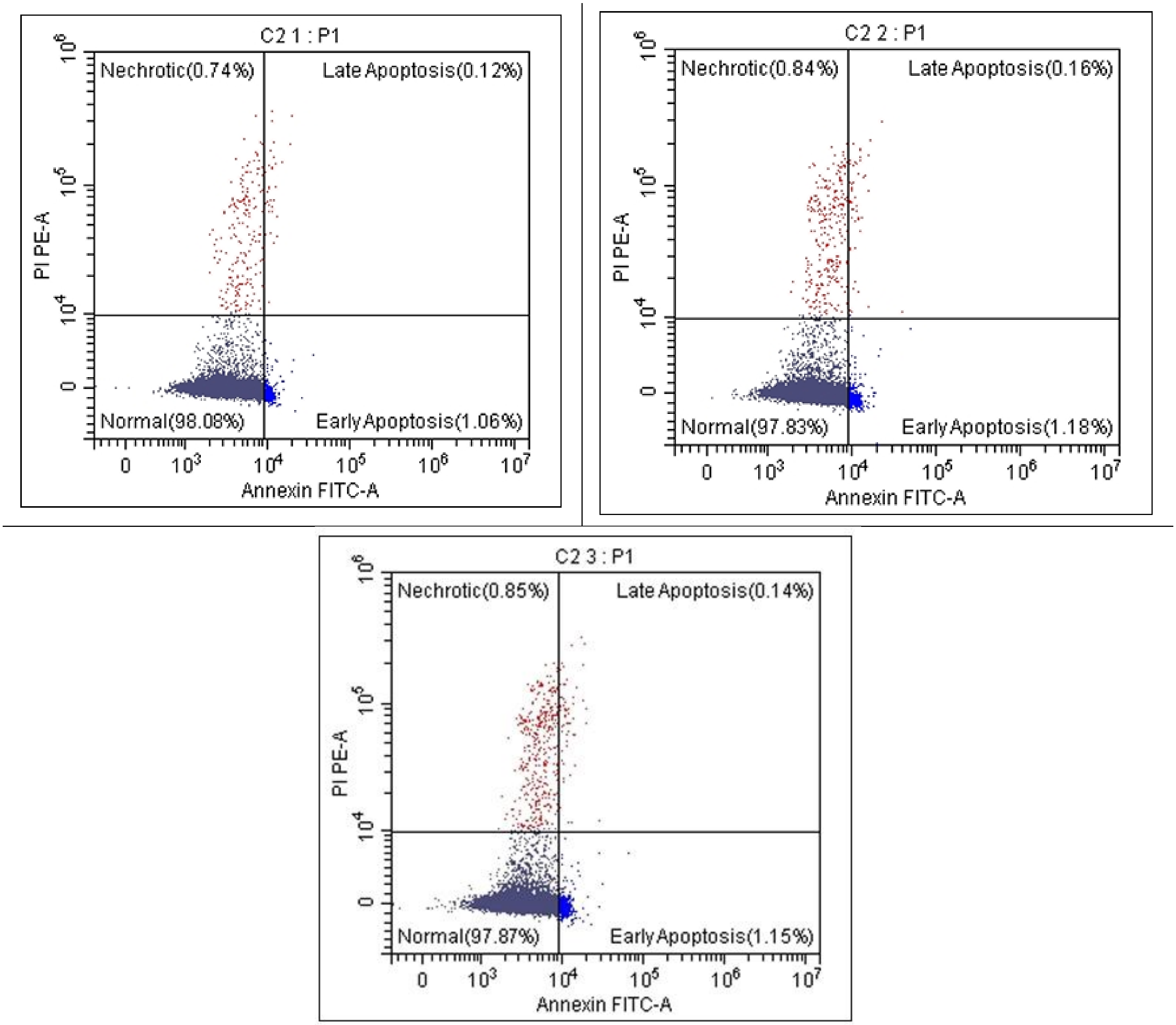

The triplicate experiments of the apoptosis analysis histograms of the untreated control FaDu cancer cell line.

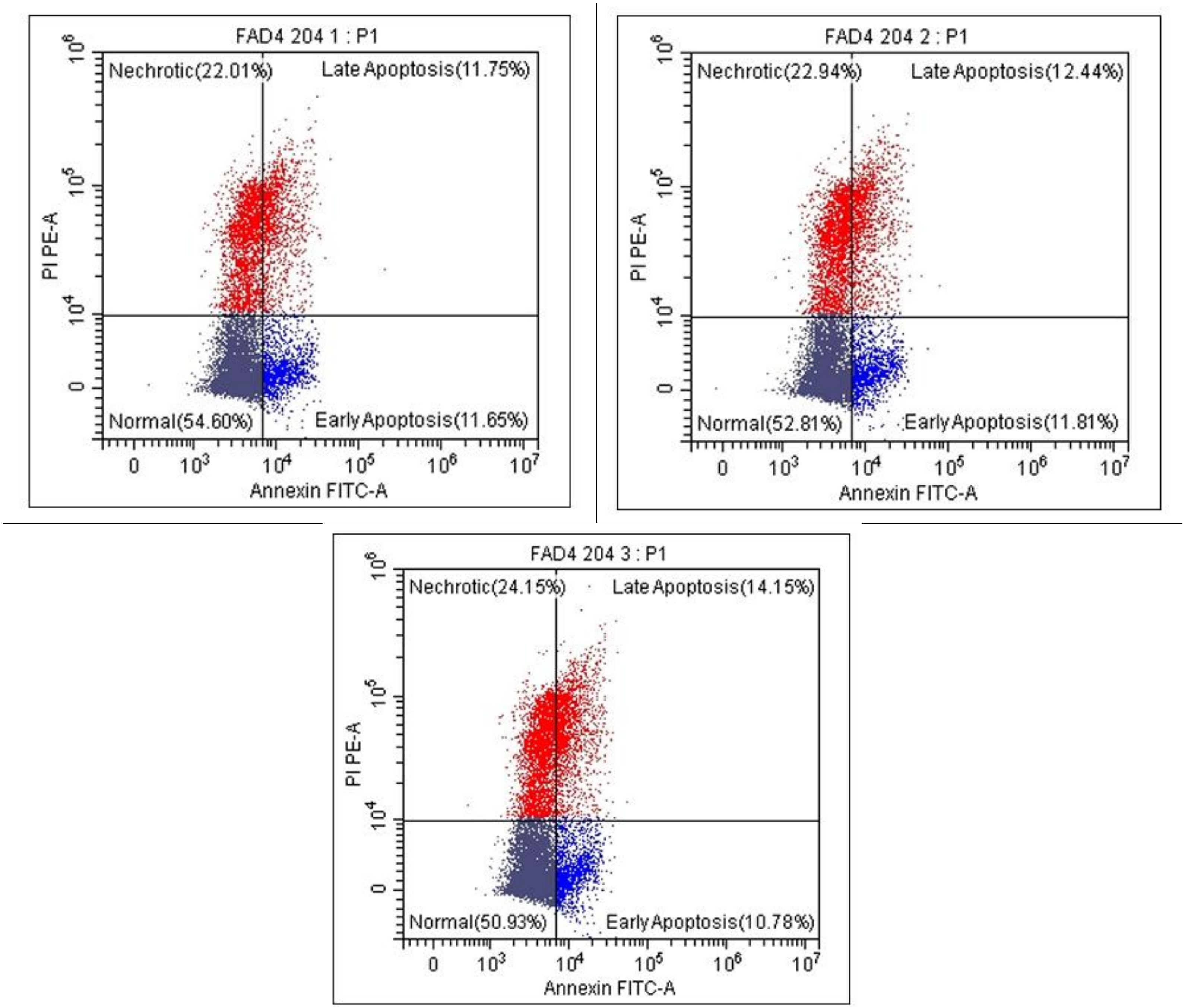

The triplicate experiments of the apoptosis analysis histograms of the HB204-treated FaDu cancer cell line.

Materials and Methods

SI1. % Inhibition against human eight cancer and two normal cell lines at 100 µg/mL

The antitumor activities of the new organoselenium compounds linked to urea against all tested cell lines, obtained from the ATCC (American Tissue Culture Collection), were evaluated by sulphorhodamine-B (SRB) assay [8]. Briefly, cells were seeded at a density of 3×10^3 cells/well in 96-well microtiter plates. They were left to attach for 24 h before incubation with the aforementioned compounds. Next, cells were treated with 100 µg/mL of the new organoselenium compounds linked to urea candidates.

For each concentration, three wells were used, and incubation was continued for 48 h. DMSO was used as a control vehicle (1 % v/v). At the end of incubation, cells were fixed with 20% trichloroacetic acid and stained with 0.4% SRB dye. The optical density (O.D.) of each well was measured spectrophotometrically at 570 nm using an ELISA microplate reader (TECAN sunrise™, Germany). The mean survival fraction at each drug concentration was calculated as follows: O.D. of the treated cells/O.D. of the control cells.

SI2. Cytotoxicity evaluation against HEPG2, A549, HCT_{116}, HuH7, FaDu, and MCF7 cancer cell lines

The antitumor activities of the new organoselenium compounds linked to urea against HEPG2, A549, HCT116, HuH7, FaDu, and MCF7 cells were evaluated by sulphorhodamine-B (SRB) assay [8]. Briefly, cells were seeded at a density of 3×10^3 cells/well in 96-well microtiter plates. They were left to attach for 24 h before incubation with the aforementioned compounds. Next, cells were treated with different concentrations (62.5, 12.5, 25, and 50 µg/mL) of the new organoselenium compounds linked to urea.

For each concentration, three wells were used, and incubation was continued for 48 h. DMSO was used as a control vehicle (1 % *v/v*). At the end of incubation, cells were fixed with 20% trichloroacetic acid and stained with 0.4% SRB dye. The optical density (O.D.) of each well was measured spectrophotometrically at 570 nm using an ELISA microplate reader (TECAN sunrise™, Germany). The mean survival fraction at each drug concentration was calculated as follows: O.D. of the treated cells/O.D. of the control cells. The IC_{50} (concentration that produces

50% cell growth inhibition) value of each drug was calculated using sigmoidal dose-response curve-fitting models (GraphPad Prism software, version 8).

SI3. Molecular dynamics (MD) simulations

The molecular dynamics simulations were carried out using the Desmond simulation package of Schrödinger LLC.[9-11] Each docked pose served as the starting point for all calculations; the system was built and relaxed, and we then proceeded with the simulation. The NPγT ensemble with a temperature of 300 K and a pressure of 1.01 bar was applied in all runs. The simulation length was 500 ns with a relaxation time of 1 ps. The OPLS3 force field parameters were used in all simulations.[12] The cutoff radius in Coulomb interactions was 9.0 Å. The orthorhombic periodic box boundaries were set 10 Å away from the protein atoms. The water molecules were explicitly described using the transferable intermolecular potential (TIP3P) model.[13] Salt concentration was set to 0.15 M NaCl and was built using the System Builder utility of Desmond. The Martyna−Tuckerman−Klein chain coupling scheme with a coupling constant of 2.0 ps was used for the pressure control, and the Nosé−Hoover chain coupling scheme for the temperature control.[14, 15] Nonbonded forces were calculated using a RESPA integrator, with short-range forces updated at each step and long-range forces at every third step. The trajectories were saved at 300 ps intervals for analysis. The behavior and interactions between the ligands and protein were analyzed using the Simulation Interaction Diagram tool implemented in the Desmond MD package. The stability of MD simulations was monitored by looking at the RMSD of the ligand and protein atom positions as a function of simulation time.

References

1. H. M. Abd El-Lateef, M. M. Khalaf, M. Gouda, K. Shalabi, F. E. T. Heakal, A. S. Al-Janabi and S. Shaaban, *Construction and Building Materials*, 2023, **366**, 130135.
2. S. Shaaban, K. T. Abdullah, K. Shalabi, T. A. Yousef, O. K. Al Duaij, G. M. Alsulaim, H. A. Althikrallah, M. Alaasar, A. S. Al-Janabi and A. M. Abu-Dief, *Applied Organometallic Chemistry*, 2024, e7712.
3. S. Shaaban, A. M. Abu-Dief, M. Alaasar, A. S. Al-Janabi, N. S. Alsadun, O. K. Al Duaij and T. A. Yousef, *Applied Organometallic Chemistry*, 2025, **39**, e7776.
4. S. Shaaban, A. M. Abu-Dief, M. Alaasar, A. S. Al-Janabi, N. S. Alsadun, O. K. Al Duaij and T. A. Yousef, *Applied Organometallic Chemistry*, 2024, e7776.
5. S. Shaaban, A. Negm, M. A. Sobh and L. A. Wessjohann, *European journal of medicinal chemistry*, 2015, **97**, 190-201.
6. S. Shaaban, M. S. S. Adam and N. M. El-Metwaly, *Journal of Molecular Liquids*, 2022, **363**, 119907.
7. S. Shaaban, A. M. Ashmawy, A. Negm and L. A. Wessjohann, *European journal of medicinal chemistry*, 2019, **179**, 515-526.
8. P. Skehan, R. Storeng, D. Scudiero, A. Monks, J. McMahon, D. Vistica, J. T. Warren, H. Bokesch, S. Kenney and M. R. Boyd, *JNCI: Journal of the National Cancer Institute*, 1990, **82**, 1107-1112.
9. K. J. Bowers, D. E. Chow, H. Xu, R. O. Dror, M. P. Eastwood, B. A. Gregersen, J. L. Klepeis, I. Kolossvary, M. A. Moraes, F. D. Sacerdoti, J. K. Salmon, Y. Shan and D. E. Shaw, 2006.
10. M. H. El-Shershaby, A. Ghiaty, A. H. Bayoumi, A. A. Al-Karmalawy, E. M. Husseiny, M. S. El-Zoghbi and H. S. Abulkhair, *Bioorganic & Medicinal Chemistry*, 2021, **42**, 116266.
11. D. E. S. Research, *Journal*, 2021.
12. E. Harder, W. Damm, J. Maple, C. Wu, M. Reboul, J. Y. Xiang, L. Wang, D. Lupyan, M. K. Dahlgren, J. L. Knight, J. W. Kaus, D. S. Cerutti, G. Krilov, W. L. Jorgensen, R. Abel and R. A. Friesner, *Journal of Chemical Theory and Computation*, 2016, **12**, 281-296.
13. W. L. Jorgensen, J. Chandrasekhar, J. D. Madura, R. W. Impey and M. L. Klein, *Journal of Chemical Physics*, 1983, **79**, 926-935.
14. G. J. Martyna, M. L. Klein and M. Tuckerman, *Journal of Chemical Physics*, 1992, **97**, 2635-2643.
15. G. J. Martyna, D. J. Tobias and M. L. Klein, *Journal of Chemical Physics*, 1994, **101**, 4177-4189.
